# An integrated approach of immunogenomics and bioinformatics to identify new Tumor Associated Antigens (TAA) for mammary cancer immunological prevention

**DOI:** 10.1186/1471-2105-6-S4-S7

**Published:** 2005-12-01

**Authors:** Federica Cavallo, Annalisa Astolfi, Manuela Iezzi, Francesca Cordero, Pier-Luigi Lollini, Guido Forni, Raffaele Calogero

**Affiliations:** 1 Dept. of Clinical and Biological Sciences, University of Torino, Az. Ospedaliera S. Luigi, Regione Gonzole 10, I-10043 Orbassano Italy; 2 Dept. of Informatics, University of Torino, Via Pessinetto 12, I-10100 Torino, Italy; 3 Dept. of Experimental Pathology, University of Bologna, Viale Filopanti 22, I-40126 Bologna, Italy; 4 Dept. of Oncology and Neurosciences, University of Chieti, Via Colle dell'Ara, I-66013 Chieti, Italy

## Abstract

**Background:**

Neoplastic transformation is a multistep process in which distinct gene products of specific cell regulatory pathways are involved at each stage. Identification of overexpressed genes provides an unprecedented opportunity to address the immune system against antigens typical of defined stages of neoplastic transformation. HER-2/neu/ERBB2 (Her2) oncogene is a prototype of deregulated oncogenic protein kinase membrane receptors. Mice transgenic for rat Her2 (BALB-neuT mice) were studied to evaluate the stage in which vaccines can prevent the onset of Her2 driven mammary carcinomas. As Her2 is not overexpressed in all mammary carcinomas, definition of an additional set of tumor associated antigens (TAAs) expressed at defined stages by most breast carcinomas would allow a broader coverage of vaccination. To address this question, a meta-analysis was performed on two transcription profile studies [1,2] to identify a set of new TAA targets to be used instead of or in conjunction with Her2.

**Results:**

The five TAAs identified (Tes, Rcn2, Rnf4, Cradd, Galnt3) are those whose expression is linearly related to the tumor mass increase in BALB-neuT mammary glands. Moreover, they have a low expression in normal tissues and are generally expressed in human breast tumors, though at a lower level than Her2.

**Conclusion:**

Although the number of putative TAAs identified is limited, this pilot study suggests that meta-analysis of expression profiles produces results that could assist in the designing of pre-clinical immunopreventive vaccines.

## Background

One of the most significant tenet of tumor immunology is the assumption that the immune system is able to discriminate between normal and neoplastic tissues. As this distinction is based on the overexpression of TAAs, the discovery of TAAs and their molecular and genetic characterization is important in both tumor immunodiagnosis and immunotherapy.

The progressive elucidation of the nature of TAAs recognized by antibodies and T lymphocytes coupled with the elaboration of methods for their isolation and synthesis of their peptides is leading towards new formulations of antitumor vaccines. Despite of the fact that long lists of well-characterized TAAs recognized by T cells [3] and by antibodies [4] are currently available, the attempts to use them to arouse an immune response able to cure tumor patients has met with very little success, at least so far [5]. Towards this goal significant obstacles are posed by the patient's state of immunosuppression and the numerous mechanisms to evade the immune response that a tumor progressively acquires [6]. This two order of obstacles might suggest that active immunotherapy is not very appropriate for patients with advanced tumors.

A more modern endeavor is to exploit the potential of the immune response elicited by a vaccine against more plausible targets, such as pre-neoplastic lesions. In this case, the immune response may be addressed towards TAAs that are already present in early lesions and that remain overexpressed during the progression of the tumor [7]. Identification of TAAs on preneoplastic cells will provide the opportunity to trigger the immune system against transformed cells at their earliest manifestation [8]. The availability of animal models of autochthonous carcinogenesis offers an unprecedented opportunity to identify this kind of TAAs and to test the efficacy of the immune control of preneoplastic lesions [9]. A preneoplastic lesion formed by few cells characterized by an indolent proliferation both displays a limited genetic instability and is more permeable to an immune attack than a large tumor [10,11]. In addition it is possible that early expressed TAAs are more suitable target for tumor immune prevention than most of the already defined TAAs.

The characterization of TAAs expressed in early lesions is a prerequisite for setting up protocols for tumor prevention instead of for tumor cure [12]. The protein product of the oncogene Her2 [13], IGF-R [14] and cyclin B1 [15] are significant examples of early expressed TAAs causally involved in the progression of the lesions [7].

Mouse strains transgenic for oncogenes or knock-out for oncosuppressor genes that consequently develop defined kind of tumors are being used for preclinical assessment of the potential of preventive vaccines [16,11].

By combining the information provided by the genome sequence and the expression profiles of entire transcriptomes (microarrays), these mouse models can also lead towards the identification of TAAs expressed during progressive stages of carcinogenesis. Microarrays are an attractive way of identifying new TAAs since their procedure is now firmly established [17] and they are well suited to the intrinsic limitations of the biological material to be examined (e.g. the small amount of starting material). This paper describes our application of meta-analysis to two BALB-neuT mice transcription profiling studies [1,2] to evaluate the possibility of identifying, within the tumor modulated genes, a set of new putative targets to be used as TAAs instead of or in conjunction with Her2.

## Results and discussion

Our previous demonstration of persistent inhibition of autochthonous preneoplastic lesions in a mouse model of Her2 mammary carcinogenesis by a combined DNA and cell vaccine [1], and the curing of Her2 transplantable tumors by DNA vaccination [18] indicates that efficient immunological inhibition of Her2 carcinogenesis can be achieved. We have also shown that two transcriptional profiling studies based on different immunization protocols in BALB-neuT mice [1,2] are integrable [17]. Since an important issue of DNA vaccination is the definition of a set of target genes other that Her2 in order to broaden its effective coverage, we decided to use these data to identify a set of new TAAs to be used in DNA vaccination.

### Datasets

Seven prototypic situations were evaluated. Mammary glands from: (a) 6-week-old mice (wk6nt), displaying atypical hyperplasia; (b) 10-week-old mice (wk10nt), showing atypical hyperplasia and some foci of in situ carcinomas; (c) 15-week-old mice (wk15nt), with diffuse hyperplasia and in situ carcinomas; (d) 19-week-old mice (wk19nt), displaying lobular invasive carcinomas; (e) 22-week-old mice (wk22nt), with palpable carcinomas; (f) 26-week-old mice (wk26nt), with large lobular and metastasizing carcinomas, (g) 2-week-pregnant (wk2prg) wild type BALB/c mice displaying marked pregnancy-related hyperplasia [19,1]. wk6nt, wk15nt, wk19nt and wk26nt gland expression profiles were derived from Astolfi [2], wk10nt, wk22nt and wk2prg gland profiles from Quaglino [1].

### Selection of genes whose expression is linearly correlated with the increase of the tumor

**Figure 1:**
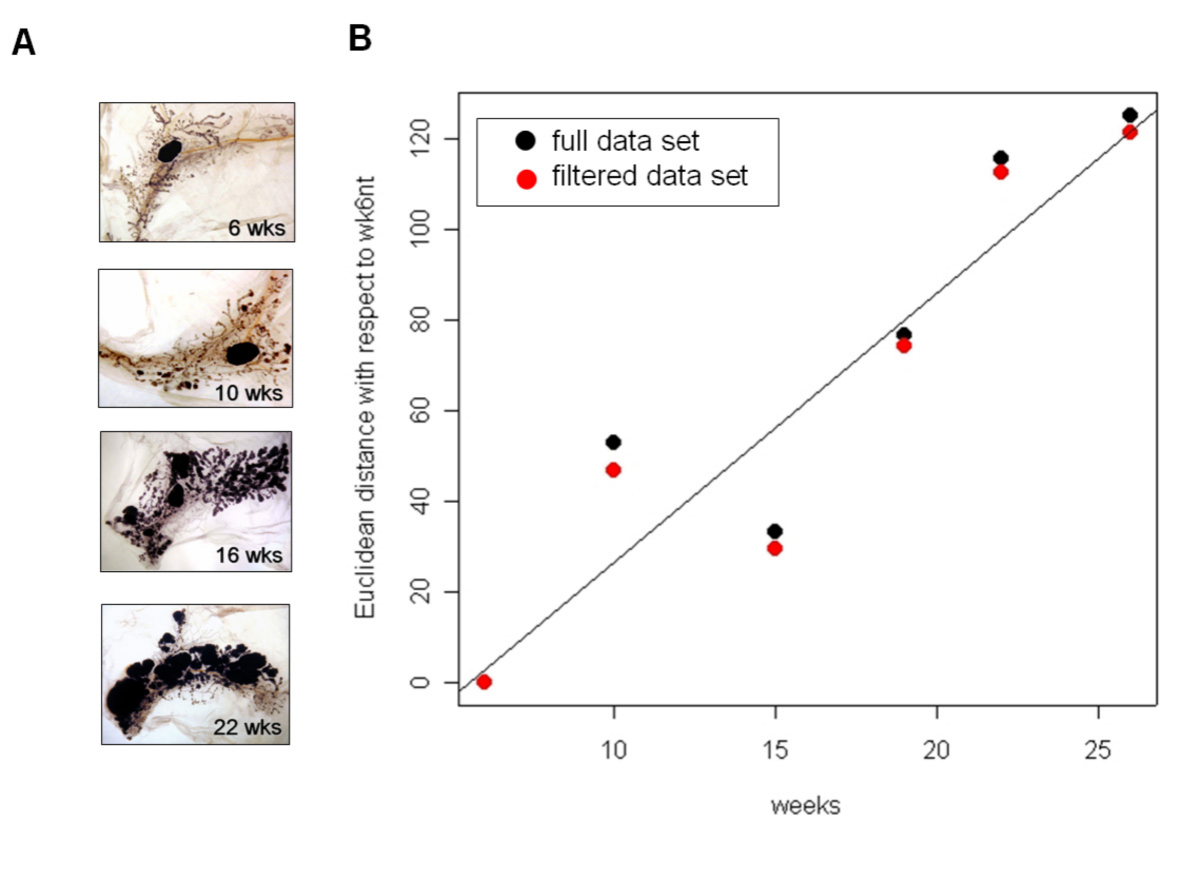
**BALB-neuT mammary gland morphology and transcription profile similarities**. A) Morphological preparation called "whole mount", that allows the observation of the entire mammary gland. The draining lymph node, vessels, ducts and ductules are evident. Clearly the amount of tumor mass increases from 6 to 22 weeks. 19 weeks picture was omitted since it is very similar to 22 weeks. At 26 weeks the tumor mass is palpable in all the mammary glands, and the "whole mount" picture is very similar to 22 weeks old mice (not shown). B) Euclidean distance of the expression profiles derived by mammary glands from 6 to 26 weeks old BALB-neuT mice measured with respect to 6 weeks old mice. Black dots refer to the average distance generated using all microarray data. Red dots refer to the average distance calculated using only microarray data showing an IQR greater than 0.5.

**Figure 2:**
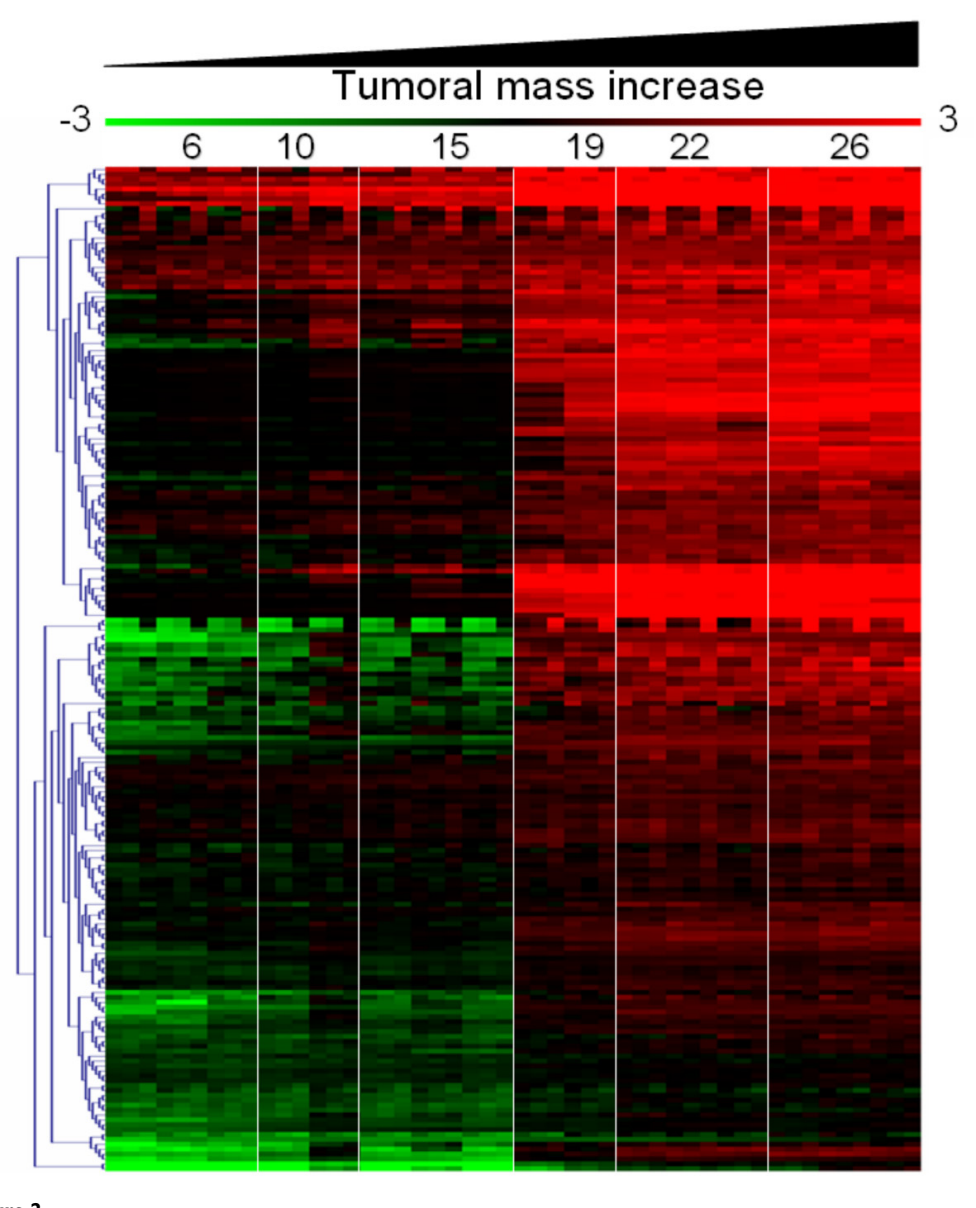
**Transcription profiles as virtual two dye experiments**. Each of the experimental points are represented as ratio with respect to wk2prg mice displaying marked pregnancy-related hyperplasia. The expression level of the genes associated to tumor cells increases with the animal age since tumor cells are replacing the conventional cellular environment of a normal mammary gland.

**Figure 3:**
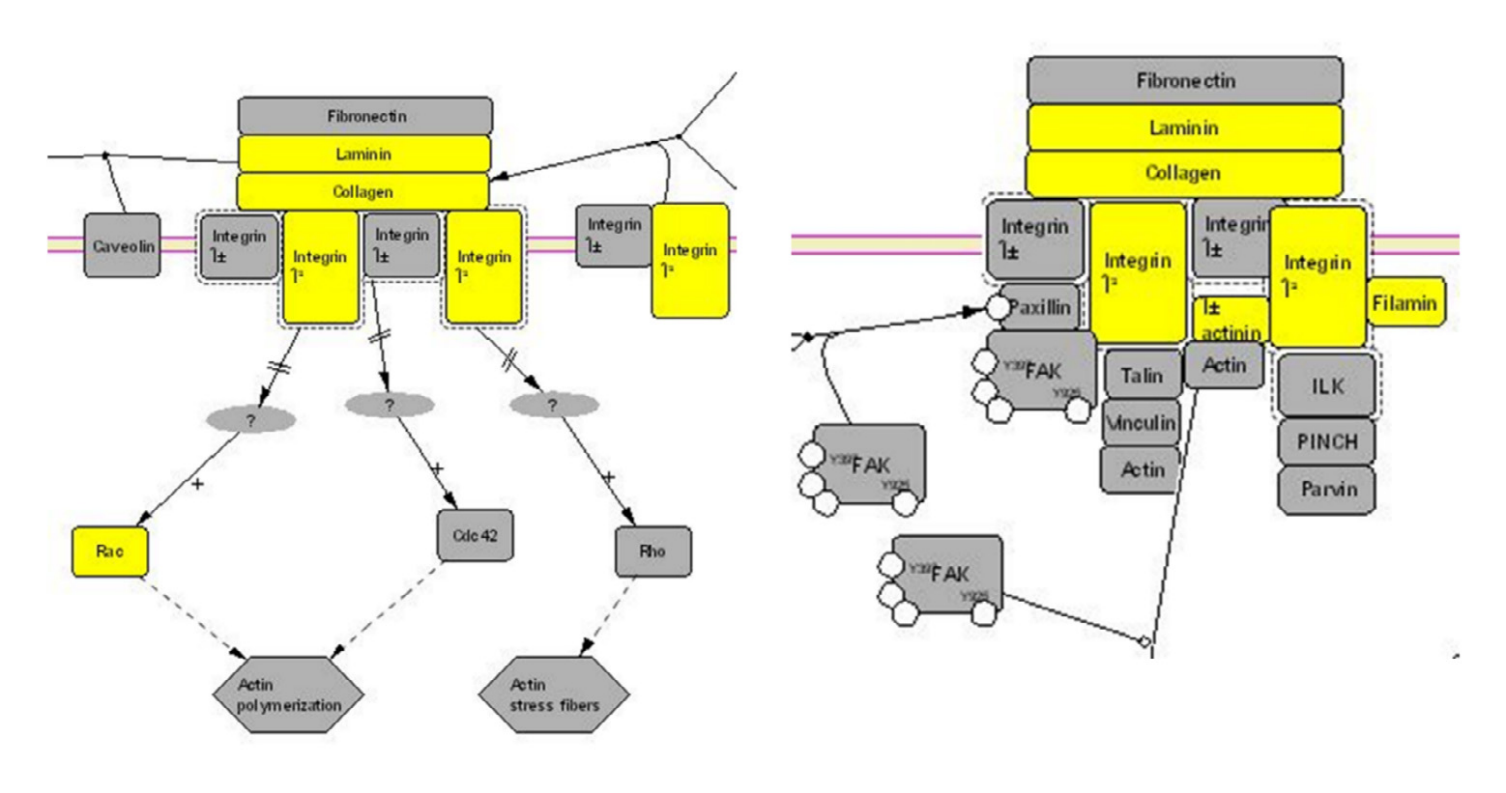
**Integrin signal transduction pathway**. Part of the integrin signal transduction pathway characterized by an over-representation of 6/204 genes showing an expression linearly linked to the tumor mass increment. The 6 genes found overrepresented are colored in yellow in the integrin pathway.

The number of neoplastic cells in the mammary tissue of BALB-neuT mice increases constantly during aging, and at 19–22 weeks a tumor mass becomes palpable in all their mammary glands. Previously [17] we have used PCA (Principal Component Analysis) to show that the transcriptional profiles of BALB-neuT mammary glands are linearly correlated with age (i.e. in function of the increment of tumor cellularity in the mammary glands, fig. 1). We identified 205 probe sets by linear fitting each gene expression with respect to age (fig. 2). Only one probe set was linked to an unmapped EST , whereas the others were linked to annotated mouse genes. Investigation of specific gene ontology (GO) class (BP, biological process; MF, molecular function; CC, cellular component) over-representation within the annotated 204 genes revealed significant enrichments in functions associated with the extracellular compartment (table 1). Pathway analysis showed the presence of 6 genes involved in the integrin signal transduction pathway (fig. 3) linked to Her2 action [20,21].

### Evaluation of the expression of tumor-related genes in normal mouse tissues

If a TAA is to be used as a vaccination target, its expression must be low in normal tissues. To evaluate the expression of the 204 putative TAAs in normal tissues, we used transcription profiles recently published by Su [22]. The mouse atlas (GNFm) was generated by using a custom Affymetrix array which interrogates 17,108 annotated mouse genes: 171 of the putative TAAs are present in GNFm, twelve of them (table 2) characterized by low expression (see material and methods) in normal mouse tissues.

### Expression of tumor related genes in normal and cancer human tissues

The 12 genes identified as TAAs were expressed in all the stages of breast carcinogenesis in BALB-neuT mice and characterized by low expression in normal mouse tissues. To determine whether these characteristics were also present in the corresponding human genes, we cross-validated the expression of the 12 TAAs within the human tissue expression atlas published by Yanai [23]. Since Her2 is expressed at very low levels in normal human tissues, we used it as the "ideal reference" of low expression. Rtn1, Irf6, Sel1L were discarded since their expression levels in some human tissues were higher than those of Her2 (data not shown). Tes, Rcn2, Rnf4, Cradd, Galnt3, Clca1, Cdcp1, Socs2 and Spred2 were expressed at levels similar to Her2.

We also assessed the expression of these 9 TAAs in human breast specimens. Of the breast cancer transcriptional profiling data sets available [24-26], that proposed by van't Veer [26] is ideal because its experimental structure allows evaluation of the expression level distribution within the specimens. Fold change variation of each specimen is measured with respect to a reference pool composed by mixing the same amount of all RNA of the sporadic patients [26].

**Figure 4:**
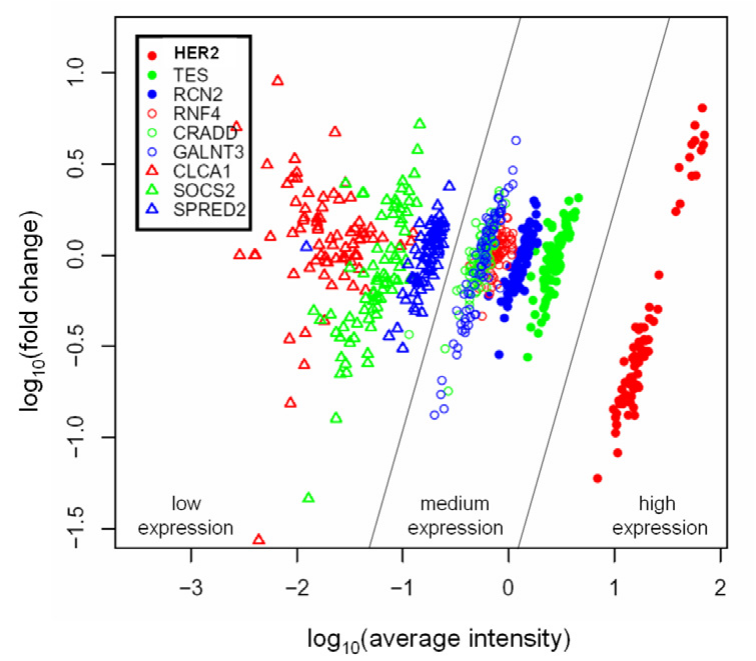
**Fold change variation versus average intensities of putative TAAs in van't Veer data set**. Log_10 _fold change variation with respect to a reference pool (the reference pool is made of the same amount of all sporadic patients [26]) is plotted in function of log_10 _average intensities [42]. The 9 putative TAAs are grouped with respect to Her2 expression as medium expressed and low expressed. Genes belonging to the medium expressed group have a median average expression level between 1/10 to 1/100 of median Her2 expression. Genes belonging to the low expressed group have a median average expression level lower than 1/100 of median Her2 expression.

The 9 TAAs and Her2 were represented in the van't Veer set [26], Her2 was highly expressed, while Tes, Rcn2, Rnf4, Cradd and Galnt3 were medium expressed. Clca1, Cdcp1, Socs2 and Spred2 were low expressed group (fig. 4) and since their expression was 100 times lower than that of Her2 they were discarded as putative vaccination targets. Even though Tes, Rcn2, Rnf4, Cradd and Galnt3 were less expressed than Her2, they were less scattered than Her2 (fig. 4). The highest expressed is Tes, which maps to a fragile site on chromosome 7q31.2 [27] and may have a tumor suppression activity [28]. It is homogeneously expressed in all the 78 cancer specimens in the van't Veer set [26]. The function of Rcn2, reticulocalbin, has not been characterized. It is implicated in tumor cell invasiveness, as it is expressed in the highly invasive breast cancer cell lines, but not in poorly invasive ones [29]. Rnf4 is a RING-finger protein which acts as a transcription regulator [30], it is not only a coactivator in steroid receptor-dependent transcription but also activates transcription from steroid-independent promoters [31]. It is expressed at very high levels in testis and at much lower levels in several other tissues. When ectopically expressed, it inhibits the proliferation of both somatic and germ cell tumor-derived cells [32,33]. Cradd encodes a death domain (CARD/DD)-containing protein and induces apoptosis [34,35]. Galnt3 encodes UDP-GalNAc transferase 3, a member of the GalNAc-transferases family. In gallbladder cancer, the presence of diffuse-type localization of GalNAc-T3 in the subserosal layer is correlated with aggressiveness [36]. Furthermore, Galnt3 expression is associated with the differentiation and aggressiveness of ductal adenocarcinoma of the pancreas [37].

## Conclusion

This study identified 12 putative TAAs. Three were discarded because they were expressed in some normal human tissues and four because their expression was too low in human cancer specimens. Tes, Rcn2, Rnf4, Cradd and Galnt3 were retained for further studies. This work was based on in silico analysis of published transcriptional profiling studies. Its results must now be referred back to the in vivo pre-clinical experimentation.

We shall select the most immunogenic domains of the 5 candidate TAAs and use them in vaccination protocols in BALB-neuT mice [9,38]. Other interesting TAAs may have been missed in this analysis since it integrates two independent experiments and this increases the experimental noise. A further limitation could be the partial coverage of the mouse transcriptome of the arrays employed. A new transcription profiling study covering the same experimental points, but using full genome-wide mouse arrays is now in progress.

## Methods

### Microarray data

The .CEL files from Quaglino [1], Astolfi [2], Su [22] were kindly provided by the authors. The van't Veer data set [26] was obtained from the Rosetta Inpharmatics web site .

### Data analysis

Microarray data analysis was performed using Bioconductor libraries [39]. Probe set intensities were calculated using the gcRMA algorithm  and normalized by the quantiles method [40]. The full data set (wk6nt 4 arrays, wk10nt 2 arrays, wk15t 3 arrays, wk19nt 2 arrays, wk22nt 3 arrays, wk26nt 3 arrays) was filtered to select probe sets with an intra-experiment Inter Quantile Range (IQR) greater than 0.5. We used a statistical linear model to measure the effects of time on gene expression and to identify probe sets linearly correlated to the increment of the tumor mass. In equation (1) y_*ij *_is the observed expression level for probe set *i *in sample *j *(*j *= 1,...,16). μ is the average expression level of probe set *i *and β_time _represents the effect of time on the expression level of probe set *i*, ε represents random error for probe set *i *and sample *j*, and is assumed to be independent for each probe set and sample, and normally distributed with mean 0 and variance σ^2^.

y_*ij *_= μ_*i *_+ β_time _+ ε_*ij *_    (1)

205 probe sets were identified as putative TAAs by selecting only the expression profiles characterized by an r^2 ^> 0.7, and a positive slope (p < 0.001) of the linear fitting of expression versus the animal age.

Virtual two-dye experiments were done as previously published [1] using as reference wk2prg (3 arrays).

GO class enrichment was evaluated with the Bioconductor GOstats package and the annotation library mgu74av2 1.6.8. Pathway analysis was performed with the PANTHER annotation tool .

Expression level of the probe sets within mouse tissues was evaluated using the mouse tissue expression atlas (GNFm) published by Su [22]. 171 of the 204 putative TAAs were mapped within GNFm. The expression of 12 of them was located in the low expression tail of the GNFm distribution (|log_2_(average intensity)|<7) and narrowly distributed within the GNFm tissues (IQR of the inter-tissue expression < 0.5). The expression of 12 TAAs was compared to that of Her2 in the human normal tissue expression atlas published by Yanai [23] by using their web interface .

The expression of 9 TAAs was evaluated within the breast cancer transcription profiling study produced by van't Veer [26]. Since in this experiment the gene identifiers are EST accession numbers, the Entrez Gene identifier [41] associated with each putative TAA was searched in the homologene database  and the corresponding orthologous human Entrez Gene identifier (HsEG) was selected. A Perl  script was used to link HsEG to all the accession numbers of EST associated with them in the UniGene database (downloaded from  on 3/3/2005). The ESTs accession numbers were then searched in the van't Veer data set [26], together with the Her2 gene. In this experiment, RNA expression for each of the 78 breast cancer tumors is evaluated as the log_10 _differential expression with respect to a pool composed by mixing the same amount of all RNA of the sporadic patients [26].

## Authors' contributions

CaF and AA carried out the microarray experiments and drafted the manuscript. MI performed whole mount analysis of BALB-neuT mammary glands. CoF participated in the microarray data analysis. CaF, LPL and FG participated in the design of the study and performed literature analysis. CR conceived the study, participated in its design and coordination and helped to draft the manuscript. All authors read and approved the final manuscript.

## Supplementary Material

Additional File 1 **Annotation of the 204 genes showing an expression linearly related to the tumor mass increase. **e annotation of the 204 genes showing a linear correlation with the increase of the tumor mass in the BALB-neuT murine cancer model was carried out using the mgu74v2 1.6.8 and the annaffy Bioconductor libraries. Click here for file

## Figures and Tables

**Table 1:** GO classes found enriched by mean of hypergeometric distribution within the 204 genes linearly linked to tumor mass increase.

**GO class**	**Description**	**p value**	**N. of found items within the 204 differentially expressed genes**	**N. of items belonging to the GO class within the mgu74av2 chip.**	**GO**
GO:0007155	Cell adhesion	0.000	19	250	BP
GO:0030198	Extracellular matrix organization and biogenesis	0.005	3	17	BP
GO:0007160	Cell-matrix adhesion	0.005	4	34	BP
GO:0005201	Extracellular matrix structural constituent	0.003	5	47	MF
GO:0008201	Heparin binding	0.004	4	39	MF
GO:0005576	Extracellular	0.000	58	1631	CC
GO:0005615	Extracellular space	0.000	54	1514	CC
GO:0005794	Golgi apparatus	0.004	12	209	CC
GO:0005581	collagen	0.004	4	28	CC
GO:0016327	Apicolateral plasma membrane	0.004	6	65	CC
GO:0016020	Membrane	0.005	69	22285	CC
GO:0030054	Cell junction	0.006	6	71	CC
GO:0005923	Tight junction	0.007	3	17	CC
GO:0005911	Intercellular junction	0.007	5	52	CC
GO:0030057	Desmosome	0.008	2	6	CC
GO:0005578	Extracellular matrix	0.008	11	204	CC

**Table 2:** Genes, having expression linked to tumor mass increase, showing low expression levels in the GNFm tissue atlas. 12 genes were showing a limited expression at transcriptional level in the normal murine tissue expression atlas.

**Symbol**	**GNFm ID**	**Mm Affy IDs**	**Mm Gene IDs**	**Hs Gene IDs**	**Expression similar to Her2 in human tissues**
Socs2	gnf1m24576_at	99475_at	216233	8835	Y
Spred2	gnf1m09332_s_at	161070_at	114716	200734	Y
Cdcp1	gnf1m12020_a_at	104198_at	109332	64866	Y
Rtn1	gnf1m15854_at	94545_at	104001	6252	N
Irf6	gnf1m13081_at	92440_at	54139	3664	N
Rcn2	gnf1m24916_at	93281_at	26611	5955	Y
Tes	gnf1m02859_at	101551_s_at	21753	26136	Y
Sel1L	gnf1m30768_a_at	92870_at, 92871_at	20338	6400	N
Rnf4	gnf1m25286_at	93782_at	19822	6047	Y
Galnt3	gnf1m24047_at	99011_at	14425	2591	Y
Cradd	gnf1m27661_at	102952_g_at	12905	8738	Y
Clca1	gnf1m13262_a_at	103812_at	12722	1179	Y
