# Supplementary material for: An integrated approach of immunogenomics and bioinformatics to identify new Tumor Associated Antigens (TAA) for mammary cancer immunological prevention
Source: BMC Bioinformatics. 2005 Dec 1;6(Suppl 4):S7. doi: 10.1186/1471-2105-6-S4-S7 (PMC1866378; doi:10.1186/1471-2105-6-S4-S7)
Supplement: Additional File 1 — Annotation of the 204 genes showing an expression linearly related to the tumor mass increase. e annotation of the 204 genes showing a linear correlation with the increase of the tumor mass in the BALB-neuT murine cancer model was carried out using the mgu74v2 1.6.8 and the annaffy Bioconductor libraries. [file 1471-2105-6-S4-S7-S1.html]

Bioconductor Affymetrix Probe Listing


# Bioconductor Affymetrix Probe Listing

| Probe | Symbol | Description | LocusLink | Cytoband | PubMed | Gene Ontology | Pathway | wk6nt.2.CEL | wk6nt.3.CEL | wk6nt.4.CEL | wk10nt.5.CEL | wk10nt.6.CEL | wk15nt.5.CEL | wk15nt.6.CEL | wk15nt.7.CEL | wk19nt.14.CEL | wk19nt.15.CEL | wk22nt.15.CEL | wk22nt.7.CEL | wk22nt.8.CEL | wk26nt.16.CEL | wk26nt.17.CEL | wk26nt.18.CEL | wk2pgn.13.CEL | wk2pgn.2.CEL | wk2pgn.3.CEL | wk6.1.17 | wk6.1.18 | wk6.1.19 | wk6.2.17 | wk6.2.18 | wk6.2.19 | wk6.3.17 | wk6.3.18 | wk6.3.19 | wk10.4.17 | wk10.4.18 | wk10.4.19 | wk10.5.17 | wk10.5.18 | wk10.5.19 | wk15.6.17 | wk15.6.18 | wk15.6.19 | wk15.7.17 | wk15.7.18 | wk15.7.19 | wk15.8.17 | wk15.8.18 | wk15.8.19 | wk19.9.17 | wk19.9.18 | wk19.9.19 | wk19.10.17 | wk19.10.18 | wk19.10.19 | wk22.11.17 | wk22.11.18 | wk22.11.19 | wk22.12.17 | wk22.12.18 | wk22.12.19 | wk22.13.17 | wk22.13.18 | wk22.13.19 | wk26.14.17 | wk26.14.18 | wk26.14.19 | wk26.15.17 | wk26.15.18 | wk26.15.19 | wk26.16.17 | wk26.16.18 | wk26.16.19 |
| --- | --- | --- | --- | --- | --- | --- | --- | --- | --- | --- | --- | --- | --- | --- | --- | --- | --- | --- | --- | --- | --- | --- | --- | --- | --- | --- | --- | --- | --- | --- | --- | --- | --- | --- | --- | --- | --- | --- | --- | --- | --- | --- | --- | --- | --- | --- | --- | --- | --- | --- | --- | --- | --- | --- | --- | --- | --- | --- | --- | --- | --- | --- | --- | --- | --- | --- | --- | --- | --- | --- | --- | --- | --- | --- |
| 100084\_at | Vil2 | villin 2 | 22350 | 17 | 14 | apical part of cell  apical plasma membrane  basal body  cellular morphogenesis  cytoplasm  cytoskeletal protein binding  cytoskeleton  membrane  protein binding  structural molecule activity |  | 6.48932 | 7.3567 | 6.88066 | 7.1281 | 7.27796 | 6.88957 | 7.05291 | 6.7025 | 8.59907 | 8.42539 | 9.58526 | 9.19485 | 8.65504 | 9.24763 | 9.98642 | 9.74563 | 6.8959 | 7.15869 | 7.16723 | -0.406581 | -0.669368 | -0.677912 | 0.4608 | 0.198012 | 0.189468 | -0.0152408 | -0.278028 | -0.286572 | 0.232199 | -0.0305882 | -0.0391323 | 0.382056 | 0.119269 | 0.110725 | -0.00632595 | -0.269113 | -0.277657 | 0.157013 | -0.105775 | -0.114319 | -0.193402 | -0.45619 | -0.464734 | 1.70317 | 1.44038 | 1.43184 | 1.52949 | 1.2667 | 1.25816 | 2.68935 | 2.42657 | 2.41802 | 2.29895 | 2.03616 | 2.02762 | 1.75914 | 1.49635 | 1.48781 | 2.35173 | 2.08894 | 2.08039 | 3.09052 | 2.82773 | 2.81919 | 2.84973 | 2.58694 | 2.5784 |
| 100128\_at | Cdc2a | cell division cycle 2 homolog A (S. pombe) | 12534 | 10 | 14 | ATP binding  Hsp70 protein binding  cell cycle  kinase activity  mitosis  mitotic G2 checkpoint  nucleus  protein amino acid phosphorylation  protein binding  protein kinase activity  protein serine/threonine kinase activity  protein-tyrosine kinase activity  transferase activity |  | 2.51562 | 2.69635 | 3.33459 | 3.1608 | 2.99124 | 2.64503 | 2.85617 | 2.68803 | 5.59571 | 6.54178 | 7.21612 | 7.2527 | 7.08983 | 7.09177 | 7.78141 | 6.02867 | 4.14636 | 4.12858 | 4.0894 | -1.63073 | -1.61296 | -1.57377 | -1.45001 | -1.43224 | -1.39305 | -0.811766 | -0.793991 | -0.754806 | -0.985554 | -0.967779 | -0.928594 | -1.15512 | -1.13734 | -1.09816 | -1.50133 | -1.48356 | -1.44437 | -1.29019 | -1.27241 | -1.23323 | -1.45833 | -1.44056 | -1.40137 | 1.44935 | 1.46713 | 1.50631 | 2.39542 | 2.4132 | 2.45238 | 3.06977 | 3.08754 | 3.12673 | 3.10634 | 3.12412 | 3.1633 | 2.94347 | 2.96125 | 3.00043 | 2.94541 | 2.96318 | 3.00237 | 3.63505 | 3.65283 | 3.69201 | 1.88232 | 1.90009 | 1.93928 |
| 100155\_at | Ddr1 | discoidin domain receptor family, member 1 | 12305 | 17 | 11 | ATP binding  cell adhesion  extracellular space  integral to membrane  kinase activity  membrane  protein amino acid phosphorylation  protein kinase activity  protein serine/threonine kinase activity  protein-tyrosine kinase activity  receptor activity  transferase activity  transmembrane receptor protein tyrosine kinase activity  transmembrane receptor protein tyrosine kinase signaling pathway |  | 2.75406 | 2.82903 | 2.89897 | 2.79855 | 2.85968 | 2.84268 | 2.82572 | 2.86001 | 4.00522 | 5.79642 | 7.47642 | 8.17007 | 7.33285 | 7.67615 | 8.90692 | 7.99653 | 2.81331 | 2.84087 | 2.91749 | -0.0592551 | -0.0868167 | -0.16343 | 0.0157217 | -0.0118399 | -0.0884535 | 0.0856616 | 0.0581 | -0.0185136 | -0.0147596 | -0.0423212 | -0.118935 | 0.0463679 | 0.0188063 | -0.0578073 | 0.0293636 | 0.00180198 | -0.0748117 | 0.0124091 | -0.0151525 | -0.0917661 | 0.0467014 | 0.0191398 | -0.0574738 | 1.19191 | 1.16435 | 1.08773 | 2.98311 | 2.95555 | 2.87894 | 4.66311 | 4.63555 | 4.55893 | 5.35676 | 5.3292 | 5.25258 | 4.51953 | 4.49197 | 4.41536 | 4.86284 | 4.83528 | 4.75867 | 6.09361 | 6.06604 | 5.98943 | 5.18321 | 5.15565 | 5.07904 |
| 100283\_at | Sox10 | SRY-box containing gene 10 | 20665 | 15 | 13 | DNA binding  cell differentiation  nucleus  regulation of transcription, DNA-dependent  transcription factor activity  transcription factor complex |  | 2.60138 | 2.67961 | 3.66026 | 4.58428 | 4.99757 | 3.99791 | 5.03715 | 3.23522 | 8.15246 | 5.88253 | 7.99836 | 7.69209 | 8.27979 | 9.57213 | 9.38593 | 9.41399 | 6.02689 | 4.97889 | 4.92376 | -3.42551 | -2.37751 | -2.32237 | -3.34729 | -2.29928 | -2.24415 | -2.36663 | -1.31863 | -1.2635 | -1.44261 | -0.394611 | -0.339477 | -1.02933 | 0.0186763 | 0.0738105 | -2.02898 | -0.980978 | -0.925843 | -0.989749 | 0.058255 | 0.113389 | -2.79168 | -1.74367 | -1.68854 | 2.12557 | 3.17357 | 3.22871 | -0.144362 | 0.903642 | 0.958776 | 1.97146 | 3.01947 | 3.0746 | 1.66519 | 2.71319 | 2.76833 | 2.25289 | 3.3009 | 3.35603 | 3.54523 | 4.59324 | 4.64837 | 3.35904 | 4.40704 | 4.46218 | 3.3871 | 4.4351 | 4.49023 |
| 100428\_at | Lamc2 | laminin, gamma 2 | 16782 | 1 | 6 | basal lamina  basement membrane  cell adhesion  electron transport  extracellular matrix  extracellular matrix structural constituent  extracellular space  heparin binding  membrane  protein binding  structural molecule activity  ubiquinol-cytochrome-c reductase complex |  | 1.56576 | 2.37152 | 2.41222 | 3.44256 | 6.28735 | 2.13215 | 4.45494 | 2.0604 | 6.88062 | 7.8347 | 9.14864 | 8.87076 | 8.81198 | 8.9365 | 8.30954 | 9.27583 | 4.69853 | 5.41439 | 5.71272 | -3.13277 | -3.84863 | -4.14696 | -2.327 | -3.04286 | -3.3412 | -2.28631 | -3.00217 | -3.3005 | -1.25597 | -1.97183 | -2.27016 | 1.58882 | 0.872959 | 0.574624 | -2.56638 | -3.28224 | -3.58058 | -0.243585 | -0.959442 | -1.25778 | -2.63813 | -3.35399 | -3.65232 | 2.18209 | 1.46624 | 1.1679 | 3.13617 | 2.42031 | 2.12198 | 4.45011 | 3.73425 | 3.43592 | 4.17223 | 3.45637 | 3.15804 | 4.11345 | 3.3976 | 3.09926 | 4.23797 | 3.52212 | 3.22378 | 3.61101 | 2.89516 | 2.59682 | 4.5773 | 3.86145 | 3.56311 |
| 100481\_at | Col11a1 | procollagen, type XI, alpha 1 | 12814 | 3 | 11 | cartilage condensation  cell adhesion  collagen  collagen fibril organization  cytoplasm  extracellular matrix  extracellular matrix structural constituent  extracellular matrix structural constituent conferring tensile strength  phosphate transport  proteoglycan metabolism  structural molecule activity |  | 3.98951 | 4.03859 | 4.06757 | 3.97956 | 4.36746 | 4.07585 | 4.91878 | 4.08286 | 10.1089 | 10.3477 | 10.9292 | 10.5383 | 10.0846 | 11.1041 | 11.1133 | 11.0758 | 3.95213 | 3.95816 | 3.9236 | 0.0373837 | 0.0313491 | 0.0659134 | 0.0864575 | 0.0804228 | 0.114987 | 0.115442 | 0.109407 | 0.143972 | 0.0274271 | 0.0213924 | 0.0559568 | 0.415333 | 0.409298 | 0.443863 | 0.123725 | 0.117691 | 0.152255 | 0.966657 | 0.960622 | 0.995186 | 0.13073 | 0.124695 | 0.159259 | 6.15673 | 6.1507 | 6.18526 | 6.3956 | 6.38956 | 6.42413 | 6.97709 | 6.97105 | 7.00562 | 6.58616 | 6.58013 | 6.61469 | 6.13246 | 6.12642 | 6.16099 | 7.15201 | 7.14598 | 7.18054 | 7.16113 | 7.1551 | 7.18966 | 7.12363 | 7.1176 | 7.15216 |
| 100522\_s\_at | Wbp5 | WW domain binding protein 5 | 22381 | X | 7 |  |  | 9.78811 | 10.3759 | 10.6817 | 10.1959 | 11.2581 | 10.2826 | 10.4655 | 9.95978 | 11.4226 | 11.631 | 12.2275 | 12.4494 | 11.9782 | 12.299 | 12.6025 | 12.3835 | 11.7431 | 11.8975 | 11.4588 | -1.95497 | -2.10936 | -1.67069 | -1.36718 | -1.52157 | -1.0829 | -1.06135 | -1.21574 | -0.777067 | -1.5472 | -1.70159 | -1.26292 | -0.48494 | -0.639334 | -0.20066 | -1.4605 | -1.6149 | -1.17622 | -1.27762 | -1.43202 | -0.993345 | -1.7833 | -1.9377 | -1.49902 | -0.320495 | -0.474889 | -0.0362154 | -0.112054 | -0.266449 | 0.172225 | 0.484374 | 0.32998 | 0.768654 | 0.706306 | 0.551912 | 0.990586 | 0.235102 | 0.0807079 | 0.519382 | 0.555889 | 0.401495 | 0.840169 | 0.859469 | 0.705074 | 1.14375 | 0.640377 | 0.485982 | 0.924656 |
| 100581\_at | Cstb | cystatin B | 13014 | 10 | 7 | cysteine protease inhibitor activity  endopeptidase inhibitor activity  intracellular |  | 9.7429 | 9.45038 | 9.12017 | 9.60424 | 9.79608 | 9.35394 | 9.88831 | 9.44982 | 10.2195 | 10.3334 | 10.4843 | 10.5127 | 10.5855 | 10.3851 | 11.1962 | 10.6188 | 10.4379 | 10.5607 | 10.1185 | -0.695047 | -0.817822 | -0.375622 | -0.987562 | -1.11034 | -0.668137 | -1.31777 | -1.44055 | -0.998349 | -0.833701 | -0.956476 | -0.514276 | -0.641866 | -0.764642 | -0.322442 | -1.08401 | -1.20678 | -0.764581 | -0.549631 | -0.672407 | -0.230207 | -0.988124 | -1.1109 | -0.668699 | -0.218481 | -0.341256 | 0.100944 | -0.10456 | -0.227335 | 0.214865 | 0.0463271 | -0.0764483 | 0.365752 | 0.0747722 | -0.0480031 | 0.394197 | 0.147593 | 0.0248177 | 0.467018 | -0.0528601 | -0.175635 | 0.266564 | 0.758215 | 0.63544 | 1.07764 | 0.180865 | 0.0580897 | 0.50029 |
| 100601\_at | Itgb5 | integrin beta 5 | 16419 | 16 | 11 | cell adhesion  cell-matrix adhesion  development  extracellular space  integral to membrane  integrin complex  integrin-mediated signaling pathway  protein binding  receptor activity |  | 3.50547 | 4.20174 | 4.34407 | 4.26578 | 5.2335 | 4.47108 | 4.05341 | 4.1396 | 5.74127 | 5.94074 | 6.21138 | 7.15536 | 6.52316 | 6.64287 | 6.57706 | 7.46285 | 3.98862 | 4.71791 | 4.706 | -0.48315 | -1.21244 | -1.20053 | 0.213116 | -0.516177 | -0.50426 | 0.355453 | -0.37384 | -0.361923 | 0.277164 | -0.452129 | -0.440211 | 1.24488 | 0.51559 | 0.527507 | 0.482456 | -0.246837 | -0.23492 | 0.064793 | -0.6645 | -0.652583 | 0.15098 | -0.578313 | -0.566395 | 1.75265 | 1.02336 | 1.03528 | 1.95212 | 1.22283 | 1.23474 | 2.22276 | 1.49346 | 1.50538 | 3.16674 | 2.43745 | 2.44937 | 2.53454 | 1.80525 | 1.81717 | 2.65425 | 1.92496 | 1.93687 | 2.58844 | 1.85915 | 1.87107 | 3.47423 | 2.74493 | 2.75685 |
| 101069\_g\_at | Mkrn1 | makorin, ring finger protein, 1 | 54484 | 6 | 8 | nucleic acid binding  protein ubiquitination  ubiquitin ligase complex  ubiquitin-protein ligase activity  zinc ion binding |  | 7.15664 | 7.34816 | 7.92013 | 6.96677 | 7.15747 | 7.52967 | 8.02894 | 7.3533 | 8.6989 | 9.22803 | 8.80119 | 9.17715 | 8.66023 | 9.5044 | 10.1233 | 10.0585 | 7.78984 | 7.74556 | 6.83597 | -0.6332 | -0.588915 | 0.320669 | -0.441676 | -0.397391 | 0.512193 | 0.130294 | 0.174579 | 1.08416 | -0.823073 | -0.778788 | 0.130797 | -0.632374 | -0.58809 | 0.321495 | -0.260173 | -0.215888 | 0.693696 | 0.239098 | 0.283383 | 1.19297 | -0.436537 | -0.392252 | 0.517332 | 0.909064 | 0.953349 | 1.86293 | 1.43819 | 1.48247 | 2.39206 | 1.01135 | 1.05563 | 1.96521 | 1.38731 | 1.43159 | 2.34118 | 0.870386 | 0.91467 | 1.82425 | 1.71456 | 1.75884 | 2.66843 | 2.33343 | 2.37771 | 3.2873 | 2.26867 | 2.31296 | 3.22254 |
| 101070\_at | Mkrn1 | makorin, ring finger protein, 1 | 54484 | 6 | 8 | nucleic acid binding  protein ubiquitination  ubiquitin ligase complex  ubiquitin-protein ligase activity  zinc ion binding |  | 5.5176 | 5.39477 | 6.04718 | 5.6826 | 5.98337 | 5.8244 | 5.98385 | 5.91799 | 6.31252 | 6.36648 | 6.2097 | 6.81008 | 6.62389 | 7.52981 | 7.14484 | 7.62006 | 5.69251 | 5.3091 | 5.58772 | -0.174918 | 0.208493 | -0.070127 | -0.29774 | 0.0856707 | -0.192949 | 0.354663 | 0.738074 | 0.459454 | -0.00991213 | 0.373499 | 0.0948789 | 0.29086 | 0.674271 | 0.395651 | 0.131885 | 0.515296 | 0.236676 | 0.291338 | 0.674749 | 0.396129 | 0.225473 | 0.608884 | 0.330264 | 0.620005 | 1.00342 | 0.724796 | 0.673965 | 1.05738 | 0.778756 | 0.517189 | 0.9006 | 0.62198 | 1.11756 | 1.50098 | 1.22236 | 0.931379 | 1.31479 | 1.03617 | 1.8373 | 2.22071 | 1.94209 | 1.45233 | 1.83574 | 1.55712 | 1.92754 | 2.31095 | 2.03233 |
| 101446\_at | Tpd52l1 | tumor protein D52-like 1 | 21987 | 10 | 4 | early endosome  protein binding |  | 3.12383 | 3.73304 | 4.03483 | 3.93207 | 7.18351 | 3.88753 | 5.74596 | 3.91796 | 8.4155 | 9.02132 | 9.98033 | 10.1829 | 9.90761 | 9.57104 | 10.3172 | 9.58793 | 5.13936 | 5.52282 | 4.13127 | -2.01553 | -2.39899 | -1.00744 | -1.40632 | -1.78978 | -0.398225 | -1.10453 | -1.48799 | -0.0964411 | -1.20729 | -1.59075 | -0.199194 | 2.04415 | 1.66069 | 3.05224 | -1.25183 | -1.63529 | -0.24374 | 0.606607 | 0.223145 | 1.6147 | -1.22139 | -1.60486 | -0.213303 | 3.27614 | 2.89268 | 4.28423 | 3.88197 | 3.4985 | 4.89006 | 4.84097 | 4.45751 | 5.84906 | 5.04354 | 4.66008 | 6.05164 | 4.76825 | 4.38479 | 5.77634 | 4.43169 | 4.04822 | 5.43978 | 5.17789 | 4.79443 | 6.18598 | 4.44857 | 4.06511 | 5.45666 |
| 101453\_at | Mia | melanoma inhibitory activity | 12587 | 7 | 5 | cell-matrix adhesion  extracellular  extracellular matrix organization and biogenesis  extracellular space  growth factor activity |  | 2.10196 | 2.17509 | 2.28138 | 2.09213 | 4.15148 | 2.22897 | 2.82673 | 2.31997 | 8.41793 | 9.62216 | 9.96443 | 10.367 | 10.7223 | 10.3152 | 11.2267 | 11.1012 | 2.17468 | 2.1832 | 2.70776 | -0.0727206 | -0.0812353 | -0.605794 | 0.000406375 | -0.0081083 | -0.532667 | 0.106696 | 0.0981808 | -0.426378 | -0.0825528 | -0.0910675 | -0.615626 | 1.9768 | 1.96828 | 1.44372 | 0.0542919 | 0.0457772 | -0.478781 | 0.652048 | 0.643534 | 0.118975 | 0.145283 | 0.136768 | -0.38779 | 6.24325 | 6.23473 | 5.71018 | 7.44748 | 7.43897 | 6.91441 | 7.78974 | 7.78123 | 7.25667 | 8.19234 | 8.18382 | 7.65926 | 8.54767 | 8.53915 | 8.01459 | 8.14056 | 8.13204 | 7.60748 | 9.05202 | 9.04351 | 8.51895 | 8.92654 | 8.91802 | 8.39346 |
| 101495\_at | Cd81 | CD 81 antigen | 12520 | 7 | 11 | integral to membrane  plasma membrane |  | 8.94281 | 9.17682 | 9.0889 | 10.0073 | 10.1373 | 9.12697 | 9.48035 | 9.07189 | 10.2571 | 10.5216 | 11.0735 | 11.0756 | 11.2846 | 10.7873 | 11.1546 | 11.4742 | 10.1244 | 10.1384 | 10.1113 | -1.18164 | -1.19556 | -1.16845 | -0.94762 | -0.961548 | -0.934434 | -1.03555 | -1.04947 | -1.02236 | -0.117155 | -0.131084 | -0.103969 | 0.0128522 | -0.00107643 | 0.026038 | -0.997474 | -1.0114 | -0.984288 | -0.644092 | -0.65802 | -0.630906 | -1.05255 | -1.06648 | -1.03937 | 0.132614 | 0.118686 | 0.1458 | 0.397186 | 0.383257 | 0.410372 | 0.949094 | 0.935165 | 0.96228 | 0.951144 | 0.937215 | 0.96433 | 1.16013 | 1.14621 | 1.17332 | 0.66282 | 0.648891 | 0.676006 | 1.03019 | 1.01626 | 1.04337 | 1.34976 | 1.33583 | 1.36295 |
| 101502\_at | Tgif | TG interacting factor | 21815 | 17 | 5 | DNA binding  nucleus  regulation of transcription, DNA-dependent  transcription factor activity  transcription factor complex |  | 3.92244 | 4.82217 | 6.03613 | 4.68605 | 6.84863 | 4.93507 | 6.73852 | 5.85788 | 9.37268 | 9.29072 | 9.16054 | 9.33555 | 8.7328 | 9.98035 | 9.48363 | 9.42517 | 4.23112 | 3.51736 | 4.68255 | -0.308684 | 0.405085 | -0.760108 | 0.591048 | 1.30482 | 0.139625 | 1.805 | 2.51877 | 1.35358 | 0.454921 | 1.16869 | 0.003498 | 2.6175 | 3.33127 | 2.16608 | 0.703942 | 1.41771 | 0.252519 | 2.5074 | 3.22117 | 2.05598 | 1.62676 | 2.34053 | 1.17533 | 5.14156 | 5.85533 | 4.69014 | 5.0596 | 5.77336 | 4.60817 | 4.92942 | 5.64319 | 4.47799 | 5.10442 | 5.81819 | 4.653 | 4.50168 | 5.21545 | 4.05025 | 5.74923 | 6.463 | 5.2978 | 5.2525 | 5.96627 | 4.80108 | 5.19405 | 5.90782 | 4.74262 |
| 101551\_s\_at | Tes | testis derived transcript | 21753 | 6 | 4 |  |  | 3.58389 | 3.71527 | 3.76139 | 3.44177 | 3.6891 | 3.49517 | 3.65631 | 3.72401 | 3.984 | 5.45942 | 7.63148 | 8.31579 | 7.298 | 8.0033 | 9.05752 | 8.42905 | 3.64796 | 3.8902 | 3.85823 | -0.0640761 | -0.306316 | -0.274344 | 0.0673077 | -0.174933 | -0.14296 | 0.113429 | -0.128812 | -0.0968394 | -0.206188 | -0.448428 | -0.416456 | 0.0411429 | -0.201097 | -0.169125 | -0.152793 | -0.395033 | -0.363061 | 0.00834399 | -0.233896 | -0.201924 | 0.0760518 | -0.166188 | -0.134216 | 0.336039 | 0.0937991 | 0.125771 | 1.81145 | 1.56921 | 1.60119 | 3.98351 | 3.74127 | 3.77325 | 4.66783 | 4.42559 | 4.45756 | 3.65004 | 3.4078 | 3.43977 | 4.35534 | 4.1131 | 4.14507 | 5.40956 | 5.16732 | 5.19929 | 4.78109 | 4.53885 | 4.57082 |
| 101560\_at | Emb | embigin | 13723 | 13 | 8 | integral to membrane |  | 2.32032 | 2.37925 | 2.41595 | 2.31094 | 2.31618 | 2.38169 | 2.35594 | 2.38548 | 6.31315 | 7.55322 | 9.00431 | 8.89068 | 8.7556 | 9.35764 | 9.28778 | 7.03417 | 2.27243 | 2.29118 | 2.25893 | 0.0478871 | 0.0291422 | 0.0613884 | 0.106819 | 0.0880741 | 0.12032 | 0.143518 | 0.124773 | 0.157019 | 0.038511 | 0.0197661 | 0.0520123 | 0.0437428 | 0.0249979 | 0.0572442 | 0.109257 | 0.0905119 | 0.122758 | 0.083508 | 0.0647631 | 0.0970094 | 0.113051 | 0.094306 | 0.126552 | 4.04072 | 4.02197 | 4.05422 | 5.28079 | 5.26204 | 5.29429 | 6.73188 | 6.71313 | 6.74538 | 6.61825 | 6.59951 | 6.63175 | 6.48317 | 6.46442 | 6.49667 | 7.0852 | 7.06646 | 7.0987 | 7.01535 | 6.9966 | 7.02885 | 4.76174 | 4.74299 | 4.77524 |
| 101637\_at | Ceacam10 | CEA-related cell adhesion molecule 10 | 26366 | 7 | 8 | extracellular space |  | 4.03161 | 4.10878 | 4.2055 | 4.02466 | 4.13483 | 4.17434 | 4.14062 | 4.15165 | 5.60571 | 8.02504 | 9.08312 | 9.58988 | 9.1146 | 9.47202 | 9.95078 | 10.1017 | 4.06833 | 4.10918 | 3.96592 | -0.0367196 | -0.0775675 | 0.0656874 | 0.0404482 | -0.000399686 | 0.142855 | 0.137175 | 0.0963267 | 0.239582 | -0.0436722 | -0.0845201 | 0.0587348 | 0.0664995 | 0.0256516 | 0.168906 | 0.106012 | 0.0651643 | 0.208419 | 0.07229 | 0.031442 | 0.174697 | 0.0833175 | 0.0424696 | 0.185724 | 1.53738 | 1.49654 | 1.63979 | 3.95671 | 3.91586 | 4.05912 | 5.01479 | 4.97394 | 5.11719 | 5.52155 | 5.4807 | 5.62395 | 5.04627 | 5.00542 | 5.14868 | 5.40369 | 5.36284 | 5.5061 | 5.88245 | 5.8416 | 5.98486 | 6.03338 | 5.99254 | 6.13579 |
| 101851\_at | Cd200 | Cd200 antigen | 17470 | 16 | 10 | extracellular space  integral to membrane |  | 5.23603 | 6.75255 | 6.45893 | 6.52195 | 6.77355 | 6.21911 | 6.60206 | 6.23096 | 7.10436 | 7.51075 | 8.17857 | 8.15804 | 7.94248 | 9.40529 | 8.97144 | 8.74713 | 4.60274 | 4.73088 | 4.92148 | 0.633296 | 0.505156 | 0.314549 | 2.14982 | 2.02168 | 1.83107 | 1.85619 | 1.72806 | 1.53745 | 1.91921 | 1.79107 | 1.60047 | 2.17082 | 2.04268 | 1.85207 | 1.61638 | 1.48824 | 1.29763 | 1.99933 | 1.87119 | 1.68058 | 1.62823 | 1.50009 | 1.30948 | 2.50163 | 2.37349 | 2.18288 | 2.90801 | 2.77988 | 2.58927 | 3.57583 | 3.44769 | 3.25708 | 3.5553 | 3.42716 | 3.23655 | 3.33974 | 3.2116 | 3.02099 | 4.80255 | 4.67441 | 4.48381 | 4.36871 | 4.24057 | 4.04996 | 4.14439 | 4.01626 | 3.82565 |
| 101955\_at | Hspa5 | heat shock 70kD protein 5 (glucose-regulated protein) | 14828 | 2 | 23 | ATP binding  response to ER-overload  endoplasmic reticulum  protein binding  protein folding  response to unfolded protein  ribosome binding | MAPK signaling pathway  Prion disease | 11.6728 | 11.8066 | 11.3598 | 11.4042 | 12.2811 | 11.8013 | 12.0027 | 11.7758 | 12.9141 | 12.8826 | 13.2897 | 13.1049 | 12.976 | 13.359 | 12.9358 | 13.0403 | 12.3014 | 12.7086 | 12.4891 | -0.628639 | -1.03588 | -0.816371 | -0.494835 | -0.902072 | -0.682567 | -0.941611 | -1.34885 | -1.12934 | -0.897172 | -1.30441 | -1.0849 | -0.0203207 | -0.427558 | -0.208053 | -0.500101 | -0.907338 | -0.687833 | -0.298689 | -0.705926 | -0.486421 | -0.525546 | -0.932783 | -0.713278 | 0.612733 | 0.205496 | 0.425002 | 0.581209 | 0.173972 | 0.393478 | 0.988259 | 0.581022 | 0.800527 | 0.803529 | 0.396292 | 0.615798 | 0.674605 | 0.267368 | 0.486873 | 1.05762 | 0.650381 | 0.869886 | 0.634416 | 0.227179 | 0.446684 | 0.738952 | 0.331715 | 0.55122 |
| 101973\_at | Cited2 | Cbp/p300-interacting transactivator, with Glu/Asp-rich carboxy-terminal domain, 2 | 17684 | 10 | 10 | central nervous system development  nucleus  regulation of transcription  regulation of transcription from Pol II promoter  regulation of transcription, DNA-dependent  transcription cofactor activity  transcription factor activity  transcription regulator activity |  | 9.14213 | 9.53172 | 10.1136 | 9.13463 | 10.0605 | 9.58715 | 9.78867 | 9.27329 | 10.1172 | 11.0069 | 11.211 | 11.2759 | 10.738 | 11.9451 | 11.7997 | 11.414 | 9.06629 | 8.73165 | 7.82089 | 0.0758388 | 0.410486 | 1.32124 | 0.465424 | 0.800071 | 1.71082 | 1.0473 | 1.38195 | 2.2927 | 0.0683401 | 0.402987 | 1.31374 | 0.994203 | 1.32885 | 2.2396 | 0.520853 | 0.8555 | 1.76625 | 0.72238 | 1.05703 | 1.96778 | 0.206998 | 0.541645 | 1.4524 | 1.05092 | 1.38557 | 2.29632 | 1.94061 | 2.27526 | 3.18601 | 2.14467 | 2.47931 | 3.39007 | 2.20957 | 2.54422 | 3.45497 | 1.67171 | 2.00636 | 2.91711 | 2.87876 | 3.21341 | 4.12416 | 2.73341 | 3.06806 | 3.97881 | 2.34771 | 2.68236 | 3.59311 |
| 101990\_at | Ldh2 | lactate dehydrogenase 2, B chain | 16832 | 6 | 7 | L-lactate dehydrogenase activity  glycolysis  oxidoreductase activity | Glycolysis / Gluconeogenesis  Cysteine metabolism  Pyruvate metabolism  Propanoate metabolism | 6.299 | 6.70145 | 6.5167 | 6.46867 | 7.11935 | 7.17262 | 7.22652 | 6.57578 | 8.22165 | 8.29886 | 8.34351 | 9.49199 | 9.17377 | 9.08546 | 10.0781 | 9.75774 | 6.28897 | 6.30876 | 6.08893 | 0.010038 | -0.00975882 | 0.210077 | 0.412485 | 0.392688 | 0.612524 | 0.227738 | 0.207942 | 0.427778 | 0.1797 | 0.159903 | 0.379739 | 0.830387 | 0.810591 | 1.03043 | 0.883658 | 0.863861 | 1.0837 | 0.937553 | 0.917756 | 1.13759 | 0.286814 | 0.267017 | 0.486853 | 1.93269 | 1.91289 | 2.13273 | 2.00989 | 1.99009 | 2.20993 | 2.05455 | 2.03475 | 2.25459 | 3.20302 | 3.18323 | 3.40306 | 2.8848 | 2.865 | 3.08484 | 2.7965 | 2.7767 | 2.99653 | 3.78913 | 3.76933 | 3.98917 | 3.46877 | 3.44898 | 3.66881 |
| 102001\_at | Rrm2 | ribonucleotide reductase M2 | 20135 | 12 | 14 | DNA replication  deoxyribonucleoside diphosphate metabolism  deoxyribonucleotide metabolism  oxidoreductase activity  protein binding  ribonucleoside-diphosphate reductase activity  ribonucleoside-diphosphate reductase complex | Purine metabolism  Pyrimidine metabolism | 3.92883 | 4.014 | 4.32993 | 3.93068 | 3.92732 | 4.11623 | 4.43866 | 4.1683 | 6.7208 | 6.59909 | 7.03809 | 6.99833 | 6.7539 | 7.23944 | 6.4547 | 6.23406 | 4.62503 | 4.58123 | 4.50077 | -0.696206 | -0.652402 | -0.571943 | -0.611028 | -0.567224 | -0.486765 | -0.2951 | -0.251296 | -0.170838 | -0.694354 | -0.65055 | -0.570091 | -0.697714 | -0.65391 | -0.573451 | -0.508805 | -0.465001 | -0.384542 | -0.186373 | -0.142569 | -0.0621103 | -0.45673 | -0.412926 | -0.332468 | 2.09577 | 2.13957 | 2.22003 | 1.97406 | 2.01786 | 2.09832 | 2.41306 | 2.45686 | 2.53732 | 2.37329 | 2.4171 | 2.49756 | 2.12887 | 2.17267 | 2.25313 | 2.6144 | 2.65821 | 2.73867 | 1.82967 | 1.87347 | 1.95393 | 1.60903 | 1.65283 | 1.73329 |
| 102012\_at | Scap2 | src family associated phosphoprotein 2 | 54353 | 6 | 6 | cytoplasm  negative regulation of cell proliferation |  | 7.86697 | 7.83508 | 7.87303 | 6.96894 | 7.78583 | 8.19377 | 8.19463 | 7.87315 | 9.18332 | 9.73812 | 9.52533 | 9.74066 | 9.63738 | 10.3 | 10.9426 | 10.6754 | 5.88258 | 5.79121 | 5.1827 | 1.98439 | 2.07576 | 2.68427 | 1.95249 | 2.04386 | 2.65238 | 1.99045 | 2.08182 | 2.69033 | 1.08635 | 1.17772 | 1.78624 | 1.90324 | 1.99462 | 2.60313 | 2.31118 | 2.40256 | 3.01107 | 2.31205 | 2.40342 | 3.01194 | 1.99056 | 2.08194 | 2.69045 | 3.30074 | 3.39211 | 4.00062 | 3.85554 | 3.94691 | 4.55543 | 3.64274 | 3.73411 | 4.34263 | 3.85808 | 3.94945 | 4.55797 | 3.75479 | 3.84617 | 4.45468 | 4.41746 | 4.50883 | 5.11734 | 5.05997 | 5.15134 | 5.75986 | 4.79286 | 4.88424 | 5.49275 |
| 102053\_at | Plscr2 | phospholipid scramblase 2 | 18828 | 9 | 7 | calcium ion binding  integral to membrane |  | 4.16206 | 4.28759 | 4.38553 | 4.1997 | 4.64265 | 4.32933 | 4.29351 | 4.36496 | 5.29179 | 7.07014 | 7.96924 | 7.74132 | 5.46703 | 7.65496 | 8.90442 | 7.93711 | 6.34178 | 6.14118 | 4.97464 | -2.17973 | -1.97913 | -0.812584 | -2.0542 | -1.8536 | -0.687053 | -1.95625 | -1.75565 | -0.58911 | -2.14208 | -1.94148 | -0.77494 | -1.69914 | -1.49853 | -0.331993 | -2.01246 | -1.81185 | -0.645312 | -2.04827 | -1.84767 | -0.681128 | -1.97682 | -1.77622 | -0.609681 | -1.05 | -0.849398 | 0.317144 | 0.728358 | 0.92896 | 2.0955 | 1.62746 | 1.82806 | 2.9946 | 1.39954 | 1.60014 | 2.76668 | -0.874753 | -0.674152 | 0.49239 | 1.31318 | 1.51378 | 2.68032 | 2.56264 | 2.76324 | 3.92978 | 1.59533 | 1.79593 | 2.96247 |
| 102064\_at | Casp1 | caspase 1 | 12362 | 9 | 17 | caspase activity  cysteine-type peptidase activity  cytoplasm  extracellular  hydrolase activity  induction of apoptosis  induction of apoptosis  induction of apoptosis  intracellular  protein binding  protein processing  protein processing  proteolysis and peptidolysis  regulation of apoptosis  response to hypoxia | MAPK signaling pathway  Huntington's disease  Dentatorubropallidoluysian atrophy (DRPLA) | 3.67884 | 3.82122 | 3.67274 | 3.76663 | 5.41527 | 3.85077 | 5.49885 | 5.48616 | 6.73747 | 6.97565 | 8.5617 | 6.99943 | 6.85343 | 7.62276 | 8.26355 | 8.22892 | 3.86886 | 3.53153 | 3.09868 | -0.190021 | 0.147318 | 0.580165 | -0.0476469 | 0.289692 | 0.72254 | -0.196122 | 0.141217 | 0.574065 | -0.102239 | 0.2351 | 0.667947 | 1.54641 | 1.88375 | 2.3166 | -0.0180911 | 0.319248 | 0.752095 | 1.62999 | 1.96733 | 2.40018 | 1.61729 | 1.95463 | 2.38748 | 2.86861 | 3.20595 | 3.63879 | 3.10678 | 3.44412 | 3.87697 | 4.69283 | 5.03017 | 5.46302 | 3.13057 | 3.46791 | 3.90076 | 2.98456 | 3.3219 | 3.75475 | 3.7539 | 4.09124 | 4.52408 | 4.39468 | 4.73202 | 5.16487 | 4.36005 | 4.69739 | 5.13024 |
| 102070\_at | Col9a3 | procollagen, type IX, alpha 3 | 12841 | 2 | 9 | cell adhesion  collagen  collagen type IX  extracellular matrix  extracellular matrix structural constituent  extracellular matrix structural constituent conferring tensile strength  extracellular space |  | 3.44008 | 3.437 | 3.44537 | 3.39358 | 3.4853 | 3.41668 | 3.50493 | 2.99933 | 7.03478 | 5.78958 | 9.34258 | 8.97301 | 9.36838 | 8.8135 | 8.95515 | 8.43496 | 3.7566 | 3.42761 | 3.62177 | -0.316518 | 0.0124732 | -0.181685 | -0.319604 | 0.00938732 | -0.184771 | -0.311227 | 0.0177642 | -0.176394 | -0.363022 | -0.0340306 | -0.228189 | -0.271299 | 0.0576927 | -0.136466 | -0.33992 | -0.010929 | -0.205087 | -0.251668 | 0.0773229 | -0.116835 | -0.757268 | -0.428276 | -0.622434 | 3.27818 | 3.60717 | 3.41301 | 2.03298 | 2.36197 | 2.16781 | 5.58598 | 5.91498 | 5.72082 | 5.21641 | 5.5454 | 5.35124 | 5.61178 | 5.94077 | 5.74662 | 5.0569 | 5.38589 | 5.19173 | 5.19855 | 5.52754 | 5.33339 | 4.67836 | 5.00735 | 4.8132 |
| 102197\_at | Nucb2 | nucleobindin 2 | 53322 | 7 | 3 | DNA binding  calcium ion binding  calcium ion homeostasis  cytoplasm  endoplasmic reticulum  extracellular space  nuclear outer membrane  nucleus |  | 6.07517 | 6.12478 | 6.25547 | 6.27574 | 8.26184 | 6.36657 | 7.54495 | 6.81314 | 8.31755 | 8.27678 | 9.40778 | 8.73203 | 8.69618 | 8.80689 | 8.58282 | 8.88396 | 8.34339 | 8.75798 | 8.15293 | -2.26822 | -2.6828 | -2.07776 | -2.21862 | -2.6332 | -2.02816 | -2.08793 | -2.50251 | -1.89747 | -2.06765 | -2.48224 | -1.87719 | -0.081556 | -0.49614 | 0.108902 | -1.97683 | -2.39141 | -1.78637 | -0.798446 | -1.21303 | -0.607987 | -1.53025 | -1.94484 | -1.33979 | -0.0258398 | -0.440423 | 0.164618 | -0.0666173 | -0.481201 | 0.123841 | 1.06439 | 0.649804 | 1.25485 | 0.388636 | -0.025948 | 0.579094 | 0.352782 | -0.0618013 | 0.54324 | 0.463498 | 0.0489145 | 0.653956 | 0.239423 | -0.175161 | 0.429881 | 0.540568 | 0.125985 | 0.731027 |
| 102198\_at | Kcnn4 | potassium intermediate/small conductance calcium-activated channel, subfamily N, member 4 | 16534 | 7 | 9 | calcium-activated potassium channel activity  calmodulin binding  integral to membrane  integral to plasma membrane  ion channel activity  ion transport  membrane  positive regulation of protein secretion  potassium channel activity  potassium ion transport  small conductance calcium-activated potassium channel activity |  | 6.97705 | 4.48181 | 8.22982 | 8.86698 | 10.5792 | 8.0027 | 9.52269 | 8.34762 | 11.0771 | 10.7824 | 11.6042 | 11.8485 | 11.97 | 11.6495 | 11.5853 | 12.129 | 10.2106 | 10.3945 | 10.5893 | -3.23353 | -3.41748 | -3.61222 | -5.72877 | -5.91272 | -6.10746 | -1.98076 | -2.16471 | -2.35945 | -1.3436 | -1.52755 | -1.72229 | 0.368626 | 0.184675 | -0.010065 | -2.20788 | -2.39183 | -2.58657 | -0.687886 | -0.871837 | -1.06658 | -1.86296 | -2.04691 | -2.24165 | 0.866517 | 0.682566 | 0.487826 | 0.571838 | 0.387887 | 0.193147 | 1.39363 | 1.20968 | 1.01494 | 1.6379 | 1.45395 | 1.25921 | 1.75943 | 1.57548 | 1.38074 | 1.43893 | 1.25498 | 1.06024 | 1.37467 | 1.19072 | 0.995982 | 1.91838 | 1.73443 | 1.53969 |
| 102207\_at | BC011468 | cDNA sequence BC011468 | 223649 | 15 | 2 |  |  | 3.20289 | 3.07865 | 4.0807 | 3.17105 | 4.19898 | 4.11899 | 4.05838 | 4.09909 | 6.62836 | 5.77827 | 6.73604 | 6.00991 | 6.36587 | 6.75015 | 7.08129 | 6.88153 | 3.9523 | 3.86472 | 2.94685 | -0.749413 | -0.661838 | 0.256034 | -0.873652 | -0.786078 | 0.131795 | 0.128397 | 0.215971 | 1.13384 | -0.781246 | -0.693671 | 0.224201 | 0.246679 | 0.334254 | 1.25213 | 0.16669 | 0.254264 | 1.17214 | 0.106078 | 0.193653 | 1.11153 | 0.146795 | 0.23437 | 1.15224 | 2.67606 | 2.76364 | 3.68151 | 1.82597 | 1.91355 | 2.83142 | 2.78374 | 2.87131 | 3.78919 | 2.05761 | 2.14518 | 3.06306 | 2.41357 | 2.50115 | 3.41902 | 2.79785 | 2.88543 | 3.8033 | 3.12899 | 3.21656 | 4.13443 | 2.92923 | 3.0168 | 3.93468 |
| 102248\_f\_at | Cask | calcium/calmodulin-dependent serine protein kinase | 12361 | X | 9 | ATP binding  basolateral plasma membrane  calmodulin binding  cytosol  membrane  protein amino acid phosphorylation  protein binding  protein kinase activity  protein serine/threonine kinase activity  protein-tyrosine kinase activity  synapse  transferase activity | Starch and sucrose metabolism  Inositol phosphate metabolism  Benzoate degradation via CoA ligation  Nicotinate and nicotinamide metabolism | 2.90211 | 2.96372 | 2.96973 | 2.3217 | 2.95293 | 2.97857 | 3.20606 | 3.04752 | 3.80368 | 4.65129 | 6.0041 | 5.90568 | 4.60374 | 5.63274 | 5.27904 | 6.13959 | 3.30927 | 3.36479 | 4.1293 | -0.407155 | -0.462678 | -1.22719 | -0.34555 | -0.401072 | -1.16558 | -0.339537 | -0.39506 | -1.15957 | -0.98757 | -1.04309 | -1.8076 | -0.356341 | -0.411863 | -1.17637 | -0.330698 | -0.38622 | -1.15073 | -0.103206 | -0.158728 | -0.923236 | -0.261753 | -0.317275 | -1.08178 | 0.494413 | 0.438891 | -0.325617 | 1.34202 | 1.28649 | 0.521987 | 2.69483 | 2.63931 | 1.8748 | 2.59641 | 2.54089 | 1.77638 | 1.29447 | 1.23895 | 0.474438 | 2.32347 | 2.26794 | 1.50344 | 1.96978 | 1.91425 | 1.14975 | 2.83032 | 2.7748 | 2.01029 |
| 102255\_at | Osmr | oncostatin M receptor | 18414 | 15 | 6 | cell surface receptor linked signal transduction  extracellular space  hematopoietin/interferon-class (D200-domain) cytokine receptor activity  integral to membrane  integral to plasma membrane  membrane  oncostatin-M receptor activity  receptor activity | Cytokine-cytokine receptor interaction | 4.31141 | 4.66581 | 4.88132 | 5.31429 | 6.11589 | 5.41938 | 5.22021 | 5.11351 | 6.26222 | 6.2832 | 6.80518 | 6.49745 | 6.2292 | 6.63981 | 6.87068 | 6.51268 | 2.87588 | 3.10407 | 3.0269 | 1.43553 | 1.20734 | 1.28451 | 1.78992 | 1.56174 | 1.63891 | 2.00544 | 1.77725 | 1.85443 | 2.43841 | 2.21022 | 2.28739 | 3.24001 | 3.01182 | 3.08899 | 2.5435 | 2.31531 | 2.39248 | 2.34433 | 2.11614 | 2.19331 | 2.23763 | 2.00944 | 2.08662 | 3.38634 | 3.15815 | 3.23533 | 3.40732 | 3.17913 | 3.2563 | 3.9293 | 3.70111 | 3.77828 | 3.62157 | 3.39338 | 3.47056 | 3.35332 | 3.12513 | 3.2023 | 3.76393 | 3.53574 | 3.61291 | 3.9948 | 3.76661 | 3.84379 | 3.6368 | 3.40861 | 3.48578 |
| 102381\_at | Acsl4 | acyl-CoA synthetase long-chain family member 4 | 50790 | X | 9 | catalytic activity  fatty acid metabolism  integral to membrane  ligase activity  long-chain-fatty-acid-CoA ligase activity  magnesium ion binding  metabolism  regulation of fatty acid metabolism | Fatty acid metabolism | 7.1637 | 7.44458 | 9.0802 | 7.97706 | 10.6878 | 9.75775 | 9.95392 | 9.78773 | 11.6576 | 11.5137 | 12.3395 | 12.3015 | 12.0118 | 12.5894 | 12.094 | 12.3228 | 10.3019 | 10.7447 | 9.99969 | -3.13823 | -3.58103 | -2.83598 | -2.85736 | -3.30016 | -2.55511 | -1.22174 | -1.66454 | -0.919487 | -2.32488 | -2.76768 | -2.02263 | 0.385896 | -0.0569037 | 0.688146 | -0.544188 | -0.986987 | -0.241938 | -0.348011 | -0.790811 | -0.0457614 | -0.514203 | -0.957003 | -0.211953 | 1.35562 | 0.912825 | 1.65787 | 1.21172 | 0.768922 | 1.51397 | 2.03753 | 1.59473 | 2.33978 | 1.99952 | 1.55672 | 2.30177 | 1.70991 | 1.26711 | 2.01216 | 2.28744 | 1.84464 | 2.58969 | 1.7921 | 1.3493 | 2.09435 | 2.02089 | 1.57809 | 2.32314 |
| 102410\_at | Hs3st1 | heparan sulfate (glucosamine) 3-O-sulfotransferase 1 | 15476 | 5 | 10 | extracellular space  sulfotransferase activity  transferase activity |  | 3.58496 | 3.64674 | 3.6895 | 3.58131 | 3.65553 | 3.65761 | 3.7157 | 3.66496 | 7.95748 | 8.35782 | 9.79941 | 10.1509 | 9.8952 | 10.1033 | 9.29522 | 10.0406 | 3.54299 | 3.55685 | 3.49603 | 0.0419724 | 0.028112 | 0.0889309 | 0.103752 | 0.0898914 | 0.15071 | 0.146513 | 0.132653 | 0.193472 | 0.0383182 | 0.0244579 | 0.0852768 | 0.112539 | 0.0986787 | 0.159498 | 0.114617 | 0.100757 | 0.161576 | 0.172706 | 0.158846 | 0.219665 | 0.121972 | 0.108112 | 0.168931 | 4.41449 | 4.40063 | 4.46145 | 4.81483 | 4.80097 | 4.86179 | 6.25642 | 6.24256 | 6.30337 | 6.60796 | 6.5941 | 6.65492 | 6.35221 | 6.33835 | 6.39917 | 6.56028 | 6.54642 | 6.60724 | 5.75223 | 5.73837 | 5.79919 | 6.49759 | 6.48372 | 6.54454 |
| 102414\_i\_at | Dnajc3 | DnaJ (Hsp40) homolog, subfamily C, member 3 | 19107 | 14 | 8 | cytoplasm  extracellular space  negative regulation of protein kinase activity  protein kinase inhibitor activity |  | 6.27781 | 6.35322 | 5.6048 | 6.09714 | 6.25637 | 6.91434 | 7.40543 | 7.08005 | 8.23382 | 7.12034 | 7.05332 | 7.47807 | 7.04953 | 8.02929 | 8.94538 | 8.56109 | 7.04017 | 7.37396 | 6.30585 | -0.762358 | -1.09615 | -0.0280456 | -0.686942 | -1.02073 | 0.0473713 | -1.43536 | -1.76916 | -0.70105 | -0.943031 | -1.27682 | -0.208718 | -0.783796 | -1.11759 | -0.049483 | -0.125823 | -0.459616 | 0.60849 | 0.365264 | 0.0314706 | 1.09958 | 0.0398796 | -0.293914 | 0.774192 | 1.19365 | 0.859856 | 1.92796 | 0.0801789 | -0.253615 | 0.814492 | 0.0131555 | -0.320638 | 0.747468 | 0.437902 | 0.104108 | 1.17221 | 0.00936002 | -0.324433 | 0.743673 | 0.989129 | 0.655335 | 1.72344 | 1.90522 | 1.57142 | 2.63953 | 1.52092 | 1.18713 | 2.25523 |
| 102804\_at | Ceacam1 | CEA-related cell adhesion molecule 1 | 26365 | 7 | 22 | integral to membrane  receptor activity |  | 0.345872 | 0.385433 | 0.426788 | 0.331201 | 0.353889 | 0.392749 | 0.374159 | 0.391613 | 3.29378 | 4.10426 | 4.46919 | 4.70331 | 4.53059 | 5.13485 | 6.97907 | 6.02679 | 2.3992 | 2.3633 | 0.359077 | -2.05333 | -2.01743 | -0.0132058 | -2.01377 | -1.97787 | 0.0263559 | -1.97241 | -1.93651 | 0.0677103 | -2.068 | -2.0321 | -0.0278768 | -2.04531 | -2.00941 | -0.00518852 | -2.00645 | -1.97055 | 0.0336714 | -2.02504 | -1.98914 | 0.015082 | -2.00759 | -1.97169 | 0.0325354 | 0.89458 | 0.93048 | 2.9347 | 1.70506 | 1.74096 | 3.74518 | 2.06999 | 2.10589 | 4.11011 | 2.30411 | 2.34001 | 4.34423 | 2.13139 | 2.16729 | 4.17151 | 2.73565 | 2.77155 | 4.77578 | 4.57986 | 4.61576 | 6.61999 | 3.62759 | 3.66349 | 5.66771 |
| 102805\_at | Ceacam1 | CEA-related cell adhesion molecule 1 | 26365 | 7 | 22 | integral to membrane  receptor activity |  | 1.54065 | 2.33162 | 3.10938 | 1.52981 | 2.22149 | 2.47921 | 1.5659 | 1.61526 | 8.3513 | 9.82685 | 8.21871 | 8.90489 | 7.09124 | 10.2556 | 10.3012 | 10.4559 | 7.09146 | 6.44176 | 2.16574 | -5.55081 | -4.90111 | -0.625085 | -4.75984 | -4.11014 | 0.165881 | -3.98208 | -3.33238 | 0.943639 | -5.56165 | -4.91195 | -0.635924 | -4.86997 | -4.22027 | 0.055756 | -4.61225 | -3.96255 | 0.313476 | -5.52556 | -4.87586 | -0.59984 | -5.4762 | -4.8265 | -0.550478 | 1.25984 | 1.90954 | 6.18556 | 2.73539 | 3.38509 | 7.66111 | 1.12725 | 1.77695 | 6.05298 | 1.81343 | 2.46313 | 6.73915 | -0.000219088 | 0.649482 | 4.9255 | 3.16415 | 3.81385 | 8.08987 | 3.2097 | 3.8594 | 8.13542 | 3.36448 | 4.01418 | 8.29021 |
| 102806\_g\_at | Ceacam1 | CEA-related cell adhesion molecule 1 | 26365 | 7 | 22 | integral to membrane  receptor activity |  | 2.66058 | 2.75858 | 3.69684 | 2.65883 | 2.89613 | 3.70961 | 2.69537 | 2.74055 | 8.25639 | 8.41941 | 7.52308 | 8.28967 | 7.48113 | 9.35864 | 9.94508 | 10.0728 | 7.48404 | 6.80907 | 2.76712 | -4.82346 | -4.14849 | -0.106536 | -4.72546 | -4.05049 | -0.00854136 | -3.7872 | -3.11223 | 0.929716 | -4.8252 | -4.15023 | -0.108285 | -4.58791 | -3.91294 | 0.129007 | -3.77443 | -3.09946 | 0.942493 | -4.78867 | -4.1137 | -0.0717511 | -4.74349 | -4.06852 | -0.0265706 | 0.772351 | 1.44732 | 5.48927 | 0.935373 | 1.61034 | 5.65229 | 0.0390427 | 0.714013 | 4.75596 | 0.805631 | 1.4806 | 5.52255 | -0.0029139 | 0.672056 | 4.71401 | 1.8746 | 2.54957 | 6.59152 | 2.46104 | 3.13601 | 7.17796 | 2.58879 | 3.26376 | 7.30571 |
| 102856\_at | Sox10 | SRY-box containing gene 10 | 20665 | 15 | 13 | DNA binding  cell differentiation  nucleus  regulation of transcription, DNA-dependent  transcription factor activity  transcription factor complex |  | 2.92994 | 2.7256 | 3.39984 | 4.38391 | 8.34569 | 3.37117 | 6.65903 | 3.40061 | 9.37109 | 8.30739 | 10.3392 | 10.3649 | 11.116 | 10.1877 | 10.707 | 10.7366 | 8.20728 | 8.57073 | 8.05837 | -5.27734 | -5.64079 | -5.12843 | -5.48168 | -5.84512 | -5.33277 | -4.80744 | -5.17088 | -4.65853 | -3.82337 | -4.18681 | -3.67446 | 0.138406 | -0.225041 | 0.287312 | -4.83611 | -5.19956 | -4.6872 | -1.54825 | -1.9117 | -1.39935 | -4.80667 | -5.17012 | -4.65777 | 1.16381 | 0.800363 | 1.31272 | 0.100113 | -0.263335 | 0.249019 | 2.13193 | 1.76848 | 2.28084 | 2.15766 | 1.79421 | 2.30657 | 2.90869 | 2.54524 | 3.05759 | 1.98045 | 1.61701 | 2.12936 | 2.49973 | 2.13628 | 2.64863 | 2.52929 | 2.16584 | 2.67819 |
| 102936\_at | B4galt6 | UDP-Gal | 56386 | 18 | 4 | Golgi apparatus  carbohydrate metabolism  galactosyltransferase activity  integral to membrane  magnesium ion binding  manganese ion binding  sphingolipid biosynthesis  transferase activity  transferase activity, transferring glycosyl groups | Fructose and mannose metabolism  N-Glycans biosynthesis  O-Glycans biosynthesis  Glycerolipid metabolism  Glycosylphosphatidylinositol(GPI)-anchor biosynthesis  Glycosphingolipid metabolism  Blood group glycolipid biosynthesis-lactoseries  Blood group glycolipid biosynthesis-neolactoseries  Globoside metabolism  Ganglioside biosynthesis | 3.16307 | 3.26428 | 3.46329 | 3.94903 | 6.88998 | 3.45052 | 4.14602 | 3.48572 | 6.97899 | 8.16929 | 9.22629 | 8.57416 | 8.01787 | 8.69162 | 8.74978 | 9.37716 | 3.26708 | 3.19659 | 3.18049 | -0.104009 | -0.0335209 | -0.0174252 | -0.00280205 | 0.0676862 | 0.0837819 | 0.196206 | 0.266695 | 0.28279 | 0.681954 | 0.752442 | 0.768538 | 3.6229 | 3.69339 | 3.70949 | 0.183442 | 0.25393 | 0.270026 | 0.878945 | 0.949433 | 0.965529 | 0.218642 | 0.28913 | 0.305226 | 3.71191 | 3.7824 | 3.7985 | 4.90221 | 4.9727 | 4.9888 | 5.95921 | 6.0297 | 6.04579 | 5.30708 | 5.37757 | 5.39366 | 4.75079 | 4.82128 | 4.83737 | 5.42454 | 5.49503 | 5.51113 | 5.4827 | 5.55318 | 5.56928 | 6.11008 | 6.18057 | 6.19666 |
| 102952\_g\_at | Cradd | CASP2 and RIPK1 domain containing adaptor with death domain | 12905 | 10 | 6 | apoptosis  intracellular  protein binding  regulation of apoptosis  signal transduction |  | 2.73892 | 2.8223 | 2.85526 | 2.73606 | 2.77253 | 2.82491 | 2.80114 | 2.81856 | 2.86426 | 4.39943 | 5.52539 | 5.68575 | 5.30857 | 5.36885 | 5.54439 | 6.06795 | 2.69998 | 2.71218 | 2.67421 | 0.0389341 | 0.0267342 | 0.0647071 | 0.12232 | 0.11012 | 0.148093 | 0.155282 | 0.143082 | 0.181054 | 0.0360737 | 0.0238738 | 0.0618466 | 0.0725447 | 0.0603448 | 0.0983176 | 0.124928 | 0.112729 | 0.150701 | 0.101159 | 0.088959 | 0.126932 | 0.118578 | 0.106379 | 0.144351 | 0.164274 | 0.152074 | 0.190047 | 1.69945 | 1.68725 | 1.72522 | 2.82541 | 2.81321 | 2.85118 | 2.98577 | 2.97357 | 3.01154 | 2.60859 | 2.59639 | 2.63436 | 2.66887 | 2.65667 | 2.69464 | 2.84441 | 2.83221 | 2.87018 | 3.36797 | 3.35577 | 3.39375 |
| 102979\_at |  |  |  |  |  |  |  | 3.72015 | 3.80549 | 3.85108 | 3.72971 | 3.75191 | 3.8347 | 3.77052 | 3.82086 | 4.35329 | 7.52659 | 9.66867 | 9.37016 | 9.77002 | 9.0956 | 9.35481 | 9.9794 | 3.71709 | 3.71706 | 3.66536 | 0.00305549 | 0.00308588 | 0.0547901 | 0.0883962 | 0.0884266 | 0.140131 | 0.133994 | 0.134024 | 0.185729 | 0.0126225 | 0.0126529 | 0.0643571 | 0.0348232 | 0.0348536 | 0.0865578 | 0.117606 | 0.117637 | 0.169341 | 0.0534345 | 0.0534649 | 0.105169 | 0.103775 | 0.103805 | 0.155509 | 0.636198 | 0.636228 | 0.687933 | 3.8095 | 3.80953 | 3.86124 | 5.95158 | 5.95161 | 6.00331 | 5.65307 | 5.6531 | 5.7048 | 6.05293 | 6.05296 | 6.10467 | 5.37851 | 5.37854 | 5.43025 | 5.63772 | 5.63775 | 5.68946 | 6.26231 | 6.26234 | 6.31405 |
| 102993\_at | Ggta1 | glycoprotein galactosyltransferase alpha 1, 3 | 14594 | 2 | 9 | Golgi apparatus  N-acetyllactosaminide 3-alpha-galactosyltransferase activity  carbohydrate metabolism  integral to membrane  manganese ion binding  membrane  transferase activity  transferase activity, transferring glycosyl groups  transferase activity, transferring hexosyl groups | Blood group glycolipid biosynthesis-lactoseries  Blood group glycolipid biosynthesis-neolactoseries | 4.34548 | 4.02242 | 4.10434 | 4.18281 | 4.09188 | 4.05158 | 5.465 | 5.4201 | 8.31226 | 8.83248 | 9.6268 | 9.74552 | 9.69997 | 9.72599 | 10.4037 | 10.5046 | 3.9558 | 3.59848 | 3.63577 | 0.389684 | 0.746995 | 0.709713 | 0.0666228 | 0.423934 | 0.386652 | 0.14854 | 0.505851 | 0.468569 | 0.227016 | 0.584327 | 0.547045 | 0.136088 | 0.493399 | 0.456117 | 0.0957848 | 0.453096 | 0.415814 | 1.50921 | 1.86652 | 1.82924 | 1.4643 | 1.82161 | 1.78433 | 4.35646 | 4.71378 | 4.67649 | 4.87668 | 5.23399 | 5.19671 | 5.67101 | 6.02832 | 5.99104 | 5.78973 | 6.14704 | 6.10976 | 5.74418 | 6.10149 | 6.0642 | 5.7702 | 6.12751 | 6.09023 | 6.44789 | 6.8052 | 6.76792 | 6.54883 | 6.90614 | 6.86886 |
| 103051\_at | Expi | extracellular proteinase inhibitor | 14038 | 11 | 3 | endopeptidase inhibitor activity  extracellular space |  | 10.0862 | 9.6048 | 11.1093 | 11.8523 | 12.799 | 11.854 | 12.4992 | 11.6026 | 12.9143 | 12.7839 | 13.2912 | 13.4011 | 13.4817 | 13.0683 | 13.2262 | 13.4833 | 13.4626 | 13.3759 | 13.3259 | -3.37639 | -3.28974 | -3.23972 | -3.85777 | -3.77112 | -3.7211 | -2.35332 | -2.26667 | -2.21665 | -1.61027 | -1.52362 | -1.4736 | -0.663603 | -0.576952 | -0.526931 | -1.60855 | -1.5219 | -1.47188 | -0.963345 | -0.876694 | -0.826673 | -1.86001 | -1.77336 | -1.72334 | -0.548259 | -0.461607 | -0.411587 | -0.678699 | -0.592048 | -0.542027 | -0.171373 | -0.0847219 | -0.0347013 | -0.0615168 | 0.0251346 | 0.0751552 | 0.0191171 | 0.105768 | 0.155789 | -0.394229 | -0.307578 | -0.257557 | -0.236416 | -0.149765 | -0.0997443 | 0.0207008 | 0.107352 | 0.157373 |
| 103059\_at | Fxyd3 | FXYD domain-containing ion transport regulator 3 | 17178 | 7 | 6 | extracellular space  integral to membrane  ion channel activity  ion transport  membrane |  | 8.09265 | 8.86264 | 10.4129 | 10.2368 | 11.6653 | 10.1638 | 11.1137 | 9.58746 | 12.5133 | 12.1913 | 12.538 | 12.8545 | 12.7782 | 12.6475 | 13.1428 | 13.1386 | 10.5197 | 10.4282 | 11.0588 | -2.42707 | -2.33559 | -2.96615 | -1.65708 | -1.5656 | -2.19617 | -0.1068 | -0.0153196 | -0.645886 | -0.28293 | -0.191449 | -0.822015 | 1.14554 | 1.23702 | 0.606454 | -0.355898 | -0.264417 | -0.894983 | 0.593957 | 0.685438 | 0.054872 | -0.932259 | -0.840778 | -1.47134 | 1.99359 | 2.08507 | 1.45451 | 1.67157 | 1.76306 | 1.13249 | 2.01828 | 2.10976 | 1.47919 | 2.33475 | 2.42623 | 1.79566 | 2.25848 | 2.34996 | 1.7194 | 2.12782 | 2.2193 | 1.58874 | 2.62308 | 2.71456 | 2.084 | 2.61884 | 2.71032 | 2.07976 |
| 103065\_at | Slc20a1 | solute carrier family 20, member 1 | 20515 | 2 | 7 | carrier activity  extracellular space  inorganic phosphate transporter activity  integral to membrane  integral to plasma membrane  membrane  phosphate transport  receptor activity  transport |  | 4.69936 | 4.18387 | 4.13045 | 4.36109 | 5.09729 | 4.69345 | 5.14846 | 5.19057 | 5.58259 | 5.82797 | 7.32517 | 6.47377 | 6.23578 | 5.82895 | 6.08271 | 6.38424 | 5.07015 | 5.11558 | 3.76107 | -0.370792 | -0.416215 | 0.938293 | -0.886288 | -0.93171 | 0.422797 | -0.939706 | -0.985128 | 0.369379 | -0.709059 | -0.754482 | 0.600026 | 0.0271362 | -0.0182864 | 1.33622 | -0.376701 | -0.422124 | 0.932384 | 0.078301 | 0.0328785 | 1.38739 | 0.120414 | 0.0749918 | 1.4295 | 0.512433 | 0.46701 | 1.82152 | 0.757813 | 0.712391 | 2.0669 | 2.25502 | 2.2096 | 3.56411 | 1.40362 | 1.3582 | 2.7127 | 1.16562 | 1.1202 | 2.47471 | 0.758797 | 0.713375 | 2.06788 | 1.01256 | 0.967134 | 2.32164 | 1.31408 | 1.26866 | 2.62317 |
| 103202\_at | Gbp4 | guanylate nucleotide binding protein 4 | 55932 | 3 | 4 | GTP binding  GTPase activity  cytosol  immune response |  | 8.16748 | 7.20161 | 7.92644 | 8.00173 | 8.84954 | 8.0598 | 9.46938 | 8.91551 | 8.36228 | 9.7638 | 10.6712 | 9.86081 | 9.61732 | 9.81059 | 10.5302 | 10.6471 | 7.52277 | 6.28244 | 5.03367 | 0.644707 | 1.88504 | 3.13381 | -0.321162 | 0.919171 | 2.16794 | 0.403667 | 1.644 | 2.89277 | 0.478957 | 1.71929 | 2.96806 | 1.32677 | 2.56711 | 3.81587 | 0.53703 | 1.77736 | 3.02613 | 1.94661 | 3.18694 | 4.43571 | 1.39274 | 2.63307 | 3.88184 | 0.839512 | 2.07985 | 3.32861 | 2.24103 | 3.48136 | 4.73012 | 3.14841 | 4.38874 | 5.63751 | 2.33804 | 3.57837 | 4.82714 | 2.09455 | 3.33488 | 4.58365 | 2.28782 | 3.52815 | 4.77692 | 3.00744 | 4.24778 | 5.49654 | 3.12433 | 4.36466 | 5.61343 |
| 103305\_at | Itgb4 | integrin beta 4 | 192897 | 11 | 10 | basal plasma membrane  cell adhesion  cell adhesion  cell-matrix adhesion  development  integral to membrane  integrin complex  integrin complex  integrin-mediated signaling pathway  protein binding  receptor activity |  | 2.51859 | 3.05433 | 5.37854 | 3.88005 | 6.30171 | 5.72242 | 4.40474 | 2.72011 | 8.32616 | 7.55823 | 9.23063 | 9.77615 | 9.89661 | 9.51474 | 9.66961 | 9.50868 | 5.6837 | 5.50881 | 6.10375 | -3.16511 | -2.99022 | -3.58515 | -2.62937 | -2.45448 | -3.04942 | -0.30516 | -0.130271 | -0.725207 | -1.80365 | -1.62876 | -2.2237 | 0.618011 | 0.792899 | 0.197963 | 0.0387249 | 0.213613 | -0.381323 | -1.27896 | -1.10407 | -1.69901 | -2.96359 | -2.7887 | -3.38364 | 2.64246 | 2.81735 | 2.22241 | 1.87454 | 2.04942 | 1.45449 | 3.54693 | 3.72182 | 3.12688 | 4.09245 | 4.26734 | 3.6724 | 4.21291 | 4.3878 | 3.79286 | 3.83104 | 4.00593 | 3.41099 | 3.98591 | 4.1608 | 3.56587 | 3.82498 | 3.99987 | 3.40494 |
| 103345\_at | Spna2 | spectrin alpha 2 | 20740 | 2 | 8 | actin binding  calcium ion binding  calmodulin binding  cytoskeleton  membrane |  | 9.27626 | 8.8865 | 9.37191 | 8.80018 | 9.59348 | 9.50792 | 9.32199 | 9.34637 | 10.8915 | 10.9105 | 11.4824 | 11.3344 | 11.5228 | 11.7099 | 11.6593 | 11.3983 | 8.71568 | 8.10296 | 8.17714 | 0.56058 | 1.1733 | 1.09912 | 0.170823 | 0.783541 | 0.709362 | 0.656231 | 1.26895 | 1.19477 | 0.0845041 | 0.697223 | 0.623043 | 0.877808 | 1.49053 | 1.41635 | 0.792242 | 1.40496 | 1.33078 | 0.606312 | 1.21903 | 1.14485 | 0.630693 | 1.24341 | 1.16923 | 2.17584 | 2.78856 | 2.71438 | 2.19486 | 2.80758 | 2.7334 | 2.76676 | 3.37947 | 3.30529 | 2.61874 | 3.23146 | 3.15728 | 2.80708 | 3.4198 | 3.34562 | 2.99421 | 3.60693 | 3.53275 | 2.94357 | 3.55629 | 3.48211 | 2.68262 | 3.29534 | 3.22116 |
| 103549\_at | Nes | nestin | 18008 | 3 | 18 | cytoplasm  intermediate filament  neurogenesis |  | 2.23369 | 2.29293 | 2.33364 | 2.22968 | 3.24297 | 2.31444 | 2.32727 | 2.30271 | 6.48779 | 6.23188 | 7.0015 | 7.2425 | 7.76426 | 7.0988 | 6.65481 | 7.64716 | 2.19068 | 2.20511 | 2.1762 | 0.0430012 | 0.0285755 | 0.0574823 | 0.102244 | 0.0878187 | 0.116726 | 0.142955 | 0.128529 | 0.157436 | 0.038995 | 0.0245693 | 0.0534761 | 1.05228 | 1.03786 | 1.06676 | 0.123758 | 0.109332 | 0.138239 | 0.136586 | 0.12216 | 0.151067 | 0.112029 | 0.0976028 | 0.12651 | 4.29711 | 4.28268 | 4.31159 | 4.0412 | 4.02677 | 4.05568 | 4.81081 | 4.79639 | 4.82529 | 5.05182 | 5.03739 | 5.0663 | 5.57357 | 5.55915 | 5.58805 | 4.90811 | 4.89369 | 4.92259 | 4.46412 | 4.4497 | 4.4786 | 5.45648 | 5.44206 | 5.47096 |
| 103721\_at | Npnt | nephronectin | 114249 | 3 | 7 | calcium ion binding  cell-matrix adhesion  cell-matrix adhesion  extracellular matrix  extracellular matrix  extracellular space  integrin binding  membrane |  | 2.8983 | 3.09129 | 5.14723 | 4.53467 | 7.79654 | 6.14604 | 7.21024 | 5.35162 | 9.96315 | 10.016 | 11.0344 | 10.3928 | 10.8858 | 10.8371 | 10.7668 | 11.0881 | 4.95784 | 5.30874 | 3.60686 | -2.05953 | -2.41043 | -0.708556 | -1.86655 | -2.21745 | -0.515573 | 0.189387 | -0.16151 | 1.54036 | -0.423167 | -0.774064 | 0.927811 | 2.8387 | 2.4878 | 4.18968 | 1.18821 | 0.837308 | 2.53918 | 2.25241 | 1.90151 | 3.60338 | 0.39378 | 0.0428827 | 1.74476 | 5.00531 | 4.65441 | 6.35628 | 5.05819 | 4.70729 | 6.40916 | 6.07658 | 5.72568 | 7.42756 | 5.43494 | 5.08404 | 6.78591 | 5.92798 | 5.57708 | 7.27895 | 5.87924 | 5.52834 | 7.23021 | 5.80892 | 5.45802 | 7.1599 | 6.1303 | 5.7794 | 7.48128 |
| 103812\_at | Clca1 | chloride channel calcium activated 1 | 12722 | 3 | 15 | chloride transport  integral to plasma membrane  intracellular calcium activated chloride channel activity |  | 4.15951 | 4.47364 | 5.87032 | 4.80093 | 6.92429 | 4.74255 | 8.14412 | 5.66635 | 9.29686 | 9.48236 | 9.7762 | 9.57385 | 9.2532 | 10.0332 | 9.75257 | 10.5766 | 3.92425 | 3.86546 | 3.78949 | 0.235257 | 0.294045 | 0.370018 | 0.549393 | 0.608181 | 0.684154 | 1.94607 | 2.00486 | 2.08083 | 0.876683 | 0.935471 | 1.01144 | 3.00004 | 3.05883 | 3.1348 | 0.818297 | 0.877086 | 0.953058 | 4.21987 | 4.27865 | 4.35463 | 1.7421 | 1.80088 | 1.87686 | 5.37261 | 5.43139 | 5.50737 | 5.55811 | 5.61689 | 5.69287 | 5.85195 | 5.91074 | 5.98671 | 5.6496 | 5.70839 | 5.78436 | 5.32895 | 5.38774 | 5.46371 | 6.10898 | 6.16777 | 6.24375 | 5.82832 | 5.88711 | 5.96308 | 6.65231 | 6.71109 | 6.78707 |
| 103816\_at | F11r | F11 receptor | 16456 | 1 | 8 | cell adhesion  extracellular space  integral to membrane  plasma membrane  protein binding  tight junction |  | 7.1314 | 6.95458 | 7.9812 | 7.66373 | 8.35187 | 7.57942 | 8.1832 | 7.01686 | 9.01236 | 9.50712 | 9.14147 | 9.57908 | 9.24589 | 9.88943 | 9.80905 | 9.76529 | 8.56687 | 8.59011 | 8.70277 | -1.43547 | -1.4587 | -1.57137 | -1.61229 | -1.63553 | -1.74819 | -0.585679 | -0.608911 | -0.721575 | -0.903141 | -0.926373 | -1.03904 | -0.215008 | -0.23824 | -0.350904 | -0.987457 | -1.01069 | -1.12335 | -0.383677 | -0.406909 | -0.519573 | -1.55002 | -1.57325 | -1.68591 | 0.445488 | 0.422255 | 0.309591 | 0.940249 | 0.917017 | 0.804353 | 0.574591 | 0.551359 | 0.438695 | 1.0122 | 0.98897 | 0.876306 | 0.679012 | 0.65578 | 0.543116 | 1.32256 | 1.29932 | 1.18666 | 1.24218 | 1.21894 | 1.10628 | 1.19841 | 1.17518 | 1.06251 |
| 103818\_at | Slc7a7 | solute carrier family 7 (cationic amino acid transporter, y+ system), member 7 | 20540 |  | 3 | amino acid metabolism  amino acid transport  amino acid-polyamine transporter activity  basic amino acid permease activity  carrier activity  integral to membrane  integral to plasma membrane  membrane  plasma membrane  protein complex assembly  transport  transport |  | 3.34553 | 3.31839 | 3.3547 | 3.426 | 3.69085 | 3.32668 | 3.76043 | 3.3246 | 5.17775 | 5.03776 | 5.79152 | 6.27145 | 6.24304 | 5.99122 | 6.75902 | 5.90648 | 3.49412 | 3.53397 | 3.48654 | -0.148588 | -0.188444 | -0.141012 | -0.175726 | -0.215582 | -0.168151 | -0.139411 | -0.179267 | -0.131836 | -0.0681178 | -0.107974 | -0.0605424 | 0.196733 | 0.156877 | 0.204309 | -0.167438 | -0.207294 | -0.159862 | 0.266312 | 0.226456 | 0.273888 | -0.169518 | -0.209375 | -0.161943 | 1.68364 | 1.64378 | 1.69121 | 1.54364 | 1.50378 | 1.55122 | 2.2974 | 2.25754 | 2.30498 | 2.77734 | 2.73748 | 2.78491 | 2.74892 | 2.70907 | 2.7565 | 2.49711 | 2.45725 | 2.50468 | 3.2649 | 3.22504 | 3.27247 | 2.41236 | 2.3725 | 2.41994 |
| 104017\_at | Acsl4 | acyl-CoA synthetase long-chain family member 4 | 50790 | X | 9 | catalytic activity  fatty acid metabolism  integral to membrane  ligase activity  long-chain-fatty-acid-CoA ligase activity  magnesium ion binding  metabolism  regulation of fatty acid metabolism | Fatty acid metabolism | 4.33195 | 4.4438 | 4.519 | 4.35443 | 4.36201 | 4.73165 | 4.50768 | 4.73265 | 8.60988 | 8.22007 | 7.91817 | 8.27509 | 6.66499 | 9.50154 | 7.66549 | 8.94507 | 4.59807 | 4.51988 | 4.25515 | -0.266122 | -0.187928 | 0.0767957 | -0.154269 | -0.0760742 | 0.18865 | -0.0790717 | -0.000877398 | 0.263846 | -0.243644 | -0.16545 | 0.0992742 | -0.236064 | -0.15787 | 0.106854 | 0.133581 | 0.211776 | 0.476499 | -0.0903956 | -0.0122013 | 0.252522 | 0.134577 | 0.212772 | 0.477495 | 4.01181 | 4.09 | 4.35473 | 3.62199 | 3.70019 | 3.96491 | 3.32009 | 3.39829 | 3.66301 | 3.67702 | 3.75522 | 4.01994 | 2.06692 | 2.14511 | 2.40984 | 4.90346 | 4.98166 | 5.24638 | 3.06742 | 3.14562 | 3.41034 | 4.347 | 4.42519 | 4.68992 |
| 104033\_at | Itm2b | integral membrane protein 2B | 16432 | 14 | 12 | ATP binding  biological\_process unknown  integral to membrane  membrane fraction |  | 8.21827 | 8.38721 | 8.82931 | 8.61074 | 8.72721 | 8.89306 | 8.91831 | 9.03323 | 8.71191 | 9.55089 | 9.63416 | 9.55668 | 9.10436 | 9.64641 | 9.92774 | 9.77666 | 8.5222 | 8.83504 | 8.76032 | -0.303925 | -0.616768 | -0.542046 | -0.134994 | -0.447837 | -0.373115 | 0.307108 | -0.00573545 | 0.0689865 | 0.0885373 | -0.224306 | -0.149584 | 0.205012 | -0.107831 | -0.0331093 | 0.370859 | 0.0580155 | 0.132737 | 0.396108 | 0.0832653 | 0.157987 | 0.511027 | 0.198183 | 0.272905 | 0.189711 | -0.123132 | -0.0484098 | 1.02869 | 0.715849 | 0.790571 | 1.11196 | 0.799122 | 0.873844 | 1.03448 | 0.721636 | 0.796358 | 0.582162 | 0.269319 | 0.344041 | 1.12421 | 0.811366 | 0.886088 | 1.40554 | 1.09269 | 1.16742 | 1.25446 | 0.941622 | 1.01634 |
| 104063\_at | Srcasm | Src activating and signaling molecule | 71943 | 11 | 7 | Golgi apparatus  Golgi stack  intra-Golgi transport  intracellular protein transport  membrane  protein binding  protein kinase activator activity  protein transport  protein transporter activity  transmembrane receptor protein tyrosine kinase signaling pathway  transport |  | 3.6171 | 4.28878 | 6.00964 | 3.83668 | 5.88293 | 4.0781 | 5.45531 | 3.78657 | 9.02734 | 9.09209 | 9.81253 | 9.34122 | 8.96804 | 9.67523 | 10.2357 | 9.28994 | 6.9744 | 7.2185 | 4.75411 | -3.3573 | -3.6014 | -1.13701 | -2.68562 | -2.92972 | -0.465336 | -0.964763 | -1.20887 | 1.25552 | -3.13772 | -3.38183 | -0.917439 | -1.09147 | -1.33557 | 1.12882 | -2.89629 | -3.1404 | -0.676009 | -1.51909 | -1.76319 | 0.701197 | -3.18783 | -3.43193 | -0.967541 | 2.05295 | 1.80884 | 4.27323 | 2.11769 | 1.87359 | 4.33798 | 2.83814 | 2.59403 | 5.05842 | 2.36682 | 2.12272 | 4.58711 | 1.99364 | 1.74953 | 4.21392 | 2.70084 | 2.45673 | 4.92112 | 3.26128 | 3.01718 | 5.48157 | 2.31554 | 2.07144 | 4.53583 |
| 104099\_at | Pglyrp1 | peptidoglycan recognition protein 1 | 21946 | 7 | 13 | N-acetylmuramoyl-L-alanine amidase activity  apoptosis  cytokine activity  extracellular space  immune response  peptidoglycan catabolism  peptidoglycan receptor activity  xenobiotic metabolism |  | 2.165 | 2.20785 | 2.24035 | 2.15135 | 4.44128 | 2.2175 | 4.35357 | 2.39423 | 9.69512 | 9.16786 | 10.233 | 11.0631 | 11.1572 | 10.9653 | 11.6085 | 10.94 | 2.60405 | 2.41731 | 2.35237 | -0.439047 | -0.25231 | -0.187368 | -0.396195 | -0.209458 | -0.144516 | -0.363696 | -0.176959 | -0.112016 | -0.4527 | -0.265963 | -0.201021 | 1.83723 | 2.02397 | 2.08891 | -0.386547 | -0.19981 | -0.134868 | 1.74952 | 1.93626 | 2.0012 | -0.209812 | -0.023075 | 0.0418671 | 7.09108 | 7.27781 | 7.34276 | 6.56382 | 6.75055 | 6.8155 | 7.629 | 7.81574 | 7.88068 | 8.45904 | 8.64578 | 8.71072 | 8.55312 | 8.73986 | 8.8048 | 8.36127 | 8.54801 | 8.61295 | 9.00444 | 9.19118 | 9.25612 | 8.33592 | 8.52266 | 8.5876 |
| 104121\_at | Jup | junction plakoglobin | 16480 | 11 | 11 | cell adhesion  cell-cell adherens junction  cytoskeleton  cytosol  desmosome  intercellular junction  protein binding  structural molecule activity |  | 1.46631 | 2.89795 | 3.58384 | 4.1932 | 4.60967 | 4.20801 | 2.97151 | 3.16177 | 6.5297 | 5.98577 | 6.49517 | 8.30743 | 7.72818 | 8.02799 | 7.77373 | 8.3507 | 4.65298 | 4.23623 | 4.14803 | -3.18667 | -2.76992 | -2.68172 | -1.75503 | -1.33828 | -1.25008 | -1.06914 | -0.652385 | -0.564184 | -0.459785 | -0.0430326 | 0.0451676 | -0.0433076 | 0.373445 | 0.461645 | -0.444969 | -0.0282165 | 0.0599837 | -1.68147 | -1.26471 | -1.17651 | -1.49121 | -1.07445 | -0.986254 | 1.87672 | 2.29348 | 2.38168 | 1.33279 | 1.74955 | 1.83775 | 1.84219 | 2.25895 | 2.34715 | 3.65445 | 4.0712 | 4.1594 | 3.0752 | 3.49195 | 3.58015 | 3.37501 | 3.79176 | 3.87996 | 3.12075 | 3.5375 | 3.6257 | 3.69772 | 4.11447 | 4.20267 |
| 104176\_at | D6Ertd245e | DNA segment, Chr 6, ERATO Doi 245, expressed | 269774 | 6 | 7 |  |  | 5.26529 | 5.23858 | 5.22913 | 5.49361 | 5.50292 | 5.36467 | 5.61692 | 5.77983 | 6.17557 | 5.6163 | 7.11958 | 7.09403 | 6.80817 | 6.6512 | 6.26656 | 6.98918 | 5.12894 | 5.19127 | 5.14212 | 0.13635 | 0.0740123 | 0.123164 | 0.109642 | 0.0473048 | 0.096456 | 0.100198 | 0.0378606 | 0.0870118 | 0.364674 | 0.302337 | 0.351488 | 0.37398 | 0.311643 | 0.360794 | 0.235731 | 0.173394 | 0.222545 | 0.48798 | 0.425643 | 0.474794 | 0.650895 | 0.588558 | 0.637709 | 1.04664 | 0.9843 | 1.03345 | 0.487362 | 0.425025 | 0.474176 | 1.99064 | 1.9283 | 1.97746 | 1.9651 | 1.90276 | 1.95191 | 1.67923 | 1.6169 | 1.66605 | 1.52226 | 1.45992 | 1.50907 | 1.13762 | 1.07528 | 1.12444 | 1.86025 | 1.79791 | 1.84706 |
| 104198\_at | E030027H19Rik | RIKEN cDNA E030027H19 gene | 109332 | 9 | 5 |  |  | 2.75092 | 2.82592 | 3.41661 | 2.77148 | 3.11628 | 2.83866 | 2.79431 | 2.83896 | 3.94118 | 5.29694 | 6.631 | 6.60652 | 5.2262 | 5.54905 | 7.3041 | 7.07018 | 4.25866 | 4.6593 | 3.56496 | -1.50774 | -1.90838 | -0.814044 | -1.43273 | -1.83337 | -0.739036 | -0.84205 | -1.24269 | -0.148351 | -1.48718 | -1.88782 | -0.793481 | -1.14238 | -1.54302 | -0.448681 | -1.42 | -1.82064 | -0.726301 | -1.46435 | -1.86498 | -0.770647 | -1.4197 | -1.82034 | -0.726001 | -0.317476 | -0.718114 | 0.376223 | 1.03829 | 0.637647 | 1.73198 | 2.37234 | 1.9717 | 3.06604 | 2.34786 | 1.94723 | 3.04156 | 0.96754 | 0.566902 | 1.66124 | 1.29039 | 0.889748 | 1.98409 | 3.04544 | 2.6448 | 3.73914 | 2.81152 | 2.41088 | 3.50522 |
| 104337\_f\_at | Pkp2 | plakophilin 2 | 67451 | 16 | 6 |  |  | 4.5861 | 4.02006 | 4.40409 | 6.06846 | 7.03973 | 5.40821 | 5.62264 | 4.68894 | 7.56741 | 7.78056 | 9.35888 | 8.90122 | 8.82905 | 8.48261 | 9.05785 | 8.3046 | 6.54837 | 7.5936 | 8.11586 | -1.96226 | -3.0075 | -3.52976 | -2.5283 | -3.57354 | -4.0958 | -2.14428 | -3.18951 | -3.71178 | -0.47991 | -1.52514 | -2.04741 | 0.491369 | -0.553865 | -1.07613 | -1.14015 | -2.18538 | -2.70765 | -0.925721 | -1.97095 | -2.49322 | -1.85942 | -2.90466 | -3.42692 | 1.01904 | -0.0261893 | -0.548453 | 1.23219 | 0.186958 | -0.335306 | 2.81051 | 1.76528 | 1.24301 | 2.35285 | 1.30762 | 0.785355 | 2.28068 | 1.23545 | 0.713187 | 1.93425 | 0.889014 | 0.366751 | 2.50949 | 1.46426 | 0.941992 | 1.75623 | 0.711001 | 0.188738 |
| 104467\_at |  |  | 103614 |  |  |  |  | 6.59753 | 6.95288 | 7.4465 | 6.34855 | 7.31851 | 7.60936 | 7.24188 | 7.26322 | 9.24791 | 9.7311 | 9.3707 | 9.75894 | 8.10571 | 10.5726 | 9.69052 | 9.92324 | 6.80396 | 5.67573 | 4.44797 | -0.206437 | 0.921798 | 2.14956 | 0.148919 | 1.27715 | 2.50491 | 0.64254 | 1.77077 | 2.99854 | -0.455414 | 0.672821 | 1.90058 | 0.514544 | 1.64278 | 2.87054 | 0.805403 | 1.93364 | 3.1614 | 0.437918 | 1.56615 | 2.79391 | 0.459259 | 1.58749 | 2.81526 | 2.44395 | 3.57218 | 4.79994 | 2.92713 | 4.05537 | 5.28313 | 2.56674 | 3.69497 | 4.92274 | 2.95498 | 4.08321 | 5.31098 | 1.30175 | 2.42998 | 3.65774 | 3.76865 | 4.89689 | 6.12465 | 2.88656 | 4.01479 | 5.24256 | 3.11927 | 4.24751 | 5.47527 |
| 104480\_at | Dsg2 | desmoglein 2 | 13511 | 18 | 8 | calcium ion binding  cell adhesion  cytoskeleton  desmosome  homophilic cell adhesion  integral to membrane  membrane  protein binding |  | 2.37323 | 2.5092 | 2.59241 | 4.07355 | 6.45226 | 2.5092 | 2.46882 | 2.51146 | 6.64327 | 7.35161 | 8.62487 | 8.85861 | 8.35377 | 9.34419 | 9.60747 | 8.64721 | 6.32016 | 6.57117 | 6.79374 | -3.94693 | -4.19795 | -4.42051 | -3.81096 | -4.06197 | -4.28453 | -3.72775 | -3.97877 | -4.20133 | -2.24661 | -2.49762 | -2.72018 | 0.132101 | -0.11891 | -0.341474 | -3.81096 | -4.06197 | -4.28454 | -3.85134 | -4.10235 | -4.32491 | -3.8087 | -4.05971 | -4.28227 | 0.323108 | 0.0720976 | -0.150467 | 1.03145 | 0.78044 | 0.557876 | 2.30471 | 2.0537 | 1.83114 | 2.53845 | 2.28744 | 2.06487 | 2.03361 | 1.7826 | 1.56003 | 3.02403 | 2.77302 | 2.55045 | 3.28731 | 3.0363 | 2.81373 | 2.32705 | 2.07604 | 1.85347 |
| 104483\_at | Col9a1 | procollagen, type IX, alpha 1 | 12839 | 1 | 10 | cell adhesion  collagen  cytoplasm  extracellular matrix  extracellular matrix structural constituent  extracellular matrix structural constituent conferring tensile strength  extracellular space  phosphate transport |  | 1.72466 | 2.01173 | 3.12389 | 1.71677 | 4.6478 | 2.91794 | 2.08321 | 2.42422 | 8.98849 | 9.82397 | 9.91093 | 10.2714 | 9.50743 | 10.7807 | 10.3754 | 10.8089 | 7.20697 | 6.78543 | 4.59281 | -5.48231 | -5.06077 | -2.86816 | -5.19524 | -4.7737 | -2.58109 | -4.08308 | -3.66154 | -1.46892 | -5.4902 | -5.06866 | -2.87605 | -2.55917 | -2.13763 | 0.0549821 | -4.28903 | -3.86749 | -1.67488 | -5.12376 | -4.70222 | -2.5096 | -4.78275 | -4.36121 | -2.1686 | 1.78153 | 2.20307 | 4.39568 | 2.617 | 3.03854 | 5.23115 | 2.70396 | 3.1255 | 5.31812 | 3.0644 | 3.48594 | 5.67855 | 2.30046 | 2.722 | 4.91461 | 3.57377 | 3.99532 | 6.18793 | 3.16845 | 3.59 | 5.78261 | 3.60196 | 4.0235 | 6.21611 |
| 104574\_at | 5730453I16Rik | RIKEN cDNA 5730453I16 gene | 269061 | 19 | 7 | nucleic acid binding |  | 3.96499 | 4.32334 | 3.92392 | 4.23716 | 4.30141 | 3.92519 | 3.64259 | 4.36783 | 4.80432 | 6.10556 | 6.48341 | 6.58594 | 6.08457 | 5.90196 | 6.67957 | 6.4448 | 3.69452 | 3.27704 | 3.40485 | 0.27047 | 0.687944 | 0.560143 | 0.628821 | 1.0463 | 0.918494 | 0.229401 | 0.646875 | 0.519074 | 0.54264 | 0.960114 | 0.832313 | 0.606888 | 1.02436 | 0.896561 | 0.230671 | 0.648145 | 0.520344 | -0.0519308 | 0.365544 | 0.237743 | 0.673311 | 1.09079 | 0.962985 | 1.1098 | 1.52727 | 1.39947 | 2.41104 | 2.82852 | 2.70072 | 2.78889 | 3.20637 | 3.07857 | 2.89142 | 3.30889 | 3.18109 | 2.39005 | 2.80752 | 2.67972 | 2.20744 | 2.62492 | 2.49711 | 2.98506 | 3.40253 | 3.27473 | 2.75028 | 3.16776 | 3.03996 |
| 104639\_i\_at | 4930553M18Rik | RIKEN cDNA 4930553M18 gene | 75316 | 9 | 5 |  |  | 4.9509 | 4.27125 | 3.24772 | 4.96281 | 4.9473 | 4.06807 | 5.03999 | 4.96971 | 5.13222 | 6.39165 | 6.19795 | 6.5583 | 6.0727 | 6.51024 | 7.557 | 7.04198 | 4.5429 | 4.52986 | 2.08719 | 0.408004 | 0.421037 | 2.86371 | -0.271642 | -0.258609 | 2.18407 | -1.29518 | -1.28214 | 1.16053 | 0.419913 | 0.432946 | 2.87562 | 0.404409 | 0.417442 | 2.86012 | -0.474825 | -0.461792 | 1.98088 | 0.497094 | 0.510127 | 2.9528 | 0.426814 | 0.439847 | 2.88252 | 0.589324 | 0.602357 | 3.04503 | 1.84875 | 1.86178 | 4.30446 | 1.65505 | 1.66808 | 4.11076 | 2.01541 | 2.02844 | 4.47111 | 1.52981 | 1.54284 | 3.98552 | 1.96734 | 1.98038 | 4.42305 | 3.01411 | 3.02714 | 5.46981 | 2.49908 | 2.51212 | 4.95479 |
| 104640\_f\_at | 4930553M18Rik | RIKEN cDNA 4930553M18 gene | 75316 | 9 | 5 |  |  | 6.94294 | 6.17261 | 6.96123 | 7.12025 | 6.99052 | 6.35358 | 7.83826 | 6.61846 | 8.4446 | 8.27361 | 8.87714 | 8.31896 | 8.38386 | 8.7982 | 9.55819 | 9.16274 | 6.42632 | 7.06879 | 3.74515 | 0.516627 | -0.125849 | 3.19779 | -0.25371 | -0.896186 | 2.42746 | 0.534911 | -0.107564 | 3.21608 | 0.69393 | 0.0514549 | 3.3751 | 0.564201 | -0.0782746 | 3.24537 | -0.0727349 | -0.71521 | 2.60843 | 1.41194 | 0.769467 | 4.09311 | 0.192139 | -0.450337 | 2.87331 | 2.01829 | 1.37581 | 4.69945 | 1.84729 | 1.20482 | 4.52846 | 2.45082 | 1.80835 | 5.13199 | 1.89265 | 1.25017 | 4.57381 | 1.95755 | 1.31507 | 4.63871 | 2.37188 | 1.72941 | 5.05305 | 3.13188 | 2.4894 | 5.81304 | 2.73642 | 2.09394 | 5.41758 |
| 104643\_at | BC037006 | cDNA sequence BC037006 | 211652 | 11 | 1 |  |  | 2.97987 | 3.05405 | 3.63135 | 3.71505 | 6.93451 | 3.27574 | 3.93676 | 3.12268 | 9.18896 | 8.87286 | 9.2341 | 9.75188 | 9.5482 | 10.0703 | 10.6647 | 11.3003 | 6.47663 | 6.00712 | 4.9483 | -3.49676 | -3.02724 | -1.96843 | -3.42258 | -2.95307 | -1.89425 | -2.84528 | -2.37576 | -1.31695 | -2.76158 | -2.29206 | -1.23325 | 0.457879 | 0.927391 | 1.98621 | -3.20089 | -2.73138 | -1.67256 | -2.53987 | -2.07036 | -1.01154 | -3.35395 | -2.88444 | -1.82562 | 2.71233 | 3.18184 | 4.24066 | 2.39623 | 2.86575 | 3.92456 | 2.75747 | 3.22699 | 4.2858 | 3.27525 | 3.74476 | 4.80358 | 3.07157 | 3.54108 | 4.5999 | 3.59366 | 4.06317 | 5.12199 | 4.18803 | 4.65754 | 5.71636 | 4.82363 | 5.29314 | 6.35196 |
| 104714\_at | Dock9 | dedicator of cytokinesis 9 | 105445 | 14 | 7 | GTP binding  GTPase binding  guanyl-nucleotide exchange factor activity  membrane |  | 7.55356 | 6.65249 | 7.89542 | 8.4486 | 7.74338 | 8.31395 | 8.85916 | 8.80854 | 9.60743 | 9.55401 | 10.0197 | 9.94385 | 9.77845 | 9.9588 | 9.88953 | 10.0072 | 6.31143 | 7.00965 | 6.76707 | 1.24213 | 0.543915 | 0.786494 | 0.341063 | -0.357153 | -0.114573 | 1.58399 | 0.885771 | 1.12835 | 2.13717 | 1.43895 | 1.68153 | 1.43195 | 0.733732 | 0.976312 | 2.00252 | 1.30431 | 1.54689 | 2.54773 | 1.84952 | 2.09209 | 2.49711 | 1.7989 | 2.04148 | 3.296 | 2.59779 | 2.84037 | 3.24258 | 2.54437 | 2.78695 | 3.70827 | 3.01006 | 3.25264 | 3.63242 | 2.9342 | 3.17678 | 3.46702 | 2.7688 | 3.01138 | 3.64737 | 2.94916 | 3.19174 | 3.5781 | 2.87988 | 3.12246 | 3.69572 | 2.99751 | 3.24009 |
| 104716\_at | Rbp1 | retinol binding protein 1, cellular | 19659 | 9 | 8 | binding  lipid binding  retinoid binding  retinoid metabolism  retinol binding  transport  transporter activity |  | 6.67871 | 7.20136 | 8.25322 | 8.36486 | 10.0382 | 8.59255 | 9.99309 | 8.74716 | 12.0854 | 11.8877 | 12.2951 | 12.5647 | 12.5479 | 12.5112 | 12.9715 | 12.7516 | 11.1112 | 10.9518 | 10.9797 | -4.4325 | -4.27307 | -4.301 | -3.90985 | -3.75041 | -3.77835 | -2.85799 | -2.69856 | -2.72649 | -2.74635 | -2.58692 | -2.61485 | -1.07302 | -0.913586 | -0.94152 | -2.51866 | -2.35923 | -2.38716 | -1.11812 | -0.958688 | -0.986622 | -2.36405 | -2.20462 | -2.23255 | 0.974201 | 1.13363 | 1.1057 | 0.776519 | 0.935951 | 0.908017 | 1.18385 | 1.34329 | 1.31535 | 1.45353 | 1.61296 | 1.58503 | 1.43673 | 1.59616 | 1.56823 | 1.39997 | 1.5594 | 1.53146 | 1.86032 | 2.01976 | 1.99182 | 1.64038 | 1.79981 | 1.77188 |
| 104725\_at | Rhoq | ras homolog gene family, member Q | 104215 | 17 | 8 | GTP binding  GTPase activity  GTPase activity  actin cytoskeleton organization and biogenesis  plasma membrane  protein binding  regulation of cell shape  small GTPase mediated signal transduction |  | 8.70817 | 8.56699 | 9.18828 | 8.85869 | 9.26284 | 9.1978 | 9.28724 | 9.07038 | 9.56982 | 9.33771 | 10.1373 | 10.2471 | 9.95093 | 10.0831 | 10.4352 | 10.2664 | 8.10899 | 8.82524 | 8.48394 | 0.59918 | -0.117074 | 0.224229 | 0.457999 | -0.258254 | 0.0830483 | 1.07929 | 0.363036 | 0.704339 | 0.749704 | 0.03345 | 0.374753 | 1.15385 | 0.437597 | 0.778899 | 1.08882 | 0.372563 | 0.713865 | 1.17825 | 0.461997 | 0.803299 | 0.961394 | 0.24514 | 0.586443 | 1.46083 | 0.744575 | 1.08588 | 1.22873 | 0.512474 | 0.853776 | 2.02826 | 1.31201 | 1.65331 | 2.13815 | 1.4219 | 1.7632 | 1.84194 | 1.12569 | 1.46699 | 1.97412 | 1.25787 | 1.59917 | 2.32618 | 1.60992 | 1.95123 | 2.15742 | 1.44117 | 1.78247 |
| 160106\_at | Capg | capping protein (actin filament), gelsolin-like | 12332 | 6 | 2 | actin binding  nucleus |  | 7.70141 | 8.36697 | 8.34293 | 8.25121 | 8.32762 | 8.20468 | 8.29334 | 7.86627 | 9.67738 | 9.49172 | 9.4702 | 9.96303 | 10.3196 | 9.90293 | 10.3802 | 10.5287 | 7.05148 | 7.3309 | 7.72782 | 0.649929 | 0.370506 | -0.0264099 | 1.3155 | 1.03607 | 0.639156 | 1.29145 | 1.01202 | 0.615109 | 1.19973 | 0.920305 | 0.523389 | 1.27614 | 0.996719 | 0.599803 | 1.1532 | 0.873773 | 0.476857 | 1.24187 | 0.962441 | 0.565526 | 0.814794 | 0.53537 | 0.138455 | 2.6259 | 2.34648 | 1.94956 | 2.44024 | 2.16082 | 1.7639 | 2.41872 | 2.1393 | 1.74239 | 2.91155 | 2.63213 | 2.23521 | 3.26812 | 2.9887 | 2.59178 | 2.85145 | 2.57203 | 2.17511 | 3.32876 | 3.04934 | 2.65242 | 3.47725 | 3.19783 | 2.80092 |
| 160119\_at | Mglap | matrix gamma-carboxyglutamate (gla) protein | 17313 | 6 | 9 | calcium ion binding  extracellular  extracellular space  regulation of bone mineralization |  | 3.36789 | 3.95146 | 3.9655 | 3.93524 | 4.491 | 3.76153 | 3.96116 | 3.95953 | 5.21197 | 4.99366 | 4.82118 | 4.97552 | 5.01022 | 5.6297 | 5.46911 | 5.76029 | 4.96185 | 5.14719 | 5.85098 | -1.59396 | -1.7793 | -2.48309 | -1.01039 | -1.19573 | -1.89952 | -0.996351 | -1.18169 | -1.88548 | -1.0266 | -1.21194 | -1.91573 | -0.47085 | -0.656187 | -1.35998 | -1.20032 | -1.38565 | -2.08944 | -1.00069 | -1.18603 | -1.88982 | -1.00232 | -1.18765 | -1.89144 | 0.250121 | 0.064785 | -0.639005 | 0.031808 | -0.153528 | -0.857318 | -0.140669 | -0.326006 | -1.0298 | 0.0136716 | -0.171665 | -0.875455 | 0.0483711 | -0.136965 | -0.840755 | 0.667853 | 0.482516 | -0.221274 | 0.507261 | 0.321925 | -0.381865 | 0.798439 | 0.613103 | -0.0906869 |
| 160249\_at | Tpd52 | tumor protein D52 | 21985 | 3 | 7 |  |  | 3.18109 | 4.20675 | 4.307 | 4.15551 | 5.08349 | 4.05292 | 4.20366 | 3.87151 | 5.21691 | 5.3419 | 6.15599 | 5.72143 | 5.93312 | 5.82792 | 6.49026 | 6.49741 | 5.86748 | 6.62843 | 6.20719 | -2.68639 | -3.44733 | -3.02609 | -1.66073 | -2.42168 | -2.00043 | -1.56048 | -2.32142 | -1.90018 | -1.71197 | -2.47291 | -2.05167 | -0.783994 | -1.54494 | -1.12369 | -1.81456 | -2.57551 | -2.15426 | -1.66382 | -2.42476 | -2.00352 | -1.99597 | -2.75691 | -2.33567 | -0.650571 | -1.41151 | -0.990272 | -0.52558 | -1.28652 | -0.865281 | 0.288508 | -0.472434 | -0.0511931 | -0.146055 | -0.906997 | -0.485755 | 0.0656386 | -0.695303 | -0.274062 | -0.0395641 | -0.800506 | -0.379265 | 0.622774 | -0.138168 | 0.283074 | 0.629921 | -0.131021 | 0.290221 |
| 160253\_at | Ifitm3 | interferon induced transmembrane protein 3 | 66141 | 7 | 9 | cytoplasmic vesicle  immune response  integral to membrane  negative regulation of cell proliferation |  | 10.6389 | 10.7376 | 10.85 | 10.7275 | 11.3358 | 10.6264 | 11.289 | 10.523 | 11.1665 | 11.9995 | 12.1653 | 12.3871 | 12.4929 | 12.0149 | 12.7955 | 12.6512 | 9.937 | 9.84893 | 10.5102 | 0.701883 | 0.78995 | 0.128662 | 0.800573 | 0.888639 | 0.227351 | 0.913029 | 1.0011 | 0.339808 | 0.790506 | 0.878573 | 0.217285 | 1.39876 | 1.48682 | 0.825536 | 0.689442 | 0.777508 | 0.11622 | 1.35202 | 1.44008 | 0.778795 | 0.586003 | 0.67407 | 0.0127819 | 1.22952 | 1.31758 | 0.656294 | 2.06248 | 2.15055 | 1.48926 | 2.22828 | 2.31635 | 1.65506 | 2.45013 | 2.5382 | 1.87691 | 2.55595 | 2.64402 | 1.98273 | 2.07794 | 2.166 | 1.50472 | 2.85848 | 2.94655 | 2.28526 | 2.71417 | 2.80224 | 2.14095 |
| 160319\_at | Sparcl1 | SPARC-like 1 (mast9, hevin) | 13602 | 5 | 9 | calcium ion binding  extracellular matrix  extracellular space |  | 9.2124 | 9.32352 | 9.2402 | 9.72788 | 9.87149 | 9.91803 | 10.1427 | 10.1294 | 11.182 | 10.4767 | 11.6271 | 11.2177 | 11.1044 | 11.6005 | 11.0827 | 11.695 | 9.55174 | 9.63826 | 8.9985 | -0.339339 | -0.425856 | 0.213897 | -0.228222 | -0.314739 | 0.325015 | -0.311542 | -0.398059 | 0.241694 | 0.176146 | 0.0896289 | 0.729382 | 0.319754 | 0.233237 | 0.87299 | 0.366288 | 0.279771 | 0.919525 | 0.591001 | 0.504484 | 1.14424 | 0.577637 | 0.49112 | 1.13087 | 1.6303 | 1.54378 | 2.18353 | 0.924966 | 0.838449 | 1.4782 | 2.07538 | 1.98886 | 2.62862 | 1.66592 | 1.5794 | 2.21915 | 1.55263 | 1.46611 | 2.10587 | 2.04874 | 1.96223 | 2.60198 | 1.53099 | 1.44447 | 2.08423 | 2.14327 | 2.05675 | 2.69651 |
| 160335\_at | Gclm | glutamate-cysteine ligase , modifier subunit | 14630 | 3 | 9 | glutamate-cysteine ligase activity  glutathione biosynthesis  ligase activity | Glutamate metabolism  Glutathione metabolism | 5.78567 | 5.26876 | 6.2741 | 5.69649 | 5.78691 | 5.91234 | 5.75295 | 6.23455 | 6.63294 | 6.16688 | 7.36692 | 6.77729 | 6.9646 | 6.86351 | 7.91256 | 7.35696 | 6.2414 | 6.09339 | 5.61845 | -0.455732 | -0.307726 | 0.167217 | -0.972642 | -0.824636 | -0.349693 | 0.0327047 | 0.180711 | 0.655654 | -0.544915 | -0.396908 | 0.0780347 | -0.454487 | -0.306481 | 0.168462 | -0.329056 | -0.18105 | 0.293894 | -0.488454 | -0.340447 | 0.134496 | -0.00685251 | 0.141154 | 0.616097 | 0.391535 | 0.539541 | 1.01448 | -0.0745203 | 0.0734859 | 0.548429 | 1.12552 | 1.27353 | 1.74847 | 0.535888 | 0.683894 | 1.15884 | 0.723203 | 0.871209 | 1.34615 | 0.622107 | 0.770113 | 1.24506 | 1.67116 | 1.81917 | 2.29411 | 1.11556 | 1.26357 | 1.73851 |
| 160393\_at | Etnk1 | ethanolamine kinase 1 | 75320 | 6 | 6 | kinase activity  transferase activity |  | 8.20815 | 7.86055 | 8.68338 | 8.38564 | 9.48486 | 8.85975 | 9.12874 | 8.95583 | 9.93835 | 10.1667 | 10.7144 | 10.7469 | 10.0963 | 11.2254 | 9.91514 | 10.7693 | 4.27273 | 5.24674 | 4.08302 | 3.93542 | 2.96141 | 4.12513 | 3.58782 | 2.61382 | 3.77753 | 4.41065 | 3.43664 | 4.60036 | 4.11291 | 3.13891 | 4.30262 | 5.21213 | 4.23812 | 5.40184 | 4.58702 | 3.61301 | 4.77673 | 4.85602 | 3.88201 | 5.04572 | 4.6831 | 3.70909 | 4.87281 | 5.66562 | 4.69161 | 5.85533 | 5.89394 | 4.91994 | 6.08365 | 6.44163 | 5.46763 | 6.63134 | 6.4742 | 5.50019 | 6.66391 | 5.82354 | 4.84953 | 6.01325 | 6.95269 | 5.97868 | 7.1424 | 5.64242 | 4.66841 | 5.83212 | 6.49662 | 5.52261 | 6.68633 |
| 160444\_at | 2010319C14Rik | RIKEN cDNA 2010319C14 gene | 72084 | 16 | 6 |  |  | 5.28955 | 5.4491 | 5.62165 | 5.16694 | 5.65828 | 5.39363 | 5.73023 | 5.72223 | 6.36059 | 6.62554 | 7.5035 | 6.87793 | 6.73934 | 6.95641 | 7.51681 | 7.01837 | 5.14625 | 5.39844 | 4.85171 | 0.143302 | -0.10889 | 0.437841 | 0.302846 | 0.0506547 | 0.597385 | 0.475394 | 0.223203 | 0.769934 | 0.0206894 | -0.231502 | 0.315229 | 0.512029 | 0.259837 | 0.806568 | 0.247375 | -0.00481632 | 0.541914 | 0.583975 | 0.331784 | 0.878514 | 0.575974 | 0.323783 | 0.870514 | 1.21434 | 0.962152 | 1.50888 | 1.47929 | 1.2271 | 1.77383 | 2.35725 | 2.10506 | 2.65179 | 1.73168 | 1.47948 | 2.02622 | 1.59309 | 1.3409 | 1.88763 | 1.81016 | 1.55797 | 2.1047 | 2.37056 | 2.11837 | 2.6651 | 1.87212 | 1.61993 | 2.16666 |
| 160469\_at | Thbs1 | thrombospondin 1 | 21825 | 2 | 19 | calcium ion binding  cell adhesion  extracellular  extracellular space  extracellular space  heparin binding  negative regulation of angiogenesis  protein binding  structural molecule activity | TGF-beta signaling pathway | 4.88615 | 7.12306 | 8.87874 | 8.3363 | 9.76809 | 8.92725 | 8.9466 | 8.99569 | 10.6762 | 10.8216 | 11.4286 | 11.4489 | 11.0651 | 11.8791 | 10.944 | 11.6623 | 6.98814 | 6.68013 | 6.82739 | -2.102 | -1.79399 | -1.94124 | 0.134911 | 0.442922 | 0.29567 | 1.8906 | 2.19861 | 2.05135 | 1.34816 | 1.65617 | 1.50892 | 2.77994 | 3.08796 | 2.9407 | 1.93911 | 2.24712 | 2.09986 | 1.95845 | 2.26647 | 2.11921 | 2.00755 | 2.31556 | 2.1683 | 3.6881 | 3.99611 | 3.84886 | 3.83347 | 4.14148 | 3.99423 | 4.44041 | 4.74842 | 4.60117 | 4.46079 | 4.76881 | 4.62155 | 4.07691 | 4.38492 | 4.23767 | 4.89096 | 5.19897 | 5.05172 | 3.95584 | 4.26385 | 4.1166 | 4.67417 | 4.98218 | 4.83493 |
| 160520\_at | Yap1 | yes-associated protein 1, 65kDa | 22601 | 9 | 3 | biological\_process unknown  cellular\_component unknown  molecular\_function unknown |  | 7.21374 | 7.26763 | 7.92552 | 7.32836 | 7.74743 | 7.7363 | 7.85862 | 7.45264 | 8.53838 | 8.92247 | 8.46616 | 8.89301 | 8.51605 | 9.31361 | 9.00688 | 9.09748 | 8.00584 | 8.1162 | 7.87989 | -0.792093 | -0.902453 | -0.666142 | -0.738203 | -0.848563 | -0.612253 | -0.080319 | -0.190679 | 0.0456314 | -0.677481 | -0.787841 | -0.551531 | -0.258408 | -0.368768 | -0.132458 | -0.269538 | -0.379898 | -0.143588 | -0.147213 | -0.257573 | -0.0212628 | -0.553195 | -0.663555 | -0.427244 | 0.53254 | 0.42218 | 0.658491 | 0.916631 | 0.806271 | 1.04258 | 0.460326 | 0.349965 | 0.586276 | 0.887172 | 0.776812 | 1.01312 | 0.510214 | 0.399854 | 0.636164 | 1.30778 | 1.19742 | 1.43373 | 1.00104 | 0.890683 | 1.12699 | 1.09165 | 0.981285 | 1.2176 |
| 160546\_at | Aldoc | aldolase 3, C isoform | 11676 | 11 | 12 | fructose-bisphosphate aldolase activity  glycolysis  lyase activity  mitochondrion |  | 3.96647 | 4.48682 | 6.65786 | 8.26573 | 10.7453 | 8.10572 | 9.98022 | 8.66734 | 12.0527 | 11.5811 | 12.8198 | 12.8602 | 12.967 | 12.1216 | 12.6074 | 12.5965 | 9.66607 | 9.51691 | 9.77767 | -5.69961 | -5.55044 | -5.8112 | -5.17925 | -5.03009 | -5.29085 | -3.00821 | -2.85905 | -3.11981 | -1.40035 | -1.25118 | -1.51194 | 1.07924 | 1.22841 | 0.967649 | -1.56036 | -1.41119 | -1.67195 | 0.314141 | 0.463306 | 0.202546 | -0.998736 | -0.849572 | -1.11033 | 2.38663 | 2.5358 | 2.27504 | 1.91498 | 2.06415 | 1.80339 | 3.15372 | 3.30289 | 3.04213 | 3.19417 | 3.34333 | 3.08257 | 3.30088 | 3.45004 | 3.18928 | 2.4555 | 2.60467 | 2.34391 | 2.9413 | 3.09046 | 2.8297 | 2.93044 | 3.0796 | 2.81884 |
| 160564\_at | Lcn2 | lipocalin 2 | 16819 | 2 | 12 | binding  extracellular space  transport  transporter activity |  | 9.66411 | 9.95129 | 11.5524 | 12.5238 | 13.7303 | 12.172 | 13.1004 | 12.5773 | 14.1531 | 13.5992 | 14.537 | 14.4984 | 14.5736 | 14.4191 | 14.4227 | 14.4892 | 12.2169 | 12.4917 | 12.3369 | -2.55279 | -2.82759 | -2.6728 | -2.26562 | -2.54042 | -2.38562 | -0.664537 | -0.939338 | -0.784538 | 0.30689 | 0.0320894 | 0.186889 | 1.51341 | 1.23861 | 1.39341 | -0.044934 | -0.319734 | -0.164935 | 0.883449 | 0.608648 | 0.763448 | 0.360418 | 0.0856182 | 0.240417 | 1.93621 | 1.66141 | 1.81621 | 1.38232 | 1.10752 | 1.26232 | 2.32013 | 2.04533 | 2.20013 | 2.28151 | 2.00671 | 2.16151 | 2.35668 | 2.08188 | 2.23668 | 2.20223 | 1.92743 | 2.08223 | 2.2058 | 1.931 | 2.0858 | 2.27231 | 1.99751 | 2.15231 |
| 160565\_at | Ckmt1 | creatine kinase, mitochondrial 1, ubiquitous | 12716 | 2 | 8 | creatine kinase activity  extracellular space  kinase activity  mitochondrion  mitochondrion  transferase activity  transferase activity, transferring phosphorus-containing groups | Urea cycle and metabolism of amino groups  Arginine and proline metabolism | 2.95841 | 2.96554 | 3.0687 | 3.06327 | 6.38989 | 3.03684 | 4.65908 | 3.03625 | 7.62925 | 7.32968 | 9.7765 | 9.81069 | 10.2293 | 8.59858 | 9.57232 | 9.17627 | 5.05816 | 5.87283 | 6.27282 | -2.09976 | -2.91442 | -3.31441 | -2.09263 | -2.9073 | -3.30729 | -1.98946 | -2.80413 | -3.20412 | -1.99489 | -2.80956 | -3.20955 | 1.33173 | 0.517062 | 0.117072 | -2.02132 | -2.83599 | -3.23598 | -0.399085 | -1.21375 | -1.61374 | -2.02191 | -2.83658 | -3.23657 | 2.57109 | 1.75642 | 1.35643 | 2.27152 | 1.45685 | 1.05686 | 4.71833 | 3.90367 | 3.50368 | 4.75252 | 3.93785 | 3.53786 | 5.17118 | 4.35651 | 3.95652 | 3.54041 | 2.72574 | 2.32575 | 4.51416 | 3.69949 | 3.2995 | 4.11811 | 3.30344 | 2.90345 |
| 160622\_at | BC011209 | cDNA sequence BC011209 | 217721 | 12 | 5 | integral to membrane  transport |  | 2.96968 | 3.0399 | 3.09424 | 2.97316 | 3.00837 | 3.06716 | 3.03507 | 3.06651 | 3.17466 | 5.78674 | 7.61677 | 6.53767 | 7.20931 | 7.09109 | 7.76808 | 7.62871 | 2.9439 | 2.94411 | 2.90215 | 0.0257795 | 0.0255686 | 0.067531 | 0.0959993 | 0.0957885 | 0.137751 | 0.150344 | 0.150133 | 0.192095 | 0.0292625 | 0.0290516 | 0.071014 | 0.0644681 | 0.0642572 | 0.10622 | 0.123262 | 0.123052 | 0.165014 | 0.0911691 | 0.0909582 | 0.132921 | 0.122609 | 0.122398 | 0.164361 | 0.230761 | 0.23055 | 0.272512 | 2.84284 | 2.84263 | 2.88459 | 4.67287 | 4.67266 | 4.71462 | 3.59377 | 3.59356 | 3.63552 | 4.26541 | 4.2652 | 4.30716 | 4.14719 | 4.14698 | 4.18894 | 4.82418 | 4.82397 | 4.86593 | 4.68481 | 4.6846 | 4.72656 |
| 160688\_at | Golph3 | golgi phosphoprotein 3 | 66629 | 15 | 8 | Golgi apparatus  membrane |  | 8.43011 | 8.01155 | 8.29438 | 8.31769 | 9.44634 | 8.47684 | 8.98796 | 8.35806 | 9.50803 | 9.50273 | 10.5469 | 10.224 | 9.78437 | 10.2791 | 10.2374 | 9.85008 | 10.2058 | 10.4648 | 10.4193 | -1.77565 | -2.03471 | -1.98921 | -2.19421 | -2.45327 | -2.40777 | -1.91138 | -2.17044 | -2.12494 | -1.88807 | -2.14713 | -2.10162 | -0.75942 | -1.01848 | -0.972974 | -1.72892 | -1.98798 | -1.94248 | -1.2178 | -1.47686 | -1.43135 | -1.8477 | -2.10676 | -2.06125 | -0.697729 | -0.956787 | -0.911283 | -0.703031 | -0.962089 | -0.916585 | 0.341148 | 0.0820906 | 0.127594 | 0.0182208 | -0.240837 | -0.195334 | -0.421389 | -0.680446 | -0.634943 | 0.073373 | -0.185685 | -0.140181 | 0.0315931 | -0.227465 | -0.181961 | -0.355683 | -0.61474 | -0.569237 |
| 160693\_at | Pip5k2c | phosphatidylinositol-4-phosphate 5-kinase, type II, gamma | 117150 | 10 | 3 | 1-phosphatidylinositol-4-phosphate 5-kinase activity  kinase activity |  | 3.50743 | 3.41568 | 3.80947 | 3.39019 | 3.48475 | 3.80064 | 3.75584 | 3.77667 | 3.87734 | 3.96534 | 4.72923 | 5.01563 | 5.14681 | 5.35134 | 5.77325 | 6.12237 | 3.63715 | 3.65859 | 3.51483 | -0.129721 | -0.151161 | -0.00739871 | -0.22147 | -0.24291 | -0.0991475 | 0.172325 | 0.150885 | 0.294647 | -0.246955 | -0.268395 | -0.124633 | -0.152395 | -0.173834 | -0.0300719 | 0.163487 | 0.142048 | 0.28581 | 0.118693 | 0.0972541 | 0.241016 | 0.13952 | 0.118081 | 0.261843 | 0.240195 | 0.218755 | 0.362517 | 0.328191 | 0.306752 | 0.450514 | 1.09208 | 1.07065 | 1.21441 | 1.37848 | 1.35704 | 1.5008 | 1.50966 | 1.48822 | 1.63198 | 1.71419 | 1.69275 | 1.83651 | 2.1361 | 2.11466 | 2.25843 | 2.48522 | 2.46378 | 2.60754 |
| 160775\_at | Tulp4 | tubby like protein 4 | 68842 | 17 | 6 | cytoplasm  intracellular signaling cascade  molecular\_function unknown |  | 7.68328 | 8.02908 | 8.22075 | 8.25809 | 8.55624 | 8.13089 | 8.13344 | 8.1736 | 8.43796 | 9.06457 | 9.36742 | 8.95311 | 8.93106 | 9.25247 | 9.34998 | 9.42719 | 7.85545 | 8.12642 | 7.81013 | -0.172168 | -0.443136 | -0.12685 | 0.173628 | -0.0973399 | 0.218946 | 0.365305 | 0.0943372 | 0.410623 | 0.402643 | 0.131675 | 0.447961 | 0.700791 | 0.429823 | 0.746109 | 0.275442 | 0.00447429 | 0.32076 | 0.277993 | 0.00702525 | 0.323311 | 0.318151 | 0.0471838 | 0.363469 | 0.582507 | 0.311539 | 0.627825 | 1.20912 | 0.938156 | 1.25444 | 1.51197 | 1.241 | 1.55729 | 1.09766 | 0.826696 | 1.14298 | 1.07561 | 0.804645 | 1.12093 | 1.39702 | 1.12605 | 1.44234 | 1.49453 | 1.22357 | 1.53985 | 1.57174 | 1.30078 | 1.61706 |
| 160801\_at | 2310009N05Rik | RIKEN cDNA 2310009N05 gene | 66943 | 18 | 6 |  |  | 2.27415 | 2.34513 | 2.37334 | 2.3197 | 2.32909 | 2.34187 | 2.34644 | 2.33832 | 4.34838 | 5.26737 | 6.42361 | 7.3203 | 6.5317 | 6.46548 | 7.36257 | 6.94148 | 2.29129 | 2.29682 | 2.27965 | -0.0171349 | -0.0226651 | -0.00549892 | 0.0538452 | 0.048315 | 0.0654812 | 0.082049 | 0.0765187 | 0.093685 | 0.0284091 | 0.0228789 | 0.0400451 | 0.0378063 | 0.0322761 | 0.0494423 | 0.0505822 | 0.045052 | 0.0622182 | 0.055157 | 0.0496267 | 0.066793 | 0.0470343 | 0.0415041 | 0.0586703 | 2.05709 | 2.05156 | 2.06872 | 2.97608 | 2.97055 | 2.98772 | 4.13232 | 4.12679 | 4.14396 | 5.02901 | 5.02348 | 5.04065 | 4.24041 | 4.23488 | 4.25205 | 4.17419 | 4.16866 | 4.18583 | 5.07129 | 5.06576 | 5.08292 | 4.65019 | 4.64466 | 4.66182 |
| 160806\_at | Stk39 | serine/threonine kinase 39, STE20/SPS1 homolog (yeast) | 53416 | 2 | 9 | ATP binding  protein amino acid phosphorylation  protein kinase activity  protein serine/threonine kinase activity  protein-tyrosine kinase activity  transferase activity |  | 2.98595 | 3.06222 | 3.3276 | 2.84829 | 3.36256 | 3.30137 | 3.31691 | 3.42823 | 6.90409 | 7.74824 | 8.1601 | 7.7716 | 6.99571 | 8.74263 | 8.09464 | 8.10786 | 3.05955 | 2.8488 | 2.81188 | -0.0735923 | 0.137149 | 0.174071 | 0.00267393 | 0.213415 | 0.250337 | 0.26805 | 0.478792 | 0.515713 | -0.211251 | -0.00050899 | 0.0364128 | 0.303013 | 0.513755 | 0.550677 | 0.241824 | 0.452566 | 0.489488 | 0.25736 | 0.468101 | 0.505023 | 0.368682 | 0.579424 | 0.616346 | 3.84454 | 4.05529 | 4.09221 | 4.68869 | 4.89943 | 4.93636 | 5.10056 | 5.3113 | 5.34822 | 4.71206 | 4.9228 | 4.95972 | 3.93616 | 4.1469 | 4.18383 | 5.68308 | 5.89382 | 5.93074 | 5.0351 | 5.24584 | 5.28276 | 5.04832 | 5.25906 | 5.29598 |
| 160819\_at | Ndrg4 | N-myc downstream regulated gene 4 | 234593 | 8 | 5 |  |  | 2.7249 | 2.77573 | 2.81647 | 2.7102 | 2.7729 | 2.78602 | 3.36894 | 2.79281 | 7.95483 | 9.14498 | 10.2299 | 10.7247 | 9.82565 | 10.2057 | 11.1375 | 9.98577 | 2.68876 | 2.69714 | 2.66371 | 0.0361426 | 0.027756 | 0.0611864 | 0.0869726 | 0.078586 | 0.112016 | 0.127716 | 0.11933 | 0.15276 | 0.0214382 | 0.0130516 | 0.046482 | 0.0841401 | 0.0757535 | 0.109184 | 0.097264 | 0.0888774 | 0.122308 | 0.680183 | 0.671796 | 0.705227 | 0.104056 | 0.0956699 | 0.1291 | 5.26607 | 5.25768 | 5.29111 | 6.45622 | 6.44784 | 6.48127 | 7.54118 | 7.53279 | 7.56622 | 8.03596 | 8.02757 | 8.061 | 7.13689 | 7.12851 | 7.16194 | 7.5169 | 7.50851 | 7.54195 | 8.44872 | 8.44033 | 8.47377 | 7.29701 | 7.28863 | 7.32206 |
| 160829\_at | Phlda1 | pleckstrin homology-like domain, family A, member 1 | 21664 | 10 | 3 | FasL biosynthesis |  | 8.59795 | 8.55317 | 10.0998 | 9.97603 | 11.7443 | 9.72294 | 10.3334 | 9.3859 | 11.8186 | 11.5676 | 12.6909 | 12.7479 | 12.3274 | 12.7481 | 12.4367 | 12.5859 | 11.6476 | 11.1928 | 11.4248 | -3.04967 | -2.59485 | -2.8269 | -3.09445 | -2.63963 | -2.87168 | -1.54786 | -1.09304 | -1.32509 | -1.67159 | -1.21677 | -1.44882 | 0.0966422 | 0.551461 | 0.319413 | -1.92468 | -1.46986 | -1.7019 | -1.31419 | -0.859374 | -1.09142 | -2.26172 | -1.8069 | -2.03895 | 0.170971 | 0.62579 | 0.393742 | -0.0800272 | 0.374792 | 0.142744 | 1.04324 | 1.49806 | 1.26601 | 1.10023 | 1.55505 | 1.323 | 0.679762 | 1.13458 | 0.902533 | 1.1005 | 1.55532 | 1.32327 | 0.789047 | 1.24387 | 1.01182 | 0.938239 | 1.39306 | 1.16101 |
| 160894\_at | Cebpd | CCAAT/enhancer binding protein (C/EBP), delta | 12609 | 16 | 15 | DNA binding  nucleus  protein homodimerization activity  regulation of transcription, DNA-dependent |  | 6.76355 | 7.60488 | 7.4464 | 8.17257 | 8.93977 | 7.85064 | 8.56586 | 8.38724 | 9.88232 | 9.14749 | 9.71921 | 10.1672 | 10.0633 | 10.4611 | 9.55284 | 10.2838 | 5.00615 | 6.17883 | 5.75856 | 1.75739 | 0.584716 | 1.00498 | 2.59872 | 1.42605 | 1.84631 | 2.44024 | 1.26757 | 1.68783 | 3.16642 | 1.99374 | 2.41401 | 3.93362 | 2.76094 | 3.18121 | 2.84449 | 1.67181 | 2.09208 | 3.55971 | 2.38703 | 2.8073 | 3.38109 | 2.20841 | 2.62868 | 4.87616 | 3.70349 | 4.12375 | 4.14134 | 2.96866 | 3.38893 | 4.71306 | 3.54038 | 3.96064 | 5.16108 | 3.98841 | 4.40867 | 5.05717 | 3.88449 | 4.30476 | 5.45493 | 4.28225 | 4.70252 | 4.54668 | 3.37401 | 3.79427 | 5.27764 | 4.10497 | 4.52523 |
| 160979\_at | Zranb1 | zinc finger, RAN-binding domain containing 1 | 360216 | 7 | 5 |  |  | 4.40615 | 5.63084 | 5.85991 | 4.48468 | 5.70905 | 5.30714 | 5.55189 | 4.91278 | 6.38723 | 8.28385 | 8.38475 | 9.03629 | 7.49517 | 8.75078 | 8.71413 | 8.4392 | 5.47771 | 5.38298 | 5.61981 | -1.07155 | -0.976829 | -1.21366 | 0.153135 | 0.247861 | 0.0110291 | 0.382206 | 0.476932 | 0.2401 | -0.993031 | -0.898306 | -1.13514 | 0.231347 | 0.326072 | 0.0892406 | -0.170569 | -0.0758437 | -0.312675 | 0.0741782 | 0.168904 | -0.067928 | -0.564928 | -0.470202 | -0.707034 | 0.909526 | 1.00425 | 0.767419 | 2.80615 | 2.90087 | 2.66404 | 2.90704 | 3.00177 | 2.76494 | 3.55858 | 3.65331 | 3.41648 | 2.01746 | 2.11218 | 1.87535 | 3.27307 | 3.3678 | 3.13097 | 3.23642 | 3.33115 | 3.09431 | 2.96149 | 3.05622 | 2.81939 |
| 161070\_at | Spred2 | sprouty protein with EVH-1 domain 2, related sequence | 114716 | 11 | 9 | inactivation of MAPK  plasma membrane  protein binding  stem cell factor receptor binding |  | 4.22087 | 4.2506 | 4.4481 | 4.16427 | 4.46075 | 4.36196 | 4.47053 | 4.34831 | 4.66191 | 4.88666 | 5.67539 | 5.99098 | 4.87604 | 6.35694 | 6.53985 | 5.84517 | 3.79792 | 3.79079 | 3.74178 | 0.422947 | 0.430078 | 0.47909 | 0.452683 | 0.459814 | 0.508826 | 0.650181 | 0.657312 | 0.706324 | 0.366354 | 0.373485 | 0.422497 | 0.662827 | 0.669958 | 0.71897 | 0.564039 | 0.57117 | 0.620182 | 0.672608 | 0.679738 | 0.728751 | 0.550389 | 0.55752 | 0.606532 | 0.863989 | 0.87112 | 0.920132 | 1.08874 | 1.09587 | 1.14488 | 1.87747 | 1.8846 | 1.93361 | 2.19306 | 2.20019 | 2.2492 | 1.07812 | 1.08525 | 1.13426 | 2.55902 | 2.56615 | 2.61516 | 2.74193 | 2.74906 | 2.79807 | 2.04725 | 2.05438 | 2.10339 |
| 161075\_at | D9Ertd280e | DNA segment, Chr 9, ERATO Doi 280, expressed | 272636 | 9 | 4 |  |  | 2.51333 | 2.58435 | 2.62756 | 2.51374 | 2.54437 | 2.59419 | 2.57062 | 2.58961 | 4.3523 | 4.71311 | 3.88516 | 5.35663 | 4.91302 | 4.29603 | 6.61596 | 5.81423 | 2.47773 | 2.49063 | 2.44391 | 0.0355952 | 0.0227026 | 0.069419 | 0.106617 | 0.0937244 | 0.140441 | 0.149821 | 0.136929 | 0.183645 | 0.0360002 | 0.0231076 | 0.069824 | 0.0666311 | 0.0537385 | 0.100455 | 0.116457 | 0.103565 | 0.150281 | 0.0928888 | 0.0799962 | 0.126713 | 0.111879 | 0.0989866 | 0.145703 | 1.87457 | 1.86167 | 1.90839 | 2.23537 | 2.22248 | 2.26919 | 1.40743 | 1.39454 | 1.44125 | 2.87889 | 2.866 | 2.91271 | 2.43529 | 2.4224 | 2.46911 | 1.81829 | 1.8054 | 1.85212 | 4.13823 | 4.12533 | 4.17205 | 3.3365 | 3.32361 | 3.37032 |
| 161825\_f\_at | Ceacam10 | CEA-related cell adhesion molecule 10 | 26366 | 7 | 8 | extracellular space |  | 3.28447 | 3.36093 | 3.40915 | 3.28579 | 3.95687 | 3.38066 | 3.33751 | 3.37257 | 5.23811 | 7.37319 | 9.05838 | 9.44831 | 9.05512 | 8.55901 | 9.83339 | 9.74897 | 3.2642 | 3.34727 | 3.22126 | 0.0202749 | -0.0627983 | 0.0632085 | 0.0967352 | 0.013662 | 0.139669 | 0.144951 | 0.061878 | 0.187885 | 0.0215923 | -0.0614809 | 0.0645259 | 0.692678 | 0.609604 | 0.735611 | 0.11646 | 0.0333865 | 0.159393 | 0.0733083 | -0.00976493 | 0.116242 | 0.108373 | 0.0252998 | 0.151307 | 1.97391 | 1.89084 | 2.01684 | 4.10899 | 4.02592 | 4.15193 | 5.79418 | 5.71111 | 5.83711 | 6.18412 | 6.10104 | 6.22705 | 5.79092 | 5.70785 | 5.83386 | 5.29481 | 5.21174 | 5.33775 | 6.56919 | 6.48612 | 6.61213 | 6.48477 | 6.4017 | 6.52771 |
| 162057\_f\_at |  |  | 52816 |  |  |  |  | 8.26602 | 8.3391 | 8.47683 | 7.72184 | 7.90382 | 8.5819 | 8.51121 | 8.59899 | 9.07667 | 8.70167 | 9.19041 | 9.31615 | 8.75208 | 9.45463 | 10.1191 | 9.71975 | 8.44093 | 8.4745 | 7.70223 | -0.174912 | -0.208483 | 0.563789 | -0.101833 | -0.135403 | 0.636868 | 0.0358975 | 0.00232713 | 0.774598 | -0.719091 | -0.752661 | 0.0196102 | -0.537114 | -0.570685 | 0.201586 | 0.140966 | 0.107395 | 0.879666 | 0.0702809 | 0.0367105 | 0.808982 | 0.15806 | 0.12449 | 0.896761 | 0.635741 | 0.602171 | 1.37444 | 0.260739 | 0.227168 | 0.99944 | 0.74948 | 0.71591 | 1.48818 | 0.875215 | 0.841645 | 1.61392 | 0.311151 | 0.27758 | 1.04985 | 1.0137 | 0.980131 | 1.7524 | 1.67818 | 1.64461 | 2.41688 | 1.27882 | 1.24525 | 2.01752 |
| 162475\_f\_at | Pglyrp1 | peptidoglycan recognition protein 1 | 21946 | 7 | 13 | N-acetylmuramoyl-L-alanine amidase activity  apoptosis  cytokine activity  extracellular space  immune response  peptidoglycan catabolism  peptidoglycan receptor activity  xenobiotic metabolism |  | 4.27704 | 4.40752 | 4.51559 | 6.10292 | 9.11142 | 5.09495 | 8.62661 | 6.6616 | 10.7065 | 9.6115 | 11.6807 | 12.0017 | 12.0896 | 11.5084 | 12.6768 | 11.3798 | 6.66336 | 6.10298 | 6.66453 | -2.38632 | -1.82594 | -2.38749 | -2.25584 | -1.69546 | -2.25701 | -2.14777 | -1.58738 | -2.14894 | -0.560438 | -5.59666e-05 | -0.561614 | 2.44807 | 3.00845 | 2.44689 | -1.56841 | -1.00802 | -1.56958 | 1.96326 | 2.52364 | 1.96208 | -0.0017602 | 0.558622 | -0.00293566 | 4.04319 | 4.60357 | 4.04202 | 2.94814 | 3.50852 | 2.94697 | 5.01735 | 5.57773 | 5.01618 | 5.33834 | 5.89873 | 5.33717 | 5.42622 | 5.9866 | 5.42505 | 4.84506 | 5.40544 | 4.84389 | 6.01344 | 6.57382 | 6.01226 | 4.71648 | 5.27687 | 4.71531 |
| 92275\_at | Tcfap2c | transcription factor AP-2, gamma | 21420 | 2 | 10 | DNA binding  embryonic development (sensu Mammalia)  nucleus  protein binding  regulation of transcription, DNA-dependent  transcription factor activity  transcription factor complex |  | 1.72449 | 2.98145 | 4.45158 | 6.35167 | 8.75561 | 5.08378 | 7.40956 | 5.19083 | 9.43023 | 10.0509 | 10.9257 | 11.2857 | 10.566 | 10.7113 | 10.6501 | 10.8347 | 8.32164 | 8.35822 | 8.40247 | -6.59715 | -6.63373 | -6.67798 | -5.34019 | -5.37676 | -5.42101 | -3.87006 | -3.90664 | -3.95089 | -1.96997 | -2.00654 | -2.0508 | 0.433966 | 0.397389 | 0.353138 | -3.23786 | -3.27444 | -3.31869 | -0.912083 | -0.948659 | -0.992911 | -3.13081 | -3.16739 | -3.21164 | 1.10859 | 1.07201 | 1.02776 | 1.72931 | 1.69273 | 1.64848 | 2.6041 | 2.56752 | 2.52327 | 2.9641 | 2.92752 | 2.88327 | 2.24437 | 2.2078 | 2.16355 | 2.38963 | 2.35305 | 2.3088 | 2.3285 | 2.29192 | 2.24767 | 2.51307 | 2.47649 | 2.43224 |
| 92440\_at | Irf6 | interferon regulatory factor 6 | 54139 | 1 | 5 | DNA binding  nucleus  regulation of transcription, DNA-dependent  transcription factor activity |  | 2.33108 | 2.40364 | 2.46392 | 2.70327 | 4.87055 | 2.4476 | 3.51155 | 2.40804 | 6.49714 | 5.70118 | 8.05039 | 7.5275 | 7.50688 | 7.58748 | 7.67986 | 6.5917 | 5.05564 | 4.8439 | 4.66289 | -2.72457 | -2.51283 | -2.33181 | -2.652 | -2.44027 | -2.25925 | -2.59172 | -2.37999 | -2.19897 | -2.35237 | -2.14063 | -1.95961 | -0.185092 | 0.0266458 | 0.20766 | -2.60804 | -2.3963 | -2.21529 | -1.54409 | -1.33235 | -1.15133 | -2.6476 | -2.43587 | -2.25485 | 1.4415 | 1.65324 | 1.83426 | 0.645544 | 0.857282 | 1.0383 | 2.99475 | 3.20649 | 3.3875 | 2.47186 | 2.6836 | 2.86461 | 2.45124 | 2.66298 | 2.84399 | 2.53184 | 2.74357 | 2.92459 | 2.62422 | 2.83596 | 3.01698 | 1.53606 | 1.7478 | 1.92881 |
| 92526\_f\_at | Btbd14a | BTB (POZ) domain containing 14A | 67991 | 2 | 6 | protein binding |  | 5.82456 | 5.13509 | 5.97388 | 5.93026 | 6.01133 | 5.92416 | 6.00576 | 5.57288 | 6.01526 | 6.56776 | 7.16116 | 7.20621 | 7.86399 | 7.47863 | 8.19757 | 7.99332 | 3.87908 | 3.40953 | 4.84141 | 1.94548 | 2.41503 | 0.983154 | 1.25602 | 1.72556 | 0.293686 | 2.09481 | 2.56435 | 1.13248 | 2.05118 | 2.52072 | 1.08885 | 2.13225 | 2.60179 | 1.16992 | 2.04509 | 2.51463 | 1.08276 | 2.12669 | 2.59623 | 1.16436 | 1.6938 | 2.16335 | 0.731472 | 2.13619 | 2.60573 | 1.17386 | 2.68868 | 3.15823 | 1.72635 | 3.28208 | 3.75163 | 2.31975 | 3.32713 | 3.79668 | 2.3648 | 3.98492 | 4.45446 | 3.02259 | 3.59956 | 4.0691 | 2.63723 | 4.31849 | 4.78804 | 3.35616 | 4.11425 | 4.58379 | 3.15192 |
| 92554\_at | Ctbp2 | C-terminal binding protein 2 | 13017 | 7 | 8 | L-serine biosynthesis  nucleus  oxidoreductase activity  oxidoreductase activity, acting on the CH-OH group of donors, NAD or NADP as acceptor  protein binding  protein binding  transcription corepressor activity | Wnt signaling pathway | 6.42139 | 6.82232 | 7.67302 | 6.70309 | 7.98304 | 7.20004 | 7.53019 | 6.78267 | 8.01242 | 8.4091 | 9.50133 | 9.50387 | 9.27942 | 9.15078 | 10.1075 | 9.63267 | 7.57788 | 7.58858 | 7.53023 | -1.15648 | -1.16718 | -1.10884 | -0.755559 | -0.76626 | -0.707917 | 0.095146 | 0.0844455 | 0.142788 | -0.874786 | -0.885487 | -0.827144 | 0.40516 | 0.39446 | 0.452803 | -0.37784 | -0.38854 | -0.330197 | -0.0476904 | -0.0583909 | -4.79242e-05 | -0.795201 | -0.805901 | -0.747559 | 0.434541 | 0.423841 | 0.482184 | 0.831227 | 0.820526 | 0.878869 | 1.92345 | 1.91275 | 1.97109 | 1.92599 | 1.91529 | 1.97364 | 1.70154 | 1.69084 | 1.74918 | 1.57291 | 1.56221 | 1.62055 | 2.52958 | 2.51888 | 2.57723 | 2.05479 | 2.04409 | 2.10243 |
| 92851\_at | Cp | ceruloplasmin | 12870 | 9 | 11 | copper ion binding  copper ion transport  copper ion transporter activity  extracellular space  ferroxidase activity  ion transport  oxidoreductase activity  transport | Porphyrin and chlorophyll metabolism | 8.949 | 8.10969 | 9.79695 | 10.2699 | 11.0231 | 10.1766 | 10.828 | 10.1898 | 11.801 | 11.6433 | 12.2332 | 12.0139 | 12.2877 | 11.9008 | 11.7723 | 12.6962 | 11.1476 | 11.3714 | 10.9838 | -2.19863 | -2.42243 | -2.03476 | -3.03793 | -3.26174 | -2.87406 | -1.35067 | -1.57448 | -1.1868 | -0.877735 | -1.10154 | -0.713867 | -0.124509 | -0.348318 | 0.0393597 | -0.970994 | -1.1948 | -0.807125 | -0.319596 | -0.543405 | -0.155727 | -0.957783 | -1.18159 | -0.793914 | 0.653367 | 0.429558 | 0.817236 | 0.495641 | 0.271832 | 0.65951 | 1.08558 | 0.861776 | 1.24945 | 0.866238 | 0.64243 | 1.03011 | 1.14003 | 0.916222 | 1.3039 | 0.753142 | 0.529333 | 0.917011 | 0.624668 | 0.400859 | 0.788536 | 1.5486 | 1.3248 | 1.71247 |
| 92870\_at | Sel1h | Sel1 (suppressor of lin-12) 1 homolog (C. elegans) | 20338 | 12 | 8 | extracellular space  integral to membrane |  | 4.17677 | 4.90641 | 4.95611 | 4.78565 | 4.85851 | 5.22549 | 4.83273 | 4.87355 | 6.8988 | 10.032 | 9.28074 | 9.83165 | 8.6101 | 10.8608 | 10.6033 | 9.34952 | 4.76233 | 4.75858 | 4.74793 | -0.585561 | -0.581813 | -0.571159 | 0.144086 | 0.147834 | 0.158488 | 0.193784 | 0.197531 | 0.208186 | 0.0233235 | 0.0270713 | 0.0377255 | 0.0961791 | 0.099927 | 0.110581 | 0.463161 | 0.466909 | 0.477563 | 0.0704071 | 0.0741549 | 0.0848091 | 0.11122 | 0.114968 | 0.125622 | 2.13647 | 2.14022 | 2.15087 | 5.26969 | 5.27343 | 5.28409 | 4.51841 | 4.52216 | 4.53282 | 5.06932 | 5.07307 | 5.08372 | 3.84777 | 3.85152 | 3.86217 | 6.09846 | 6.10221 | 6.11286 | 5.84097 | 5.84472 | 5.85537 | 4.58719 | 4.59094 | 4.60159 |
| 92871\_at | Sel1h | Sel1 (suppressor of lin-12) 1 homolog (C. elegans) | 20338 | 12 | 8 | extracellular space  integral to membrane |  | 5.17604 | 5.29471 | 5.25994 | 5.64447 | 6.73497 | 5.61431 | 6.17721 | 5.8966 | 8.27428 | 8.54096 | 9.39348 | 10.0009 | 9.46791 | 9.78059 | 10.03 | 9.10883 | 5.36615 | 5.22697 | 5.0276 | -0.19011 | -0.050933 | 0.148439 | -0.071446 | 0.0677305 | 0.267103 | -0.106215 | 0.0329618 | 0.232334 | 0.278315 | 0.417492 | 0.616864 | 1.36882 | 1.508 | 1.70737 | 0.248163 | 0.387339 | 0.586711 | 0.811056 | 0.950233 | 1.1496 | 0.530451 | 0.669628 | 0.869 | 2.90813 | 3.04731 | 3.24668 | 3.17481 | 3.31399 | 3.51336 | 4.02733 | 4.16651 | 4.36588 | 4.63475 | 4.77392 | 4.97329 | 4.10176 | 4.24094 | 4.44031 | 4.41444 | 4.55362 | 4.75299 | 4.6639 | 4.80307 | 5.00245 | 3.74268 | 3.88185 | 4.08123 |
| 92880\_at | Mfge8 | milk fat globule-EGF factor 8 protein | 17304 | 7 | 13 | cell adhesion  external side of plasma membrane  extracellular space  extracellular space  extrinsic to plasma membrane  fertilization (sensu Animalia)  integrin binding  phagocytosis, engulfment  phagocytosis, recognition  phosphatidylethanolamine binding  phosphatidylserine binding  positive regulation of phagocytosis  protein binding |  | 9.83616 | 10.3168 | 10.3374 | 11.1365 | 11.8293 | 10.8715 | 10.8501 | 10.7003 | 12.0558 | 11.819 | 12.4219 | 12.7352 | 12.8003 | 11.9542 | 12.375 | 12.7486 | 12.3152 | 12.3758 | 12.302 | -2.479 | -2.53964 | -2.46587 | -1.9984 | -2.05903 | -1.98526 | -1.97778 | -2.03842 | -1.96465 | -1.1787 | -1.23933 | -1.16556 | -0.485891 | -0.546525 | -0.472754 | -1.44369 | -1.50432 | -1.43055 | -1.46506 | -1.52569 | -1.45192 | -1.61482 | -1.67545 | -1.60168 | -0.259331 | -0.319964 | -0.246193 | -0.496123 | -0.556757 | -0.482986 | 0.106705 | 0.046071 | 0.119842 | 0.420058 | 0.359425 | 0.433195 | 0.48516 | 0.424526 | 0.498297 | -0.360996 | -0.42163 | -0.347859 | 0.0597934 | -0.000840414 | 0.0729305 | 0.433481 | 0.372848 | 0.446619 |
| 92927\_at | Etv1 | ets variant gene 1 | 14009 | 12 | 7 | DNA binding  axon guidance  mechanosensory behavior  muscle development  nucleus  regulation of transcription, DNA-dependent  transcription factor activity |  | 3.37705 | 3.44393 | 3.53202 | 3.37376 | 3.47131 | 3.50127 | 3.45495 | 3.45606 | 5.39506 | 7.65191 | 8.45274 | 8.74146 | 8.14704 | 9.46831 | 9.67099 | 8.77525 | 3.33605 | 3.34917 | 3.30534 | 0.041 | 0.0278796 | 0.0717121 | 0.107883 | 0.0947623 | 0.138595 | 0.195974 | 0.182853 | 0.226686 | 0.0377098 | 0.0245895 | 0.068422 | 0.135262 | 0.122141 | 0.165974 | 0.165218 | 0.152098 | 0.195931 | 0.118905 | 0.105785 | 0.149617 | 0.120007 | 0.106887 | 0.150719 | 2.05901 | 2.04589 | 2.08973 | 4.31586 | 4.30274 | 4.34657 | 5.11669 | 5.10357 | 5.1474 | 5.40541 | 5.39229 | 5.43612 | 4.81099 | 4.79787 | 4.84171 | 6.13227 | 6.11915 | 6.16298 | 6.33494 | 6.32182 | 6.36566 | 5.4392 | 5.42608 | 5.46991 |
| 92931\_at | Dll1 | delta-like 1 (Drosophila) | 13388 | 17 | 14 | calcium ion binding  cell communication  cell differentiation  compartment specification  determination of left/right symmetry  development  extracellular space  integral to membrane  membrane  protein binding  somite specification |  | 2.61178 | 2.84793 | 2.87816 | 2.93726 | 3.28331 | 2.94121 | 2.99017 | 2.87891 | 4.50284 | 5.68065 | 7.01326 | 7.82983 | 7.37703 | 7.74577 | 6.702 | 7.26258 | 3.18789 | 3.387 | 2.98796 | -0.576102 | -0.775212 | -0.37618 | -0.339955 | -0.539065 | -0.140033 | -0.309725 | -0.508835 | -0.109803 | -0.250623 | -0.449733 | -0.0507007 | 0.0954292 | -0.103681 | 0.295351 | -0.246673 | -0.445784 | -0.0467513 | -0.197716 | -0.396826 | 0.00220624 | -0.30898 | -0.50809 | -0.109057 | 1.31496 | 1.11585 | 1.51488 | 2.49277 | 2.29366 | 2.69269 | 3.82538 | 3.62627 | 4.0253 | 4.64194 | 4.44283 | 4.84186 | 4.18915 | 3.99004 | 4.38907 | 4.55788 | 4.35877 | 4.75781 | 3.51412 | 3.31501 | 3.71404 | 4.07469 | 3.87558 | 4.27461 |
| 93013\_at | Idb2 | inhibitor of DNA binding 2 | 15902 | 12 | 17 | cytoplasm  development  lymph gland development  nucleus | TGF-beta signaling pathway | 9.85182 | 10.1784 | 10.4914 | 10.0054 | 10.9292 | 10.1918 | 10.5516 | 10.4866 | 11.133 | 11.7068 | 11.9912 | 12.0745 | 11.4887 | 11.8577 | 12.024 | 11.8255 | 11.0573 | 11.0143 | 10.2665 | -1.20546 | -1.16246 | -0.414681 | -0.878887 | -0.835885 | -0.088103 | -0.565872 | -0.522871 | 0.224911 | -1.05184 | -1.00884 | -0.261061 | -0.12812 | -0.0851185 | 0.662663 | -0.86547 | -0.822469 | -0.0746869 | -0.505693 | -0.462691 | 0.285091 | -0.570695 | -0.527694 | 0.220088 | 0.0756894 | 0.118691 | 0.866473 | 0.64954 | 0.692542 | 1.44032 | 0.933895 | 0.976897 | 1.72468 | 1.01721 | 1.06021 | 1.80799 | 0.431444 | 0.474446 | 1.22223 | 0.800389 | 0.843391 | 1.59117 | 0.966747 | 1.00975 | 1.75753 | 0.768266 | 0.811268 | 1.55905 |
| 93025\_at | Ndfip1 | Nedd4 family interacting protein 1 | 65113 | 18 | 7 | biological\_process unknown  integral to membrane  protein binding |  | 10.6504 | 10.6242 | 10.9172 | 10.6378 | 10.5573 | 10.8572 | 11.0024 | 10.7115 | 11.0244 | 11.3492 | 11.5259 | 11.3676 | 11.2892 | 11.4409 | 11.4537 | 11.6143 | 10.8635 | 11.3242 | 10.8719 | -0.213136 | -0.673765 | -0.221534 | -0.239283 | -0.699913 | -0.247682 | 0.053661 | -0.406968 | 0.0452625 | -0.22578 | -0.686409 | -0.234178 | -0.306264 | -0.766894 | -0.314663 | -0.00630628 | -0.466935 | -0.0147048 | 0.138829 | -0.3218 | 0.13043 | -0.152046 | -0.612675 | -0.160445 | 0.16091 | -0.299719 | 0.152511 | 0.485717 | 0.0250877 | 0.477318 | 0.662335 | 0.201706 | 0.653936 | 0.504091 | 0.0434617 | 0.495692 | 0.425666 | -0.0349627 | 0.417268 | 0.577342 | 0.116713 | 0.568943 | 0.590145 | 0.129516 | 0.581747 | 0.750796 | 0.290167 | 0.742398 |
| 93059\_at | 2610204K14Rik | RIKEN cDNA 2610204K14 gene | 67148 | 7 | 5 |  |  | 5.36403 | 5.50355 | 5.44835 | 5.5713 | 5.79894 | 5.41312 | 5.94899 | 6.12394 | 6.19589 | 7.02282 | 6.86577 | 7.1523 | 6.16833 | 6.65308 | 7.29278 | 6.93103 | 6.24185 | 6.77521 | 5.67476 | -0.877828 | -1.41119 | -0.31073 | -0.738301 | -1.27166 | -0.171203 | -0.793505 | -1.32686 | -0.226407 | -0.670557 | -1.20391 | -0.103459 | -0.442916 | -0.976273 | 0.124182 | -0.82873 | -1.36209 | -0.261633 | -0.292866 | -0.826223 | 0.274231 | -0.117917 | -0.651274 | 0.44918 | -0.045964 | -0.579321 | 0.521134 | 0.780969 | 0.247612 | 1.34807 | 0.623913 | 0.0905561 | 1.19101 | 0.91045 | 0.377093 | 1.47755 | -0.07352 | -0.606877 | 0.493577 | 0.411222 | -0.122135 | 0.97832 | 1.05092 | 0.517568 | 1.61802 | 0.689174 | 0.155817 | 1.25627 |
| 93063\_at | App | amyloid beta (A4) precursor protein | 11820 | 16 | 47 | apoptosis  binding  cell adhesion  coated pit  endocytosis  heparin binding  integral to membrane  membrane  protein binding  serine-type endopeptidase inhibitor activity | Neurodegenerative Disorders | 10.3543 | 10.6367 | 11.2013 | 10.9411 | 11.3399 | 11.0879 | 10.8428 | 10.9528 | 11.8925 | 11.8966 | 12.0177 | 12.2639 | 12.0706 | 11.9509 | 12.1469 | 12.5645 | 11.2749 | 11.1432 | 11.1899 | -0.920587 | -0.788923 | -0.835653 | -0.638113 | -0.506449 | -0.553178 | -0.073529 | 0.0581356 | 0.0114058 | -0.333726 | -0.202061 | -0.248791 | 0.0650516 | 0.196716 | 0.149986 | -0.186913 | -0.0552485 | -0.101978 | -0.43202 | -0.300356 | -0.347086 | -0.322066 | -0.190402 | -0.237131 | 0.617692 | 0.749356 | 0.702627 | 0.621698 | 0.753363 | 0.706633 | 0.742848 | 0.874513 | 0.827783 | 0.989016 | 1.12068 | 1.07395 | 0.795762 | 0.927426 | 0.880696 | 0.676029 | 0.807694 | 0.760964 | 0.872071 | 1.00374 | 0.957006 | 1.28961 | 1.42127 | 1.37454 |
| 93220\_at | Col4a5 | procollagen, type IV, alpha 5 | 12830 | X | 12 | basement membrane  basement membrane  cell adhesion  collagen  collagen type IV  extracellular matrix  extracellular matrix structural constituent  extracellular matrix structural constituent conferring tensile strength  extracellular space  membrane |  | 3.65693 | 3.73052 | 3.83061 | 3.64905 | 3.64767 | 3.77956 | 3.75828 | 3.75585 | 5.13531 | 6.75803 | 7.40296 | 7.23914 | 4.88844 | 8.40402 | 7.67912 | 8.42482 | 3.62058 | 3.63306 | 3.57948 | 0.0363555 | 0.0238747 | 0.0774512 | 0.109942 | 0.0974608 | 0.151037 | 0.210031 | 0.19755 | 0.251126 | 0.0284717 | 0.0159909 | 0.0695674 | 0.0270956 | 0.0146149 | 0.0681913 | 0.158978 | 0.146497 | 0.200074 | 0.137702 | 0.125221 | 0.178798 | 0.135272 | 0.122791 | 0.176367 | 1.51474 | 1.50225 | 1.55583 | 3.13746 | 3.12498 | 3.17855 | 3.78239 | 3.76991 | 3.82348 | 3.61857 | 3.60608 | 3.65966 | 1.26786 | 1.25538 | 1.30895 | 4.78344 | 4.77096 | 4.82454 | 4.05854 | 4.04606 | 4.09964 | 4.80424 | 4.79176 | 4.84533 |
| 93281\_at | Rcn2 | reticulocalbin 2 | 26611 | 9 | 6 | calcium ion binding  endoplasmic reticulum  extracellular space |  | 6.87361 | 6.93177 | 7.59896 | 7.49816 | 7.98667 | 7.43237 | 7.75215 | 7.21233 | 8.48032 | 8.33806 | 9.47967 | 9.5095 | 9.01427 | 9.66142 | 9.12687 | 9.67238 | 5.8534 | 5.84971 | 5.76804 | 1.02021 | 1.0239 | 1.10557 | 1.07838 | 1.08206 | 1.16374 | 1.74556 | 1.74925 | 1.83093 | 1.64476 | 1.64845 | 1.73013 | 2.13328 | 2.13697 | 2.21864 | 1.57897 | 1.58266 | 1.66433 | 1.89875 | 1.90244 | 1.98411 | 1.35894 | 1.36263 | 1.4443 | 2.62692 | 2.63061 | 2.71229 | 2.48467 | 2.48836 | 2.57003 | 3.62628 | 3.62997 | 3.71164 | 3.65611 | 3.65979 | 3.74147 | 3.16087 | 3.16456 | 3.24624 | 3.80803 | 3.81171 | 3.89339 | 3.27347 | 3.27716 | 3.35883 | 3.81898 | 3.82267 | 3.90435 |
| 93285\_at | Dusp6 | dual specificity phosphatase 6 | 67603 | 10 | 10 | MAP kinase phosphatase activity  hydrolase activity  phosphoprotein phosphatase activity  protein amino acid dephosphorylation  protein tyrosine/serine/threonine phosphatase activity | MAPK signaling pathway | 6.79904 | 9.11933 | 9.70698 | 9.79239 | 11.1767 | 9.69673 | 9.94234 | 9.77594 | 11.7742 | 12.0507 | 12.6633 | 13.1278 | 12.5969 | 12.6203 | 12.6734 | 13.0137 | 5.40391 | 4.69486 | 5.35079 | 1.39513 | 2.10418 | 1.44825 | 3.71542 | 4.42447 | 3.76854 | 4.30307 | 5.01213 | 4.35619 | 4.38847 | 5.09753 | 4.4416 | 5.77283 | 6.48189 | 5.82595 | 4.29282 | 5.00187 | 4.34594 | 4.53843 | 5.24749 | 4.59156 | 4.37203 | 5.08109 | 4.42516 | 6.37033 | 7.07938 | 6.42345 | 6.64678 | 7.35583 | 6.6999 | 7.25941 | 7.96847 | 7.31254 | 7.72392 | 8.43298 | 7.77705 | 7.19297 | 7.90202 | 7.24609 | 7.21638 | 7.92543 | 7.2695 | 7.26944 | 7.9785 | 7.32257 | 7.60981 | 8.31887 | 7.66294 |
| 93298\_at | Papss1 | 3'-phosphoadenosine 5'-phosphosulfate synthase 1 | 23971 | 3 | 3 | ATP binding  catalytic activity  kinase activity  nucleotidyltransferase activity  sulfate adenylyltransferase (ATP) activity  sulfate assimilation  transferase activity  transferase activity, transferring phosphorus-containing groups | Purine metabolism  Selenoamino acid metabolism  Sulfur metabolism | 5.70676 | 5.68971 | 6.45356 | 6.08141 | 7.41647 | 6.19679 | 7.01065 | 6.40567 | 8.84453 | 8.83315 | 9.26327 | 9.71465 | 9.12337 | 8.99546 | 9.16333 | 9.59441 | 6.81156 | 6.18247 | 5.96138 | -1.1048 | -0.475703 | -0.254621 | -1.12186 | -0.49276 | -0.271678 | -0.358002 | 0.271094 | 0.492177 | -0.730151 | -0.101055 | 0.120028 | 0.604911 | 1.23401 | 1.45509 | -0.614777 | 0.0143193 | 0.235402 | 0.199084 | 0.828181 | 1.04926 | -0.405888 | 0.223209 | 0.444291 | 2.03297 | 2.66206 | 2.88315 | 2.02158 | 2.65068 | 2.87176 | 2.4517 | 3.0808 | 3.30188 | 2.90309 | 3.53218 | 3.75327 | 2.31181 | 2.9409 | 3.16199 | 2.18389 | 2.81299 | 3.03407 | 2.35177 | 2.98087 | 3.20195 | 2.78284 | 3.41194 | 3.63302 |
| 93323\_at | Plp2 | proteolipid protein 2 | 18824 | X | 6 | integral to membrane |  | 7.94161 | 7.49079 | 8.00333 | 7.94126 | 7.88053 | 8.03648 | 8.0038 | 7.56803 | 9.41074 | 9.54794 | 9.13994 | 9.65751 | 9.71704 | 9.19122 | 10.0638 | 10.126 | 7.95621 | 7.8366 | 8.34172 | -0.0146039 | 0.105003 | -0.40011 | -0.465424 | -0.345818 | -0.85093 | 0.0471169 | 0.166723 | -0.338389 | -0.0149486 | 0.104658 | -0.400454 | -0.0756789 | 0.0439277 | -0.461185 | 0.0802739 | 0.19988 | -0.305232 | 0.0475931 | 0.1672 | -0.337913 | -0.388182 | -0.268575 | -0.773687 | 1.45453 | 1.57414 | 1.06902 | 1.59173 | 1.71133 | 1.20622 | 1.18373 | 1.30333 | 0.798221 | 1.7013 | 1.8209 | 1.31579 | 1.76082 | 1.88043 | 1.37532 | 1.23501 | 1.35461 | 0.8495 | 2.10763 | 2.22724 | 1.72213 | 2.16974 | 2.28935 | 1.78424 |
| 93336\_at | 1110014C03Rik | RIKEN cDNA 1110014C03 gene | 68581 | 12 | 6 | integral to membrane |  | 9.84359 | 9.181 | 9.35981 | 9.66642 | 10.0552 | 9.88447 | 10.3249 | 10.1259 | 11.1055 | 10.8374 | 11.09 | 11.0968 | 11.1316 | 10.7166 | 11.1234 | 11.1106 | 10.8962 | 10.7681 | 10.7819 | -1.05265 | -0.924478 | -0.938343 | -1.71523 | -1.58706 | -1.60093 | -1.53642 | -1.40825 | -1.42212 | -1.22981 | -1.10164 | -1.11551 | -0.840993 | -0.712824 | -0.726689 | -1.01176 | -0.88359 | -0.897454 | -0.571305 | -0.443136 | -0.457001 | -0.770283 | -0.642114 | -0.655979 | 0.209265 | 0.337434 | 0.323569 | -0.0588206 | 0.069348 | 0.0554832 | 0.193733 | 0.321902 | 0.308037 | 0.200574 | 0.328742 | 0.314877 | 0.235396 | 0.363564 | 0.3497 | -0.179613 | -0.0514447 | -0.0653095 | 0.227151 | 0.355319 | 0.341455 | 0.21432 | 0.342488 | 0.328624 |
| 93341\_r\_at | Copb2 | coatomer protein complex, subunit beta 2 (beta prime) | 50797 | 9 | 6 | COPI vesicle coat  Golgi apparatus  endoplasmic reticulum  intracellular protein transport  membrane  protein transport  protein transporter activity  transport |  | 9.30491 | 9.0695 | 9.03621 | 9.55256 | 9.0945 | 9.37202 | 9.53928 | 9.20192 | 9.69807 | 9.53678 | 9.77719 | 9.85933 | 9.83372 | 9.65075 | 9.76413 | 9.94138 | 10.3437 | 9.89793 | 10.0237 | -1.0388 | -0.593024 | -0.71877 | -1.27421 | -0.828431 | -0.954177 | -1.3075 | -0.861724 | -0.98747 | -0.791147 | -0.345367 | -0.471114 | -1.24921 | -0.803434 | -0.929181 | -0.971689 | -0.525909 | -0.651656 | -0.804433 | -0.358654 | -0.4844 | -1.14179 | -0.69601 | -0.821756 | -0.645639 | -0.199859 | -0.325606 | -0.80693 | -0.36115 | -0.486897 | -0.566522 | -0.120742 | -0.246488 | -0.484375 | -0.0385958 | -0.164342 | -0.509988 | -0.0642088 | -0.189955 | -0.692956 | -0.247177 | -0.372923 | -0.579576 | -0.133796 | -0.259543 | -0.402325 | 0.0434549 | -0.0822915 |
| 93389\_at | Prom1 | prominin 1 | 19126 | 5 | 8 | brush border  integral to membrane  integral to plasma membrane  microvillus  phototransduction |  | 2.56406 | 2.66974 | 2.75897 | 2.66145 | 2.95734 | 2.65354 | 2.61574 | 2.64398 | 5.35104 | 6.61129 | 7.5378 | 7.98544 | 7.11323 | 7.57686 | 7.91523 | 7.27239 | 2.53764 | 2.53612 | 2.5027 | 0.0264174 | 0.0279398 | 0.0613593 | 0.132095 | 0.133618 | 0.167037 | 0.221323 | 0.222845 | 0.256265 | 0.12381 | 0.125332 | 0.158752 | 0.419697 | 0.42122 | 0.454639 | 0.115897 | 0.117419 | 0.150839 | 0.078091 | 0.0796135 | 0.113033 | 0.106338 | 0.10786 | 0.14128 | 2.81339 | 2.81492 | 2.84834 | 4.07365 | 4.07517 | 4.10859 | 5.00016 | 5.00168 | 5.0351 | 5.4478 | 5.44932 | 5.48274 | 4.57559 | 4.57711 | 4.61053 | 5.03922 | 5.04074 | 5.07416 | 5.37758 | 5.3791 | 5.41252 | 4.73474 | 4.73627 | 4.76969 |
| 93604\_f\_at | Igsf4a | immunoglobulin superfamily, member 4A | 54725 | 9 | 14 | calcium-independent cell-cell adhesion  cell adhesion  integral to membrane  protein binding  synapse  synaptic vesicle  synaptogenesis |  | 1.40729 | 1.6599 | 2.74142 | 2.66537 | 4.3594 | 2.87442 | 4.27281 | 2.57443 | 4.60188 | 6.24069 | 6.22729 | 5.11585 | 6.00873 | 6.52778 | 6.38423 | 6.92388 | 2.22657 | 4.84007 | 4.7787 | -0.819284 | -3.43278 | -3.37141 | -0.566675 | -3.18017 | -3.11881 | 0.514849 | -2.09864 | -2.03728 | 0.438792 | -2.1747 | -2.11334 | 2.13282 | -0.48067 | -0.419308 | 0.647844 | -1.96565 | -1.90429 | 2.04623 | -0.567258 | -0.505896 | 0.347858 | -2.26563 | -2.20427 | 2.37531 | -0.238183 | -0.176821 | 4.01412 | 1.40063 | 1.46199 | 4.00072 | 1.38722 | 1.44859 | 2.88928 | 0.275785 | 0.337147 | 3.78216 | 1.16867 | 1.23003 | 4.30121 | 1.68772 | 1.74908 | 4.15765 | 1.54416 | 1.60552 | 4.69731 | 2.08382 | 2.14518 |
| 93612\_at | Mmp15 | matrix metalloproteinase 15 | 17388 | 8 | 7 | extracellular matrix  extracellular space  hydrolase activity  integral to membrane  metalloendopeptidase activity  metallopeptidase activity  proteolysis and peptidolysis  zinc ion binding |  | 2.53672 | 2.59947 | 2.67804 | 2.53797 | 2.58376 | 2.60392 | 2.5661 | 2.6047 | 2.70996 | 4.3062 | 5.25793 | 6.10566 | 5.80361 | 8.6235 | 7.17253 | 8.33167 | 2.51371 | 2.51469 | 2.54858 | 0.0230117 | 0.022025 | -0.0118653 | 0.08576 | 0.0847734 | 0.050883 | 0.164332 | 0.163345 | 0.129455 | 0.0242624 | 0.0232757 | -0.0106146 | 0.0700511 | 0.0690645 | 0.0351742 | 0.0902146 | 0.089228 | 0.0553377 | 0.0523913 | 0.0514047 | 0.0175144 | 0.0909966 | 0.09001 | 0.0561196 | 0.196254 | 0.195268 | 0.161378 | 1.79249 | 1.79151 | 1.75762 | 2.74422 | 2.74323 | 2.70934 | 3.59196 | 3.59097 | 3.55708 | 3.2899 | 3.28892 | 3.25503 | 6.10979 | 6.10881 | 6.07492 | 4.65883 | 4.65784 | 4.62395 | 5.81797 | 5.81698 | 5.78309 |
| 93689\_at | Emid1 | EMI domain containing 1 | 140703 | 11 | 4 | Golgi apparatus  cytoplasm  endoplasmic reticulum  extracellular matrix  extracellular space  phosphate transport  protein binding |  | 2.92895 | 3.31413 | 3.22634 | 3.79523 | 5.04083 | 3.5019 | 3.47317 | 3.26149 | 5.88783 | 5.01678 | 7.07062 | 7.36745 | 7.26458 | 6.91018 | 7.55615 | 7.3376 | 3.62821 | 3.156 | 4.01425 | -0.699256 | -0.227046 | -1.0853 | -0.314075 | 0.158134 | -0.700116 | -0.401865 | 0.0703447 | -0.787906 | 0.167024 | 0.639233 | -0.219017 | 1.41262 | 1.88483 | 1.02658 | -0.126305 | 0.345905 | -0.512346 | -0.155037 | 0.317172 | -0.541078 | -0.366712 | 0.105497 | -0.752753 | 2.25963 | 2.73183 | 1.87358 | 1.38858 | 1.86078 | 1.00253 | 3.44241 | 3.91462 | 3.05637 | 3.73924 | 4.21145 | 3.3532 | 3.63638 | 4.10859 | 3.25034 | 3.28198 | 3.75419 | 2.89594 | 3.92794 | 4.40015 | 3.5419 | 3.7094 | 4.18161 | 3.32336 |
| 93732\_f\_at | Rgs19ip1 | regulator of G-protein signaling 19 interacting protein 1 | 67903 | 8 | 8 |  |  | 3.82623 | 4.06487 | 4.36091 | 3.68821 | 4.2558 | 4.33004 | 4.18726 | 3.59072 | 5.41831 | 5.42143 | 5.43967 | 5.93406 | 7.36764 | 6.44932 | 7.59917 | 6.74692 | 5.32295 | 5.86365 | 6.19739 | -1.49673 | -2.03742 | -2.37117 | -1.25808 | -1.79877 | -2.13252 | -0.96204 | -1.50273 | -1.83648 | -1.63475 | -2.17544 | -2.50919 | -1.06716 | -1.60785 | -1.9416 | -0.992918 | -1.53361 | -1.86736 | -1.1357 | -1.67639 | -2.01014 | -1.73223 | -2.27292 | -2.60667 | 0.0953566 | -0.445338 | -0.779084 | 0.0984728 | -0.442221 | -0.775967 | 0.116721 | -0.423973 | -0.757719 | 0.611105 | 0.0704103 | -0.263336 | 2.04469 | 1.504 | 1.17025 | 1.12637 | 0.585672 | 0.251926 | 2.27622 | 1.73552 | 1.40178 | 1.42397 | 0.883272 | 0.549526 |
| 93747\_at | na | hypothetical LOC386552 | 386552 | 9 |  |  |  | 6.56987 | 6.6083 | 6.59816 | 6.67937 | 7.47939 | 6.59249 | 7.26485 | 6.57688 | 8.02575 | 8.0247 | 8.82808 | 8.70102 | 8.3939 | 8.68365 | 8.87573 | 8.82442 | 8.25336 | 8.45284 | 8.87421 | -1.68349 | -1.88296 | -2.30434 | -1.64506 | -1.84454 | -2.26591 | -1.6552 | -1.85467 | -2.27605 | -1.574 | -1.77347 | -2.19484 | -0.773976 | -0.973448 | -1.39482 | -1.66088 | -1.86035 | -2.28173 | -0.988513 | -1.18798 | -1.60936 | -1.67649 | -1.87596 | -2.29733 | -0.227617 | -0.427088 | -0.848463 | -0.228666 | -0.428137 | -0.849512 | 0.574715 | 0.375243 | -0.0461313 | 0.447661 | 0.24819 | -0.173185 | 0.140538 | -0.0589327 | -0.480308 | 0.430286 | 0.230815 | -0.19056 | 0.622366 | 0.422894 | 0.00151964 | 0.571061 | 0.37159 | -0.0497852 |
| 93753\_at | Litaf | LPS-induced TN factor | 56722 | 16 | 8 | biological\_process unknown  cellular\_component unknown  protein binding |  | 7.98058 | 8.02395 | 8.83453 | 9.32409 | 9.99085 | 9.25942 | 9.28792 | 8.85804 | 10.6077 | 10.3892 | 11.287 | 11.1758 | 11.2143 | 10.6841 | 10.8838 | 10.6879 | 9.7468 | 9.79574 | 9.67997 | -1.76623 | -1.81516 | -1.69939 | -1.72285 | -1.77179 | -1.65601 | -0.912276 | -0.961213 | -0.845439 | -0.422714 | -0.471651 | -0.355877 | 0.24405 | 0.195113 | 0.310887 | -0.487384 | -0.536321 | -0.420546 | -0.458881 | -0.507818 | -0.392043 | -0.888766 | -0.937703 | -0.821929 | 0.860895 | 0.811958 | 0.927733 | 0.642373 | 0.593436 | 0.70921 | 1.54024 | 1.4913 | 1.60707 | 1.42901 | 1.38007 | 1.49585 | 1.46754 | 1.41861 | 1.53438 | 0.937321 | 0.888384 | 1.00416 | 1.13702 | 1.08808 | 1.20386 | 0.941144 | 0.892207 | 1.00798 |
| 93782\_at | Rnf4 | ring finger protein 4 | 19822 | 5 | 13 | nucleus  protein ubiquitination  regulation of transcription, DNA-dependent  ubiquitin ligase complex  ubiquitin-protein ligase activity  zinc ion binding |  | 7.96802 | 7.42634 | 7.80826 | 7.62971 | 7.55158 | 7.93131 | 8.08784 | 8.17644 | 8.48389 | 9.05709 | 8.86706 | 8.98756 | 8.27064 | 8.99811 | 9.26296 | 8.80984 | 7.48101 | 7.84889 | 7.00397 | 0.487007 | 0.119129 | 0.964044 | -0.0546685 | -0.422546 | 0.422368 | 0.327246 | -0.0406317 | 0.804283 | 0.148696 | -0.219182 | 0.625733 | 0.0705738 | -0.297304 | 0.547611 | 0.450302 | 0.0824237 | 0.927339 | 0.606826 | 0.238948 | 1.08386 | 0.69543 | 0.327552 | 1.17247 | 1.00288 | 0.635005 | 1.47992 | 1.57608 | 1.2082 | 2.05312 | 1.38605 | 1.01817 | 1.86309 | 1.50655 | 1.13868 | 1.98359 | 0.789625 | 0.421747 | 1.26666 | 1.5171 | 1.14922 | 1.99414 | 1.78195 | 1.41407 | 2.25898 | 1.32883 | 0.960948 | 1.80586 |
| 93785\_at | Folr1 | folate receptor 1 (adult) | 14275 | 7 | 13 | extracellular space  folate transporter activity  folic acid binding  folic acid metabolism  membrane  membrane fraction  posttranslational membrane targeting  receptor activity |  | 2.59162 | 2.66077 | 2.56629 | 2.98438 | 5.98095 | 2.71479 | 2.97484 | 2.66877 | 7.94657 | 9.08311 | 10.0045 | 10.568 | 10.3147 | 10.0888 | 10.6118 | 10.4136 | 10.5091 | 10.8119 | 10.46 | -7.91752 | -8.2203 | -7.86834 | -7.84837 | -8.15115 | -7.79919 | -7.94285 | -8.24563 | -7.89367 | -7.52476 | -7.82754 | -7.47558 | -4.52819 | -4.83097 | -4.47902 | -7.79435 | -8.09713 | -7.74518 | -7.5343 | -7.83708 | -7.48513 | -7.84037 | -8.14315 | -7.7912 | -2.56257 | -2.86535 | -2.5134 | -1.42602 | -1.72881 | -1.37685 | -0.504635 | -0.807418 | -0.455461 | 0.0588653 | -0.243918 | 0.108039 | -0.194443 | -0.497226 | -0.145269 | -0.420366 | -0.723149 | -0.371192 | 0.102709 | -0.200074 | 0.151883 | -0.0955402 | -0.398323 | -0.046366 |
| 93819\_at | 1110032D12Rik | RIKEN cDNA 1110032D12 gene | 56334 | 5 | 5 | Golgi apparatus  integral to membrane  intracellular protein transport  membrane  protein carrier activity  protein transport  transport |  | 10.6396 | 10.6861 | 10.3495 | 10.8353 | 11.1785 | 10.8671 | 10.6285 | 10.6526 | 11.1061 | 11.3786 | 11.3888 | 11.6994 | 11.5495 | 11.6883 | 11.5251 | 11.8269 | 11.5125 | 11.612 | 11.3483 | -0.872865 | -0.972442 | -0.708663 | -0.826323 | -0.9259 | -0.662121 | -1.16299 | -1.26256 | -0.998783 | -0.677166 | -0.776742 | -0.512963 | -0.333917 | -0.433494 | -0.169715 | -0.64541 | -0.744987 | -0.481208 | -0.883969 | -0.983545 | -0.719766 | -0.859884 | -0.959461 | -0.695682 | -0.406341 | -0.505918 | -0.242139 | -0.133908 | -0.233485 | 0.0302944 | -0.12368 | -0.223256 | 0.0405228 | 0.186909 | 0.0873323 | 0.351111 | 0.0369907 | -0.0625858 | 0.201193 | 0.175795 | 0.0762188 | 0.339998 | 0.012654 | -0.0869225 | 0.176856 | 0.314399 | 0.214823 | 0.478602 |
| 93842\_at | Dap | death-associated protein | 223453 | 15 | 6 | apoptosis  induction of apoptosis by extracellular signals |  | 9.68574 | 9.21789 | 9.81746 | 9.95942 | 10.205 | 9.44319 | 9.94877 | 9.7076 | 10.8561 | 10.8841 | 11.4756 | 11.6432 | 11.5065 | 11.0425 | 11.7243 | 11.2848 | 10.3542 | 10.4637 | 10.3027 | -0.668512 | -0.777918 | -0.616959 | -1.13636 | -1.24577 | -1.08481 | -0.536792 | -0.646197 | -0.485238 | -0.394825 | -0.504231 | -0.343272 | -0.149209 | -0.258615 | -0.0976551 | -0.911061 | -1.02047 | -0.859507 | -0.405478 | -0.514884 | -0.353924 | -0.646646 | -0.756052 | -0.595093 | 0.501857 | 0.392451 | 0.553411 | 0.529888 | 0.420482 | 0.581441 | 1.1214 | 1.01199 | 1.17295 | 1.28894 | 1.17953 | 1.34049 | 1.15225 | 1.04284 | 1.2038 | 0.688279 | 0.578873 | 0.739833 | 1.37008 | 1.26068 | 1.42164 | 0.930539 | 0.821133 | 0.982093 |
| 93974\_at | 1300002F13Rik | RIKEN cDNA 1300002F13 gene | 74155 | 4 | 6 |  |  | 3.39229 | 4.18427 | 4.07181 | 3.48519 | 4.28585 | 4.42077 | 4.77184 | 4.84122 | 9.19885 | 8.54989 | 6.77412 | 7.90985 | 5.65152 | 9.58786 | 8.90596 | 9.11314 | 4.08244 | 3.89775 | 3.55346 | -0.690157 | -0.505466 | -0.161168 | 0.101828 | 0.286518 | 0.630816 | -0.010638 | 0.174052 | 0.518351 | -0.59725 | -0.41256 | -0.0682615 | 0.203403 | 0.388094 | 0.732392 | 0.338323 | 0.523014 | 0.867312 | 0.689396 | 0.874086 | 1.21838 | 0.758775 | 0.943465 | 1.28776 | 5.1164 | 5.30109 | 5.64539 | 4.46745 | 4.65214 | 4.99644 | 2.69168 | 2.87637 | 3.22067 | 3.82741 | 4.0121 | 4.3564 | 1.56908 | 1.75377 | 2.09807 | 5.50541 | 5.6901 | 6.0344 | 4.82351 | 5.0082 | 5.3525 | 5.03069 | 5.21538 | 5.55968 |
| 93975\_at | 1300002F13Rik | RIKEN cDNA 1300002F13 gene | 74155 | 4 | 6 |  |  | 5.02062 | 5.54455 | 5.35561 | 6.82747 | 8.47132 | 7.04602 | 7.9189 | 6.97341 | 9.92269 | 9.64253 | 9.74411 | 10.0262 | 9.22414 | 9.74612 | 9.90178 | 9.79776 | 6.56536 | 6.42826 | 6.77421 | -1.54474 | -1.40764 | -1.75359 | -1.02081 | -0.883715 | -1.22966 | -1.20976 | -1.07266 | -1.4186 | 0.262107 | 0.399207 | 0.0532604 | 1.90596 | 2.04306 | 1.69711 | 0.480659 | 0.617758 | 0.271812 | 1.35353 | 1.49063 | 1.14469 | 0.408052 | 0.545152 | 0.199205 | 3.35733 | 3.49443 | 3.14848 | 3.07716 | 3.21426 | 2.86832 | 3.17874 | 3.31584 | 2.9699 | 3.46079 | 3.59789 | 3.25195 | 2.65878 | 2.79588 | 2.44993 | 3.18076 | 3.31786 | 2.97191 | 3.33642 | 3.47352 | 3.12757 | 3.2324 | 3.3695 | 3.02356 |
| 94207\_at | Txndc7 | thioredoxin domain containing 7 | 71853 | 12 | 8 | arsenate reductase (thioredoxin) activity  calcium ion binding  electron transport  endoplasmic reticulum  extracellular space  isomerase activity  protein disulfide isomerase activity |  | 3.36138 | 3.66335 | 3.28125 | 3.51384 | 3.78315 | 3.41849 | 3.68629 | 3.50069 | 4.67204 | 5.37632 | 5.94408 | 6.7228 | 6.04054 | 6.00596 | 5.88867 | 6.33869 | 3.54177 | 3.75269 | 3.6962 | -0.180387 | -0.391306 | -0.33482 | 0.121578 | -0.0893422 | -0.032856 | -0.260515 | -0.471435 | -0.414949 | -0.0279301 | -0.23885 | -0.182364 | 0.241376 | 0.0304563 | 0.0869425 | -0.123278 | -0.334198 | -0.277712 | 0.144518 | -0.066402 | -0.00991577 | -0.0410782 | -0.251998 | -0.195512 | 1.13027 | 0.919348 | 0.975834 | 1.83455 | 1.62363 | 1.68011 | 2.40232 | 2.1914 | 2.24788 | 3.18103 | 2.97011 | 3.0266 | 2.49877 | 2.28785 | 2.34434 | 2.46419 | 2.25327 | 2.30976 | 2.3469 | 2.13598 | 2.19247 | 2.79692 | 2.586 | 2.64249 |
| 94278\_at | Lcp1 | lymphocyte cytosolic protein 1 | 18826 | 14 | 8 | actin binding  calcium ion binding |  | 10.3999 | 10.0237 | 9.93319 | 10.0898 | 10.2385 | 9.8039 | 10.5293 | 10.5 | 10.9202 | 11.2925 | 11.5423 | 11.6239 | 11.205 | 11.199 | 11.5534 | 11.5483 | 9.63781 | 9.53843 | 9.9381 | 0.76209 | 0.861472 | 0.461807 | 0.385836 | 0.485218 | 0.0855533 | 0.295376 | 0.394758 | -0.0049071 | 0.452021 | 0.551403 | 0.151739 | 0.600643 | 0.700025 | 0.30036 | 0.166087 | 0.265469 | -0.134196 | 0.891457 | 0.990839 | 0.591174 | 0.862229 | 0.961611 | 0.561947 | 1.28235 | 1.38174 | 0.98207 | 1.65471 | 1.75409 | 1.35443 | 1.90448 | 2.00386 | 1.60419 | 1.98605 | 2.08544 | 1.68577 | 1.56714 | 1.66652 | 1.26686 | 1.56123 | 1.66061 | 1.26094 | 1.91561 | 2.01499 | 1.61533 | 1.91053 | 2.00992 | 1.61025 |
| 94319\_at | Rab18 | RAB18, member RAS oncogene family | 19330 | 18 | 9 | DNA binding  GTP binding  intracellular  protein transport  regulation of transcription, DNA-dependent  small GTPase mediated signal transduction  two-component signal transduction system (phosphorelay) |  | 10.0716 | 10.5854 | 10.4914 | 10.2954 | 10.3129 | 10.5536 | 10.5115 | 10.2014 | 10.9821 | 11.1248 | 11.6342 | 11.6582 | 11.221 | 11.7371 | 11.6203 | 11.595 | 10.4609 | 11.1373 | 10.3045 | -0.389302 | -1.06572 | -0.232887 | 0.124493 | -0.551922 | 0.280907 | 0.0305029 | -0.645912 | 0.186917 | -0.165453 | -0.841868 | -0.00903908 | -0.147964 | -0.824379 | 0.00844987 | 0.0927259 | -0.583689 | 0.24914 | 0.0506415 | -0.625773 | 0.207056 | -0.259443 | -0.935858 | -0.103029 | 0.521265 | -0.15515 | 0.677679 | 0.663904 | -0.0125104 | 0.820318 | 1.17335 | 0.496935 | 1.32976 | 1.19729 | 0.520878 | 1.35371 | 0.760077 | 0.0836627 | 0.916492 | 1.27623 | 0.599815 | 1.43264 | 1.15943 | 0.483014 | 1.31584 | 1.13409 | 0.457675 | 1.2905 |
| 94322\_at | Sqle | squalene epoxidase | 20775 | 15 | 4 | aromatic compound metabolism  electron transport  extracellular space  integral to membrane  metabolism  monooxygenase activity  oxidoreductase activity  squalene monooxygenase activity | Biosynthesis of steroids  Terpenoid biosynthesis | 2.99286 | 3.0618 | 3.12718 | 2.99021 | 3.8268 | 3.19542 | 3.71392 | 3.28783 | 4.36786 | 5.03266 | 6.46807 | 6.30788 | 5.67766 | 6.48181 | 6.9681 | 6.72078 | 4.25622 | 4.55285 | 4.23589 | -1.26336 | -1.55999 | -1.24303 | -1.19443 | -1.49106 | -1.1741 | -1.12904 | -1.42567 | -1.10871 | -1.26601 | -1.56264 | -1.24568 | -0.429422 | -0.726053 | -0.409089 | -1.0608 | -1.35743 | -1.04047 | -0.542308 | -0.838939 | -0.521975 | -0.96839 | -1.26502 | -0.948057 | 0.111634 | -0.184996 | 0.131967 | 0.776439 | 0.479808 | 0.796772 | 2.21185 | 1.91522 | 2.23218 | 2.05165 | 1.75502 | 2.07199 | 1.42143 | 1.1248 | 1.44177 | 2.22559 | 1.92896 | 2.24592 | 2.71187 | 2.41524 | 2.73221 | 2.46456 | 2.16793 | 2.48489 |
| 94384\_at | Ier3 | immediate early response 3 | 15937 | 17 | 9 | integral to membrane |  | 4.86466 | 4.82937 | 6.98985 | 7.01829 | 8.9689 | 7.82467 | 8.40047 | 7.85421 | 11.3107 | 10.4405 | 11.4447 | 11.8048 | 11.6132 | 11.7922 | 12.1437 | 11.6097 | 3.55086 | 3.53452 | 3.49971 | 1.3138 | 1.33014 | 1.36494 | 1.27852 | 1.29485 | 1.32966 | 3.439 | 3.45533 | 3.49014 | 3.46743 | 3.48377 | 3.51858 | 5.41804 | 5.43438 | 5.46919 | 4.27382 | 4.29015 | 4.32496 | 4.84961 | 4.86595 | 4.90076 | 4.30335 | 4.31968 | 4.35449 | 7.75986 | 7.77619 | 7.811 | 6.88963 | 6.90596 | 6.94077 | 7.89388 | 7.91022 | 7.94503 | 8.25391 | 8.27025 | 8.30506 | 8.06234 | 8.07868 | 8.11349 | 8.24135 | 8.25769 | 8.2925 | 8.59286 | 8.6092 | 8.64401 | 8.05887 | 8.0752 | 8.11001 |
| 94423\_at | BC004728 | cDNA sequence BC004728 | 207818 | 15 | 2 |  |  | 2.81645 | 2.84782 | 3.10133 | 2.88306 | 3.42399 | 3.23609 | 3.20959 | 2.82515 | 4.24611 | 4.71785 | 6.3274 | 6.22147 | 6.39201 | 5.64508 | 7.15737 | 6.99061 | 3.3638 | 3.06219 | 3.35816 | -0.547351 | -0.245749 | -0.541715 | -0.515974 | -0.214372 | -0.510337 | -0.262471 | 0.0391318 | -0.256834 | -0.48074 | -0.179137 | -0.475103 | 0.0601962 | 0.361799 | 0.0658329 | -0.127708 | 0.173894 | -0.122072 | -0.154204 | 0.147399 | -0.148567 | -0.538652 | -0.237049 | -0.533015 | 0.882314 | 1.18392 | 0.887951 | 1.35405 | 1.65565 | 1.35969 | 2.96361 | 3.26521 | 2.96924 | 2.85767 | 3.15928 | 2.86331 | 3.02822 | 3.32982 | 3.03385 | 2.28128 | 2.58288 | 2.28692 | 3.79357 | 4.09518 | 3.79921 | 3.62681 | 3.92841 | 3.63245 |
| 94433\_at | Slc38a2 | solute carrier family 38, member 2 | 67760 | 15 | 8 |  |  | 9.55789 | 9.53583 | 9.45926 | 9.2329 | 9.44411 | 9.70616 | 9.76585 | 10.0256 | 10.159 | 10.1415 | 11.0687 | 10.7465 | 10.4697 | 11.2265 | 11.4492 | 10.9313 | 9.502 | 10.0945 | 9.48348 | 0.0558859 | -0.536614 | 0.074409 | 0.0338307 | -0.558669 | 0.0523539 | -0.042745 | -0.635245 | -0.0242219 | -0.269107 | -0.861607 | -0.250584 | -0.0578928 | -0.650393 | -0.0393697 | 0.204158 | -0.388342 | 0.222681 | 0.263845 | -0.328655 | 0.282368 | 0.523549 | -0.0689508 | 0.542072 | 0.657035 | 0.0645345 | 0.675558 | 0.639522 | 0.0470224 | 0.658046 | 1.56674 | 0.974235 | 1.58526 | 1.24451 | 0.652015 | 1.26304 | 0.967649 | 0.375149 | 0.986172 | 1.72448 | 1.13198 | 1.743 | 1.94722 | 1.35472 | 1.96575 | 1.42931 | 0.836806 | 1.44783 |
| 94536\_s\_at | 2900073G15Rik | RIKEN cDNA 2900073G15 gene | 67268 | 17 | 6 |  |  | 12.1105 | 12.0414 | 11.8616 | 12.0048 | 12.1978 | 12.0698 | 12.2573 | 12.2851 | 13.1186 | 12.646 | 12.6363 | 12.8593 | 12.8916 | 12.9343 | 13.0152 | 12.8379 | 11.5732 | 11.2831 | 11.2295 | 0.537371 | 0.827412 | 0.88103 | 0.468195 | 0.758236 | 0.811854 | 0.288491 | 0.578532 | 0.63215 | 0.431644 | 0.721685 | 0.775303 | 0.624658 | 0.914699 | 0.968317 | 0.496621 | 0.786662 | 0.840281 | 0.684158 | 0.974199 | 1.02782 | 0.711894 | 1.00194 | 1.05555 | 1.54547 | 1.83551 | 1.88913 | 1.07287 | 1.36291 | 1.41653 | 1.06316 | 1.3532 | 1.40682 | 1.28612 | 1.57616 | 1.62977 | 1.31848 | 1.60852 | 1.66214 | 1.36113 | 1.65117 | 1.70479 | 1.44202 | 1.73206 | 1.78568 | 1.26472 | 1.55476 | 1.60838 |
| 94545\_at | Rtn1 | reticulon 1 | 104001 | 12 | 8 | biological\_process unknown  endoplasmic reticulum  molecular\_function unknown |  | 2.88894 | 2.95127 | 2.99276 | 2.88431 | 2.98196 | 2.97194 | 3.0092 | 2.99561 | 6.64409 | 5.65544 | 6.70477 | 6.13483 | 6.43805 | 7.17643 | 7.68883 | 7.44036 | 2.85776 | 2.86118 | 2.819 | 0.0311791 | 0.0277631 | 0.069942 | 0.0935061 | 0.0900902 | 0.132269 | 0.135 | 0.131584 | 0.173763 | 0.0265533 | 0.0231373 | 0.0653162 | 0.124198 | 0.120782 | 0.162961 | 0.114175 | 0.110759 | 0.152938 | 0.151434 | 0.148018 | 0.190197 | 0.13785 | 0.134434 | 0.176613 | 3.78633 | 3.78292 | 3.82509 | 2.79768 | 2.79426 | 2.83644 | 3.84701 | 3.84359 | 3.88577 | 3.27707 | 3.27365 | 3.31583 | 3.58029 | 3.57687 | 3.61905 | 4.31866 | 4.31525 | 4.35743 | 4.83107 | 4.82765 | 4.86983 | 4.5826 | 4.57919 | 4.62136 |
| 94550\_at | Snx1 | sorting nexin 1 | 56440 |  | 7 | Golgi apparatus  intracellular protein transport  intracellular signaling cascade  protein transport  protein transporter activity  transport |  | 8.05275 | 7.49841 | 7.53598 | 6.83984 | 8.05867 | 7.55318 | 8.48462 | 7.72477 | 9.50021 | 9.33835 | 10.059 | 9.45196 | 9.60995 | 9.73844 | 9.65136 | 9.35848 | 6.76448 | 6.72266 | 6.6854 | 1.28827 | 1.33009 | 1.36735 | 0.733928 | 0.775751 | 0.813012 | 0.771497 | 0.81332 | 0.850581 | 0.0753559 | 0.117179 | 0.15444 | 1.29419 | 1.33601 | 1.37327 | 0.788696 | 0.830519 | 0.86778 | 1.72014 | 1.76196 | 1.79922 | 0.960287 | 1.00211 | 1.03937 | 2.73573 | 2.77755 | 2.81481 | 2.57387 | 2.6157 | 2.65296 | 3.29451 | 3.33633 | 3.37359 | 2.68748 | 2.7293 | 2.76656 | 2.84547 | 2.88729 | 2.92456 | 2.97396 | 3.01578 | 3.05304 | 2.88688 | 2.9287 | 2.96596 | 2.594 | 2.63582 | 2.67308 |
| 94713\_at | Myo7a | myosin VIIa | 17921 | 7 | 7 | ATP binding  actin binding  calmodulin binding  cytoskeleton  cytoskeleton organization and biogenesis  motor activity  myosin  perception of sound  protein binding  signal transduction |  | 2.08577 | 2.16023 | 2.20331 | 2.10246 | 2.12843 | 2.17724 | 2.15636 | 2.17382 | 4.06968 | 6.99321 | 7.92213 | 8.24061 | 7.95624 | 8.31477 | 8.37506 | 9.02693 | 2.0764 | 2.07998 | 2.05505 | 0.00936888 | 0.00579521 | 0.0307211 | 0.0838245 | 0.0802508 | 0.105177 | 0.126909 | 0.123335 | 0.148261 | 0.0260607 | 0.022487 | 0.0474129 | 0.0520327 | 0.0484591 | 0.0733849 | 0.100834 | 0.0972601 | 0.122186 | 0.0799545 | 0.0763809 | 0.101307 | 0.0974219 | 0.0938483 | 0.118774 | 1.99328 | 1.9897 | 2.01463 | 4.91681 | 4.91323 | 4.93816 | 5.84573 | 5.84215 | 5.86708 | 6.16421 | 6.16064 | 6.18556 | 5.87984 | 5.87626 | 5.90119 | 6.23837 | 6.23479 | 6.25972 | 6.29866 | 6.29509 | 6.32001 | 6.95052 | 6.94695 | 6.97188 |
| 94834\_at | Ctsh | cathepsin H | 13036 | 9 | 9 | cathepsin H activity  cysteine-type endopeptidase activity  cysteine-type peptidase activity  extracellular space  hydrolase activity  lysosome  peptidase activity  proteolysis and peptidolysis |  | 9.913 | 9.99514 | 9.58874 | 10.0582 | 10.5301 | 9.66285 | 10.2623 | 10.0284 | 11.3924 | 11.2051 | 11.7795 | 11.5479 | 11.8819 | 11.8386 | 11.7947 | 11.5175 | 10.7939 | 11.0684 | 10.7631 | -0.880915 | -1.15541 | -0.850063 | -0.798773 | -1.07327 | -0.767921 | -1.20518 | -1.47968 | -1.17433 | -0.735753 | -1.01025 | -0.704901 | -0.263858 | -0.538358 | -0.233006 | -1.13107 | -1.40557 | -1.10021 | -0.531608 | -0.806108 | -0.500756 | -0.765486 | -1.03999 | -0.734634 | 0.598465 | 0.323965 | 0.629317 | 0.411143 | 0.136643 | 0.441995 | 0.985572 | 0.711072 | 1.01642 | 0.754007 | 0.479507 | 0.784859 | 1.08794 | 0.813439 | 1.11879 | 1.04468 | 0.770176 | 1.07553 | 1.00076 | 0.726258 | 1.03161 | 0.723547 | 0.449047 | 0.754399 |
| 94872\_at | Smpdl3a | sphingomyelin phosphodiesterase, acid-like 3A | 57319 | 10 | 5 | carbohydrate metabolism  extracellular space  hydrolase activity  hydrolase activity, acting on glycosyl bonds | Aminosugars metabolism | 9.55631 | 9.52324 | 10.0485 | 9.86627 | 10.1741 | 10.1153 | 10.1582 | 10.12 | 10.216 | 10.7308 | 10.9694 | 10.6457 | 10.5275 | 11.0642 | 10.8788 | 10.9618 | 9.25856 | 9.06453 | 8.91061 | 0.297747 | 0.491782 | 0.6457 | 0.264682 | 0.458717 | 0.612634 | 0.789934 | 0.983968 | 1.13789 | 0.607712 | 0.801747 | 0.955664 | 0.915515 | 1.10955 | 1.26347 | 0.856745 | 1.05078 | 1.2047 | 0.899618 | 1.09365 | 1.24757 | 0.861416 | 1.05545 | 1.20937 | 0.957428 | 1.15146 | 1.30538 | 1.47226 | 1.6663 | 1.82021 | 1.71086 | 1.90489 | 2.05881 | 1.38717 | 1.58121 | 1.73512 | 1.26893 | 1.46296 | 1.61688 | 1.80568 | 1.99971 | 2.15363 | 1.6202 | 1.81424 | 1.96815 | 1.70321 | 1.89724 | 2.05116 |
| 94876\_f\_at | Gorasp2 | golgi reassembly stacking protein 2 | 70231 | 2 | 7 | Golgi apparatus  biological\_process unknown  membrane  protein binding |  | 9.84239 | 9.77332 | 9.72681 | 9.58625 | 10.1447 | 10.0523 | 10.4133 | 10.087 | 11.1392 | 11.1789 | 11.6076 | 11.2629 | 11.4582 | 11.6354 | 11.7706 | 11.7154 | 10.2378 | 10.5375 | 10.6515 | -0.395382 | -0.695071 | -0.809099 | -0.464456 | -0.764144 | -0.878172 | -0.510969 | -0.810658 | -0.924685 | -0.651525 | -0.951213 | -1.06524 | -0.0930812 | -0.39277 | -0.506797 | -0.185435 | -0.485123 | -0.599151 | 0.175493 | -0.124195 | -0.238223 | -0.150758 | -0.450447 | -0.564475 | 0.901399 | 0.601711 | 0.487683 | 0.941151 | 0.641462 | 0.527434 | 1.36981 | 1.07012 | 0.956089 | 1.02508 | 0.725394 | 0.611366 | 1.22037 | 0.920686 | 0.806658 | 1.39766 | 1.09797 | 0.983946 | 1.53285 | 1.23317 | 1.11914 | 1.47757 | 1.17789 | 1.06386 |
| 95022\_at | Akap12 | A kinase (PRKA) anchor protein (gravin) 12 | 83397 | 10 | 14 | cellular\_component unknown  kinase activity  protein targeting  protein transporter activity  receptor signaling complex scaffold activity  signal transduction |  | 5.25506 | 5.37105 | 5.84513 | 5.63772 | 6.03734 | 5.79022 | 5.7238 | 5.57753 | 8.05088 | 8.40152 | 9.58174 | 9.31361 | 9.71813 | 9.48466 | 9.88556 | 8.70874 | 5.0545 | 5.04141 | 5.08471 | 0.200567 | 0.213654 | 0.170351 | 0.316553 | 0.32964 | 0.286336 | 0.790631 | 0.803718 | 0.760415 | 0.583224 | 0.596311 | 0.553007 | 0.982843 | 0.99593 | 0.952626 | 0.735724 | 0.748811 | 0.705508 | 0.669303 | 0.68239 | 0.639086 | 0.523033 | 0.53612 | 0.492817 | 2.99638 | 3.00947 | 2.96616 | 3.34703 | 3.36011 | 3.31681 | 4.52725 | 4.54033 | 4.49703 | 4.25912 | 4.27221 | 4.2289 | 4.66364 | 4.67672 | 4.63342 | 4.43016 | 4.44325 | 4.39994 | 4.83106 | 4.84415 | 4.80085 | 3.65425 | 3.66733 | 3.62403 |
| 95286\_at | Clu | clusterin | 12759 | 14 | 10 | cell death  extracellular space |  | 9.72463 | 9.26276 | 9.73065 | 9.74909 | 9.92537 | 9.59013 | 9.85466 | 9.81166 | 10.6513 | 10.9149 | 11.2163 | 11.4997 | 11.3935 | 10.9517 | 10.9835 | 11.5764 | 11.5964 | 11.9563 | 11.6061 | -1.87173 | -2.23168 | -1.88146 | -2.33359 | -2.69355 | -2.34332 | -1.86571 | -2.22567 | -1.87544 | -1.84727 | -2.20722 | -1.857 | -1.67098 | -2.03094 | -1.68071 | -2.00623 | -2.36619 | -2.01596 | -1.7417 | -2.10165 | -1.75143 | -1.7847 | -2.14465 | -1.79443 | -0.945041 | -1.30499 | -0.954771 | -0.681449 | -1.0414 | -0.691179 | -0.380043 | -0.739995 | -0.389773 | -0.0966207 | -0.456573 | -0.106351 | -0.202851 | -0.562803 | -0.212581 | -0.644682 | -1.00463 | -0.654412 | -0.61289 | -0.972843 | -0.622621 | -0.0200025 | -0.379955 | -0.0297325 |
| 95465\_s\_at | Cacng6 | calcium channel, voltage-dependent, gamma subunit 6 | 54378 | 7 | 4 | calcium channel activity  calcium ion transport  integral to membrane  ion channel activity  ion transport  membrane  structural molecule activity  tight junction  voltage-gated ion channel activity |  | 1.46689 | 1.52748 | 1.55968 | 1.46183 | 1.51101 | 1.54284 | 1.49352 | 1.5349 | 2.63208 | 5.75369 | 6.99709 | 7.5235 | 7.55076 | 7.40855 | 8.11767 | 8.01724 | 1.42917 | 1.4417 | 1.39983 | 0.0377179 | 0.0251859 | 0.0670551 | 0.0983071 | 0.0857751 | 0.127644 | 0.130514 | 0.117981 | 0.159851 | 0.0326637 | 0.0201317 | 0.0620009 | 0.0818384 | 0.0693064 | 0.111176 | 0.113671 | 0.101139 | 0.143008 | 0.0643481 | 0.051816 | 0.0936852 | 0.105732 | 0.0931997 | 0.135069 | 1.20291 | 1.19038 | 1.23224 | 4.32452 | 4.31198 | 4.35385 | 5.56792 | 5.55539 | 5.59726 | 6.09433 | 6.0818 | 6.12367 | 6.12159 | 6.10906 | 6.15093 | 5.97938 | 5.96685 | 6.00872 | 6.6885 | 6.67597 | 6.71783 | 6.58807 | 6.57553 | 6.6174 |
| 95480\_at |  |  | 68583 |  |  |  |  | 6.97017 | 6.30087 | 6.82091 | 6.64977 | 6.71748 | 6.89306 | 6.84486 | 6.85978 | 7.32703 | 7.42627 | 8.42402 | 8.09556 | 8.43271 | 7.91735 | 8.532 | 7.65935 | 6.7879 | 7.03679 | 7.0953 | 0.182271 | -0.0666265 | -0.12513 | -0.487031 | -0.735928 | -0.794432 | 0.0330157 | -0.215881 | -0.274385 | -0.138131 | -0.387028 | -0.445531 | -0.0704188 | -0.319316 | -0.377819 | 0.105166 | -0.143731 | -0.202235 | 0.0569656 | -0.191931 | -0.250435 | 0.0718845 | -0.177013 | -0.235516 | 0.539128 | 0.290231 | 0.231728 | 0.638376 | 0.389479 | 0.330975 | 1.63612 | 1.38723 | 1.32872 | 1.30766 | 1.05876 | 1.00026 | 1.64481 | 1.39592 | 1.33741 | 1.12945 | 0.880554 | 0.82205 | 1.7441 | 1.4952 | 1.4367 | 0.871448 | 0.622551 | 0.564047 |
| 95518\_at | 1810015C04Rik | RIKEN cDNA 1810015C04 gene | 66270 | 15 | 6 | integral to membrane |  | 8.76506 | 9.68932 | 8.88357 | 8.94474 | 9.51702 | 9.59998 | 9.69781 | 9.66573 | 10.2699 | 10.7463 | 10.9336 | 11.0248 | 10.6866 | 11.1256 | 11.5997 | 11.2571 | 10.5021 | 10.6812 | 10.0516 | -1.73707 | -1.91614 | -1.28652 | -0.812819 | -0.991889 | -0.362263 | -1.61856 | -1.79763 | -1.16801 | -1.5574 | -1.73647 | -1.10685 | -0.985118 | -1.16419 | -0.534562 | -0.902159 | -1.08123 | -0.451603 | -0.804332 | -0.983402 | -0.353776 | -0.836402 | -1.01547 | -0.385846 | -0.232268 | -0.411339 | 0.218288 | 0.244183 | 0.0651121 | 0.694739 | 0.431441 | 0.252371 | 0.881997 | 0.522679 | 0.343608 | 0.973235 | 0.184487 | 0.00541644 | 0.635043 | 0.62351 | 0.44444 | 1.07407 | 1.09759 | 0.918518 | 1.54814 | 0.754956 | 0.575885 | 1.20551 |
| 95541\_at | D6Wsu176e | DNA segment, Chr 6, Wayne State University 176, expressed | 27999 | 6 | 7 | extracellular space |  | 6.50141 | 7.12999 | 7.61304 | 7.62441 | 9.31934 | 7.98343 | 9.11629 | 8.95146 | 10.5659 | 11.1748 | 11.4206 | 11.3424 | 10.9109 | 11.3713 | 11.2967 | 11.8349 | 10.8377 | 11.1357 | 10.2766 | -4.33633 | -4.63427 | -3.77514 | -3.70776 | -4.0057 | -3.14657 | -3.22471 | -3.52265 | -2.66351 | -3.21333 | -3.51127 | -2.65214 | -1.5184 | -1.81635 | -0.957212 | -2.85431 | -3.15226 | -2.29312 | -1.72146 | -2.0194 | -1.16026 | -1.88629 | -2.18423 | -1.3251 | -0.271872 | -0.569814 | 0.28932 | 0.337058 | 0.0391161 | 0.898249 | 0.582849 | 0.284908 | 1.14404 | 0.504622 | 0.206681 | 1.06581 | 0.0731132 | -0.224828 | 0.634305 | 0.533552 | 0.23561 | 1.09474 | 0.45891 | 0.160968 | 1.0201 | 0.997112 | 0.699171 | 1.5583 |
| 95619\_at | 1700040I03Rik | RIKEN cDNA 1700040I03 gene | 73327 | 6 | 6 | extracellular space  peptidase activity |  | 4.68754 | 4.68546 | 4.71365 | 4.1127 | 5.14727 | 4.78165 | 5.01055 | 4.67066 | 5.48938 | 7.08912 | 7.20907 | 6.96555 | 6.85215 | 7.64354 | 7.99374 | 6.83294 | 7.03003 | 7.98634 | 8.46694 | -2.34248 | -3.2988 | -3.7794 | -2.34456 | -3.30088 | -3.78148 | -2.31637 | -3.27268 | -3.75329 | -2.91732 | -3.87363 | -4.35424 | -1.88275 | -2.83906 | -3.31967 | -2.24838 | -3.20469 | -3.6853 | -2.01947 | -2.97578 | -3.45639 | -2.35937 | -3.31568 | -3.79629 | -1.54065 | -2.49696 | -2.97757 | 0.0590927 | -0.897219 | -1.37782 | 0.179044 | -0.777268 | -1.25787 | -0.064475 | -1.02079 | -1.50139 | -0.177878 | -1.13419 | -1.61479 | 0.61351 | -0.342802 | -0.823406 | 0.963709 | 0.0073968 | -0.473207 | -0.197087 | -1.1534 | -1.634 |
| 95637\_at | Flnb | filamin, beta | 286940 | 14 | 7 | actin binding  cytoplasm  cytoskeleton  focal adhesion  myogenesis  protein binding  stress fiber | MAPK signaling pathway | 6.34134 | 7.34169 | 7.85633 | 7.49076 | 8.26994 | 7.90454 | 7.88753 | 7.09571 | 9.75977 | 9.61417 | 10.9608 | 10.4459 | 10.6819 | 10.7189 | 10.138 | 10.4292 | 8.90693 | 8.57111 | 7.92661 | -2.56558 | -2.22977 | -1.58527 | -1.56524 | -1.22943 | -0.584926 | -1.05059 | -0.714779 | -0.0702783 | -1.41616 | -1.08035 | -0.435848 | -0.636983 | -0.301168 | 0.343332 | -1.00239 | -0.666576 | -0.022076 | -1.01939 | -0.683579 | -0.0390784 | -1.81122 | -1.4754 | -0.830901 | 0.852842 | 1.18866 | 1.83316 | 0.707248 | 1.04306 | 1.68756 | 2.05392 | 2.38973 | 3.03423 | 1.53895 | 1.87477 | 2.51927 | 1.77493 | 2.11075 | 2.75525 | 1.81196 | 2.14777 | 2.79227 | 1.23107 | 1.56689 | 2.21139 | 1.52226 | 1.85808 | 2.50258 |
| 95671\_at | Hey1 | hairy/enhancer-of-split related with YRPW motif 1 | 15213 | 3 | 11 | DNA binding  nucleus  regulation of transcription, DNA-dependent  transcription factor activity  vasculogenesis |  | 3.89331 | 3.7284 | 5.23116 | 4.91206 | 6.06799 | 5.10926 | 5.58102 | 5.0133 | 6.24582 | 5.94446 | 7.3994 | 7.48754 | 7.29901 | 7.37471 | 7.35308 | 7.49742 | 2.59711 | 1.89686 | 1.8495 | 1.29619 | 1.99645 | 2.0438 | 1.13129 | 1.83155 | 1.8789 | 2.63405 | 3.33431 | 3.38166 | 2.31494 | 3.0152 | 3.06255 | 3.47088 | 4.17113 | 4.21849 | 2.51215 | 3.2124 | 3.25976 | 2.9839 | 3.68416 | 3.73151 | 2.41619 | 3.11644 | 3.1638 | 3.64871 | 4.34896 | 4.39631 | 3.34734 | 4.0476 | 4.09495 | 4.80229 | 5.50255 | 5.5499 | 4.89043 | 5.59068 | 5.63804 | 4.7019 | 5.40215 | 5.44951 | 4.7776 | 5.47786 | 5.52521 | 4.75597 | 5.45622 | 5.50357 | 4.9003 | 5.60056 | 5.64791 |
| 95708\_at | D3Ucla1 | DNA segment, Chr 3, University of California at Los Angeles 1 | 28146 | 3 | 7 |  |  | 10.1324 | 10.1945 | 10.0181 | 10.0779 | 10.5117 | 10.2303 | 10.524 | 10.3369 | 10.8386 | 11.1176 | 11.2832 | 11.3422 | 10.9339 | 11.2734 | 11.4739 | 11.4531 | 11.3203 | 11.667 | 11.1304 | -1.18792 | -1.53468 | -0.998048 | -1.12576 | -1.47252 | -0.935887 | -1.30222 | -1.64899 | -1.11235 | -1.24242 | -1.58918 | -1.05254 | -0.808563 | -1.15532 | -0.618691 | -1.08998 | -1.43674 | -0.900108 | -0.796322 | -1.14308 | -0.60645 | -0.98341 | -1.33017 | -0.793538 | -0.481705 | -0.828467 | -0.291833 | -0.202708 | -0.54947 | -0.0128363 | -0.0371039 | -0.383865 | 0.152768 | 0.0219337 | -0.324828 | 0.211806 | -0.386361 | -0.733123 | -0.196489 | -0.0468894 | -0.393651 | 0.142983 | 0.153586 | -0.193176 | 0.343458 | 0.13281 | -0.213951 | 0.322682 |
| 95722\_at | Glrx1 | glutaredoxin 1 (thioltransferase) | 93692 | 13 | 6 | electron transport  electron transporter activity  protein-disulfide reductase (glutathione) activity |  | 3.50861 | 3.60318 | 3.28639 | 3.37468 | 3.56917 | 3.55615 | 3.52776 | 3.29734 | 4.55828 | 6.17514 | 7.12156 | 7.46121 | 6.30682 | 6.88735 | 8.41907 | 5.77266 | 3.52629 | 3.54722 | 3.44891 | -0.0176772 | -0.0386078 | 0.0597024 | 0.0768909 | 0.0559603 | 0.15427 | -0.239898 | -0.260829 | -0.162519 | -0.151608 | -0.172539 | -0.0742286 | 0.0428845 | 0.0219538 | 0.120264 | 0.0298656 | 0.00893496 | 0.107245 | 0.00147139 | -0.0194592 | 0.078851 | -0.228945 | -0.249875 | -0.151565 | 1.03199 | 1.01106 | 1.10937 | 2.64885 | 2.62792 | 2.72623 | 3.59527 | 3.57434 | 3.67265 | 3.93492 | 3.91399 | 4.0123 | 2.78053 | 2.7596 | 2.85791 | 3.36107 | 3.34013 | 3.43845 | 4.89279 | 4.87186 | 4.97017 | 2.24637 | 2.22544 | 2.32375 |
| 95733\_at | Slc29a1 | solute carrier family 29 (nucleoside transporters), member 1 | 63959 | 17 | 10 | integral to membrane  integral to plasma membrane  membrane  nucleoside transport  nucleoside transporter activity  nucleoside transporter activity  transport |  | 7.30878 | 7.76669 | 7.89828 | 7.71207 | 9.2425 | 8.91118 | 9.11429 | 8.58328 | 11.2792 | 11.4105 | 11.8443 | 12.0613 | 11.8197 | 11.8273 | 11.9933 | 12.1064 | 7.69138 | 7.69278 | 7.68727 | -0.382606 | -0.384007 | -0.378496 | 0.0753055 | 0.0739038 | 0.0794154 | 0.206904 | 0.205502 | 0.211014 | 0.0206896 | 0.0192879 | 0.0247995 | 1.55112 | 1.54972 | 1.55523 | 1.2198 | 1.21839 | 1.22391 | 1.42291 | 1.42151 | 1.42702 | 0.891895 | 0.890494 | 0.896005 | 3.58784 | 3.58644 | 3.59195 | 3.71908 | 3.71768 | 3.72319 | 4.15294 | 4.15154 | 4.15705 | 4.36992 | 4.36851 | 4.37403 | 4.12832 | 4.12692 | 4.13243 | 4.13596 | 4.13456 | 4.14007 | 4.30195 | 4.30055 | 4.30606 | 4.41506 | 4.41366 | 4.41917 |
| 95746\_at | Atp6v1a1 | ATPase, H+ transporting, V1 subunit A, isoform 1 | 11964 | 16 | 5 | ATP binding  ATP biosynthesis  ATP synthesis coupled proton transport  hydrogen ion transporter activity  hydrogen-exporting ATPase activity, phosphorylative mechanism  hydrogen-transporting ATP synthase activity, rotational mechanism  hydrogen-transporting ATPase activity, rotational mechanism  hydrolase activity  ion transport  mitochondrion  proton transport  proton-transporting ATP synthase complex (sensu Eukarya)  proton-transporting two-sector ATPase complex  transport | Oxidative phosphorylation  ATP synthesis | 10.041 | 9.52738 | 9.89022 | 9.86808 | 10.282 | 10.4142 | 10.4857 | 10.0747 | 11.1558 | 11.131 | 11.4204 | 11.6434 | 11.2107 | 11.3665 | 11.8086 | 11.7619 | 10.1344 | 10.0635 | 9.69243 | -0.093418 | -0.0225202 | 0.348533 | -0.607001 | -0.536103 | -0.16505 | -0.244156 | -0.173258 | 0.197795 | -0.266296 | -0.195398 | 0.175655 | 0.14764 | 0.218538 | 0.58959 | 0.27978 | 0.350677 | 0.72173 | 0.351276 | 0.422173 | 0.793226 | -0.0596535 | 0.0112443 | 0.382297 | 1.0214 | 1.0923 | 1.46336 | 0.996643 | 1.06754 | 1.43859 | 1.28607 | 1.35697 | 1.72802 | 1.50905 | 1.57994 | 1.951 | 1.07628 | 1.14718 | 1.51823 | 1.23209 | 1.30299 | 1.67404 | 1.67423 | 1.74513 | 2.11618 | 1.62748 | 1.69838 | 2.06943 |
| 96066\_s\_at | Pkm2 | pyruvate kinase, muscle | 18746 | 9 | 10 | glycolysis  kinase activity  magnesium ion binding  mitochondrion  pyruvate kinase activity  transferase activity | Glycolysis / Gluconeogenesis  Purine metabolism  Pyruvate metabolism  Carbon fixation | 11.7296 | 11.6352 | 11.7143 | 11.7948 | 11.8615 | 12.0348 | 11.5577 | 11.7741 | 12.3372 | 12.1778 | 12.7873 | 12.8175 | 12.8465 | 12.783 | 12.6892 | 12.9215 | 11.3943 | 11.4829 | 11.2564 | 0.335385 | 0.246721 | 0.473214 | 0.240943 | 0.152279 | 0.378772 | 0.320044 | 0.23138 | 0.457873 | 0.400573 | 0.311909 | 0.538402 | 0.467236 | 0.378571 | 0.605064 | 0.640539 | 0.551874 | 0.778367 | 0.163423 | 0.0747582 | 0.301251 | 0.379881 | 0.291217 | 0.51771 | 0.942901 | 0.854237 | 1.08073 | 0.783559 | 0.694894 | 0.921387 | 1.39299 | 1.30433 | 1.53082 | 1.42324 | 1.33458 | 1.56107 | 1.45225 | 1.36358 | 1.59008 | 1.38875 | 1.30008 | 1.52658 | 1.29497 | 1.20631 | 1.4328 | 1.52725 | 1.43858 | 1.66508 |
| 96089\_at | 4931406C07Rik | RIKEN cDNA 4931406C07 gene | 70984 | 9 | 6 |  |  | 7.80718 | 7.09746 | 7.66977 | 7.63615 | 7.93348 | 7.55597 | 7.76908 | 7.45857 | 8.58099 | 9.16364 | 9.17729 | 9.36861 | 8.96514 | 9.74679 | 10.0429 | 10.1853 | 6.90765 | 6.70695 | 6.27729 | 0.899531 | 1.10023 | 1.52989 | 0.189818 | 0.390513 | 0.820176 | 0.762124 | 0.962819 | 1.39248 | 0.728509 | 0.929204 | 1.35887 | 1.02583 | 1.22653 | 1.65619 | 0.648324 | 0.849019 | 1.27868 | 0.861438 | 1.06213 | 1.4918 | 0.550922 | 0.751617 | 1.18128 | 1.67334 | 1.87404 | 2.3037 | 2.256 | 2.45669 | 2.88635 | 2.26965 | 2.47034 | 2.9 | 2.46096 | 2.66166 | 3.09132 | 2.05749 | 2.25819 | 2.68785 | 2.83914 | 3.03984 | 3.4695 | 3.13521 | 3.3359 | 3.76556 | 3.27762 | 3.47831 | 3.90798 |
| 96090\_g\_at | 4931406C07Rik | RIKEN cDNA 4931406C07 gene | 70984 | 9 | 6 |  |  | 10.2868 | 9.9982 | 10.5991 | 10.1394 | 10.8012 | 10.3219 | 10.2416 | 10.0341 | 11.1806 | 11.8346 | 11.9253 | 12.0095 | 11.5421 | 11.9944 | 12.2937 | 12.6559 | 7.98696 | 9.07013 | 8.35768 | 2.2998 | 1.21663 | 1.92908 | 2.01124 | 0.928067 | 1.64052 | 2.61218 | 1.52901 | 2.24146 | 2.15248 | 1.06931 | 1.78176 | 2.81423 | 1.73106 | 2.44351 | 2.33489 | 1.25172 | 1.96417 | 2.25462 | 1.17145 | 1.8839 | 2.04711 | 0.96394 | 1.67639 | 3.19362 | 2.11045 | 2.8229 | 3.84761 | 2.76444 | 3.47689 | 3.93836 | 2.85519 | 3.56764 | 4.02252 | 2.93935 | 3.6518 | 3.55515 | 2.47198 | 3.18443 | 4.00746 | 2.92429 | 3.63674 | 4.30671 | 3.22354 | 3.93599 | 4.66893 | 3.58575 | 4.29821 |
| 96123\_at | Lbp | lipopolysaccharide binding protein | 16803 | 2 | 12 | extracellular space  integral to membrane  lipid binding  lipid transport  lipopolysaccharide binding  xenobiotic metabolism | Toll-like receptor signaling pathway | 1.90901 | 2.05828 | 2.1834 | 2.94469 | 6.9342 | 2.68274 | 6.41346 | 2.52889 | 8.71966 | 9.04864 | 10.143 | 10.2977 | 10.1238 | 9.66444 | 9.47406 | 9.68175 | 5.41812 | 5.8669 | 6.34509 | -3.50911 | -3.95789 | -4.43608 | -3.35984 | -3.80863 | -4.28681 | -3.23472 | -3.6835 | -4.16169 | -2.47343 | -2.92221 | -3.4004 | 1.51608 | 1.06729 | 0.589108 | -2.73538 | -3.18416 | -3.66235 | 0.995339 | 0.546553 | 0.0683685 | -2.88923 | -3.33802 | -3.8162 | 3.30154 | 2.85275 | 2.37457 | 3.63052 | 3.18173 | 2.70355 | 4.72485 | 4.27606 | 3.79788 | 4.87958 | 4.4308 | 3.95261 | 4.70572 | 4.25693 | 3.77875 | 4.24632 | 3.79753 | 3.31935 | 4.05594 | 3.60715 | 3.12897 | 4.26363 | 3.81484 | 3.33666 |
| 96343\_at | Actn4 | actinin alpha 4 | 60595 | 7 | 12 | actin binding  calcium ion binding  cortical cytoskeleton  cytoplasm  protein binding |  | 9.52131 | 9.75062 | 10.5824 | 10.5483 | 11.5654 | 10.581 | 10.7099 | 10.2821 | 11.8034 | 12.2177 | 12.7222 | 12.3886 | 12.4487 | 12.6776 | 12.7162 | 12.6915 | 10.5295 | 10.8273 | 11.0911 | -1.00818 | -1.30599 | -1.56978 | -0.778864 | -1.07668 | -1.34046 | 0.0529605 | -0.244852 | -0.508639 | 0.0188234 | -0.278989 | -0.542776 | 1.03596 | 0.738149 | 0.474362 | 0.0515632 | -0.246249 | -0.510036 | 0.180455 | -0.117358 | -0.381145 | -0.247431 | -0.545244 | -0.809031 | 1.27392 | 0.976104 | 0.712317 | 1.6882 | 1.39039 | 1.1266 | 2.19271 | 1.8949 | 1.63111 | 1.85915 | 1.56134 | 1.29755 | 1.91924 | 1.62143 | 1.35764 | 2.14813 | 1.85032 | 1.58653 | 2.18674 | 1.88893 | 1.62514 | 2.16205 | 1.86424 | 1.60045 |
| 96605\_at | 0610011I04Rik | RIKEN cDNA 0610011I04 gene | 66058 | 6 | 6 | integral to membrane |  | 7.01613 | 8.57144 | 8.15771 | 8.53638 | 9.11493 | 8.26702 | 8.52044 | 7.85819 | 9.65151 | 9.46495 | 9.60672 | 9.94529 | 10.2332 | 10.4401 | 10.7912 | 11.5032 | 11.6017 | 11.5812 | 11.5264 | -4.58557 | -4.5651 | -4.51024 | -3.03026 | -3.00979 | -2.95493 | -3.44398 | -3.42351 | -3.36866 | -3.06532 | -3.04485 | -2.99 | -2.48677 | -2.4663 | -2.41144 | -3.33468 | -3.31421 | -3.25936 | -3.08126 | -3.06079 | -3.00594 | -3.74351 | -3.72304 | -3.66819 | -1.95019 | -1.92972 | -1.87487 | -2.13675 | -2.11628 | -2.06143 | -1.99497 | -1.97451 | -1.91965 | -1.65641 | -1.63594 | -1.58108 | -1.3685 | -1.34803 | -1.29318 | -1.16162 | -1.14115 | -1.08629 | -0.810485 | -0.790016 | -0.735161 | -0.0984831 | -0.0780146 | -0.0231599 |
| 96657\_at | Sat1 | spermidine/spermine N1-acetyl transferase 1 | 20229 | X | 10 | N-acetyltransferase activity  acyltransferase activity  diamine N-acetyltransferase activity  spermine catabolism  transferase activity | Arginine and proline metabolism | 9.74159 | 9.57703 | 9.88228 | 9.74044 | 9.96639 | 9.91304 | 10.5758 | 9.87653 | 10.8236 | 10.638 | 10.926 | 11.1616 | 11.0971 | 10.9573 | 11.423 | 10.7088 | 9.85552 | 10.0295 | 9.40713 | -0.113927 | -0.287874 | 0.334459 | -0.278485 | -0.452432 | 0.169901 | 0.0267668 | -0.14718 | 0.475153 | -0.115072 | -0.289019 | 0.333314 | 0.11087 | -0.0630772 | 0.559256 | 0.0575264 | -0.11642 | 0.505912 | 0.720327 | 0.54638 | 1.16871 | 0.0210098 | -0.152937 | 0.469396 | 0.968124 | 0.794177 | 1.41651 | 0.782438 | 0.608491 | 1.23082 | 1.07048 | 0.896529 | 1.51886 | 1.30607 | 1.13212 | 1.75446 | 1.24158 | 1.06763 | 1.68996 | 1.10179 | 0.92784 | 1.55017 | 1.56748 | 1.39353 | 2.01586 | 0.853265 | 0.679318 | 1.30165 |
| 96707\_at | Zipro1 | zinc finger proliferation 1 | 22697 | 5 | 4 | DNA binding  development  nucleic acid binding  nucleus  regulation of transcription, DNA-dependent  spermatogenesis  transcription factor activity  zinc ion binding |  | 5.56265 | 5.17214 | 6.20558 | 6.45735 | 6.77967 | 5.68877 | 6.86482 | 5.73233 | 7.35574 | 8.35257 | 8.72617 | 8.90335 | 8.48655 | 8.18336 | 8.3823 | 8.5362 | 7.30398 | 7.58462 | 7.44566 | -1.74133 | -2.02197 | -1.88302 | -2.13184 | -2.41248 | -2.27353 | -1.0984 | -1.37904 | -1.24008 | -0.846625 | -1.12727 | -0.988311 | -0.524312 | -0.804956 | -0.665999 | -1.6152 | -1.89585 | -1.75689 | -0.439156 | -0.7198 | -0.580842 | -1.57165 | -1.8523 | -1.71334 | 0.0517669 | -0.228877 | -0.0899198 | 1.04859 | 0.767944 | 0.906902 | 1.42219 | 1.14155 | 1.2805 | 1.59938 | 1.31873 | 1.45769 | 1.18257 | 0.901924 | 1.04088 | 0.879382 | 0.598738 | 0.737696 | 1.07832 | 0.797675 | 0.936632 | 1.23222 | 0.951579 | 1.09054 |
| 96709\_at | 1110008P14Rik | RIKEN cDNA 1110008P14 gene | 73737 | 2 | 5 |  |  | 8.51394 | 8.13714 | 8.40131 | 8.43809 | 8.90552 | 9.03329 | 7.85548 | 7.74577 | 10.1385 | 10.2862 | 10.4067 | 10.4472 | 10.9207 | 10.5644 | 10.8502 | 10.7776 | 9.81847 | 10.1531 | 9.88598 | -1.30454 | -1.6392 | -1.37204 | -1.68134 | -2.01601 | -1.74884 | -1.41716 | -1.75183 | -1.48467 | -1.38038 | -1.71505 | -1.44789 | -0.912954 | -1.24762 | -0.980459 | -0.785184 | -1.11985 | -0.852688 | -1.96299 | -2.29766 | -2.0305 | -2.0727 | -2.40737 | -2.14021 | 0.320038 | -0.01463 | 0.252533 | 0.467704 | 0.133036 | 0.400199 | 0.588231 | 0.253563 | 0.520727 | 0.628772 | 0.294104 | 0.561268 | 1.10222 | 0.767553 | 1.03472 | 0.745961 | 0.411293 | 0.678457 | 1.03174 | 0.697068 | 0.964232 | 0.959133 | 0.624465 | 0.891628 |
| 96724\_r\_at |  |  | 97841 |  |  |  |  | 5.55979 | 6.19133 | 6.00454 | 5.54531 | 6.12118 | 6.2014 | 6.00797 | 6.096 | 7.53436 | 6.83312 | 7.5369 | 7.09079 | 7.48773 | 8.39734 | 7.38947 | 8.02098 | 5.66509 | 5.67955 | 5.31359 | -0.105295 | -0.119753 | 0.246197 | 0.526246 | 0.511787 | 0.877738 | 0.33945 | 0.324991 | 0.690942 | -0.119779 | -0.134237 | 0.231713 | 0.456089 | 0.44163 | 0.807581 | 0.536309 | 0.52185 | 0.887801 | 0.34288 | 0.328421 | 0.694372 | 0.430909 | 0.41645 | 0.782401 | 1.86928 | 1.85482 | 2.22077 | 1.16804 | 1.15358 | 1.51953 | 1.87182 | 1.85736 | 2.22331 | 1.4257 | 1.41124 | 1.77719 | 1.82264 | 1.80818 | 2.17413 | 2.73225 | 2.71779 | 3.08374 | 1.72438 | 1.70992 | 2.07587 | 2.3559 | 2.34144 | 2.70739 |
| 96726\_at |  |  | 104381 |  |  |  |  | 5.42435 | 5.89455 | 5.91801 | 5.18572 | 5.52467 | 5.65856 | 5.4543 | 5.78405 | 6.1214 | 6.74259 | 7.52567 | 7.20292 | 6.94837 | 7.25044 | 7.264 | 7.87535 | 5.31585 | 5.74579 | 4.58962 | 0.108497 | -0.321442 | 0.834732 | 0.5787 | 0.148761 | 1.30494 | 0.602154 | 0.172215 | 1.32839 | -0.13013 | -0.560069 | 0.596105 | 0.208816 | -0.221123 | 0.935051 | 0.342705 | -0.0872343 | 1.06894 | 0.13845 | -0.291489 | 0.864685 | 0.468201 | 0.0382624 | 1.19444 | 0.805542 | 0.375603 | 1.53178 | 1.42674 | 0.996798 | 2.15297 | 2.20982 | 1.77988 | 2.93605 | 1.88706 | 1.45712 | 2.6133 | 1.63252 | 1.20258 | 2.35875 | 1.93459 | 1.50465 | 2.66083 | 1.94815 | 1.51821 | 2.67439 | 2.5595 | 2.12956 | 3.28573 |
| 96764\_at | Iigp1 | interferon inducible GTPase 1 | 60440 | 18 | 9 | GDP binding  GTP binding  GTPase activity  GTPase activity  cellular\_component unknown  cytokine and chemokine mediated signaling pathway  protein self binding |  | 8.05651 | 8.04501 | 7.60435 | 7.23799 | 9.18318 | 9.75003 | 10.2544 | 10.3394 | 8.91025 | 10.9829 | 10.5027 | 10.3593 | 9.52445 | 10.9983 | 11.8207 | 12.2298 | 9.27251 | 7.92913 | 3.62778 | -1.216 | 0.127381 | 4.42873 | -1.2275 | 0.11588 | 4.41723 | -1.66817 | -0.324785 | 3.97657 | -2.03453 | -0.691142 | 3.61021 | -0.0893369 | 1.25405 | 5.5554 | 0.47752 | 1.8209 | 6.12225 | 0.981859 | 2.32524 | 6.62659 | 1.0669 | 2.41028 | 6.71164 | -0.362261 | 0.981123 | 5.28247 | 1.71041 | 3.05379 | 7.35514 | 1.23018 | 2.57357 | 6.87492 | 1.08677 | 2.43016 | 6.73151 | 0.251933 | 1.59532 | 5.89667 | 1.72581 | 3.06919 | 7.37054 | 2.5482 | 3.89158 | 8.19294 | 2.95729 | 4.30067 | 8.60203 |
| 96765\_at | Peg3 | paternally expressed 3 | 18616 | 7 | 11 | nucleic acid binding |  | 10.3561 | 10.3723 | 10.607 | 10.9968 | 11.3802 | 10.811 | 11.1791 | 11.0131 | 11.5495 | 11.3603 | 11.7305 | 11.5946 | 11.7527 | 11.4729 | 11.3731 | 11.7232 | 5.49618 | 4.12725 | 4.86362 | 4.85987 | 6.22881 | 5.49243 | 4.87612 | 6.24506 | 5.50868 | 5.11082 | 6.47976 | 5.74338 | 5.50066 | 6.8696 | 6.13323 | 5.88402 | 7.25296 | 6.51658 | 5.31482 | 6.68376 | 5.94738 | 5.68294 | 7.05187 | 6.3155 | 5.51696 | 6.8859 | 6.14952 | 6.05328 | 7.42222 | 6.68584 | 5.86408 | 7.23301 | 6.49664 | 6.23431 | 7.60325 | 6.86687 | 6.0984 | 7.46734 | 6.73096 | 6.25647 | 7.6254 | 6.88903 | 5.97674 | 7.34568 | 6.6093 | 5.87694 | 7.24587 | 6.5095 | 6.22698 | 7.59592 | 6.85955 |
| 96894\_at | 1110014L17Rik | RIKEN cDNA 1110014L17 gene | 103694 | 11 | 7 | extracellular space  integral to membrane  intracellular protein transport  membrane  protein carrier activity |  | 6.70638 | 6.53551 | 6.44617 | 7.10333 | 7.0981 | 6.79588 | 7.0782 | 7.0981 | 7.21175 | 7.10768 | 7.90517 | 7.64473 | 7.50232 | 7.53625 | 7.48861 | 7.42156 | 6.7906 | 6.96937 | 6.84613 | -0.0842225 | -0.262995 | -0.139755 | -0.255086 | -0.433859 | -0.310618 | -0.344427 | -0.5232 | -0.399959 | 0.312734 | 0.133961 | 0.257202 | 0.307502 | 0.128729 | 0.25197 | 0.0052783 | -0.173495 | -0.0502537 | 0.287605 | 0.108832 | 0.232073 | 0.307502 | 0.128729 | 0.25197 | 0.421149 | 0.242376 | 0.365617 | 0.317083 | 0.13831 | 0.261551 | 1.11458 | 0.935802 | 1.05904 | 0.854135 | 0.675363 | 0.798603 | 0.711719 | 0.532946 | 0.656187 | 0.745655 | 0.566882 | 0.690123 | 0.698015 | 0.519243 | 0.642483 | 0.630966 | 0.452193 | 0.575434 |
| 96953\_at | Cxcl14 | chemokine (C-X-C motif) ligand 14 | 57266 | 13 | 9 | chemokine activity  cytokine activity  extracellular  extracellular space  immune response | Cytokine-cytokine receptor interaction | 3.43808 | 3.54717 | 3.6152 | 3.51798 | 3.51226 | 3.57043 | 3.59983 | 3.56097 | 5.44729 | 4.78113 | 5.06485 | 4.79884 | 5.56915 | 5.91474 | 4.95506 | 5.88623 | 3.52419 | 3.49562 | 3.42832 | -0.0861146 | -0.0575421 | 0.00975435 | 0.0229775 | 0.05155 | 0.118846 | 0.0910047 | 0.119577 | 0.186874 | -0.0062075 | 0.022365 | 0.0896615 | -0.0119301 | 0.0166424 | 0.0839389 | 0.0462404 | 0.0748129 | 0.142109 | 0.0756368 | 0.104209 | 0.171506 | 0.0367804 | 0.0653529 | 0.132649 | 1.92309 | 1.95167 | 2.01896 | 1.25694 | 1.28551 | 1.35281 | 1.54065 | 1.56923 | 1.63652 | 1.27465 | 1.30322 | 1.37052 | 2.04496 | 2.07353 | 2.14083 | 2.39055 | 2.41912 | 2.48642 | 1.43087 | 1.45944 | 1.52674 | 2.36204 | 2.39061 | 2.45791 |
| 97242\_at | 0610010O12Rik | RIKEN cDNA 0610010O12 gene | 66060 | 18 | 6 | nucleus |  | 3.54228 | 5.72808 | 4.31128 | 4.59215 | 6.01876 | 5.11869 | 5.27699 | 4.45173 | 7.28874 | 6.45786 | 7.39784 | 7.83289 | 8.39759 | 7.38229 | 8.63287 | 7.51117 | 2.72031 | 1.98568 | 2.83559 | 0.821968 | 1.5566 | 0.706687 | 3.00776 | 3.74239 | 2.89248 | 1.59096 | 2.32559 | 1.47568 | 1.87184 | 2.60647 | 1.75656 | 3.29845 | 4.03308 | 3.18317 | 2.39838 | 3.13301 | 2.2831 | 2.55668 | 3.29131 | 2.4414 | 1.73142 | 2.46605 | 1.61614 | 4.56843 | 5.30306 | 4.45315 | 3.73755 | 4.47218 | 3.62227 | 4.67753 | 5.41216 | 4.56225 | 5.11258 | 5.84721 | 4.9973 | 5.67728 | 6.41191 | 5.562 | 4.66198 | 5.39661 | 4.5467 | 5.91256 | 6.64719 | 5.79728 | 4.79086 | 5.52549 | 4.67558 |
| 97409\_at | Ifi1 | interferon inducible protein 1 | 15944 | 11 | 8 | defense response  endoplasmic reticulum |  | 4.66477 | 5.19311 | 4.98846 | 4.78996 | 6.33266 | 5.65487 | 6.43141 | 6.29443 | 5.63836 | 6.92475 | 8.06086 | 7.33426 | 7.23518 | 7.48345 | 8.91735 | 8.95821 | 4.97127 | 4.14397 | 3.6245 | -0.306505 | 0.520799 | 1.04027 | 0.221834 | 1.04914 | 1.56861 | 0.0171852 | 0.84449 | 1.36396 | -0.181312 | 0.645992 | 1.16546 | 1.36139 | 2.18869 | 2.70817 | 0.683595 | 1.5109 | 2.03037 | 1.46013 | 2.28744 | 2.80691 | 1.32316 | 2.15046 | 2.66993 | 0.66709 | 1.49439 | 2.01387 | 1.95347 | 2.78078 | 3.30025 | 3.08959 | 3.91689 | 4.43637 | 2.36299 | 3.1903 | 3.70977 | 2.26391 | 3.09121 | 3.61068 | 2.51217 | 3.33948 | 3.85895 | 3.94608 | 4.77338 | 5.29286 | 3.98694 | 4.81424 | 5.33372 |
| 97415\_at | Rab3d | RAB3D, member RAS oncogene family | 19340 | 9 | 14 | GTP binding  exocytosis  intracellular  protein binding  protein transport  regulation of exocytosis  small GTPase mediated signal transduction  transport  zymogen granule |  | 5.37631 | 5.65963 | 7.30995 | 5.82024 | 7.57114 | 7.21305 | 5.60104 | 5.61246 | 7.88191 | 9.85277 | 10.0138 | 10.5205 | 10.0008 | 10.5384 | 10.72 | 10.808 | 6.55615 | 7.17304 | 7.5019 | -1.17985 | -1.79673 | -2.12559 | -0.896523 | -1.51341 | -1.84227 | 0.753793 | 0.136908 | -0.19195 | -0.73591 | -1.3528 | -1.68165 | 1.01498 | 0.398095 | 0.0692373 | 0.656899 | 0.0400144 | -0.288844 | -0.95511 | -1.572 | -1.90085 | -0.94369 | -1.56057 | -1.88943 | 1.32575 | 0.708869 | 0.380012 | 3.29662 | 2.67973 | 2.35087 | 3.45768 | 2.84079 | 2.51194 | 3.96433 | 3.34744 | 3.01858 | 3.44461 | 2.82772 | 2.49886 | 3.98226 | 3.36538 | 3.03652 | 4.16385 | 3.54696 | 3.2181 | 4.25184 | 3.63495 | 3.30609 |
| 97487\_at | Serpine2 | serine (or cysteine) proteinase inhibitor, clade E, member 2 | 20720 | 1 | 8 | extracellular space  heparin binding  serine-type endopeptidase inhibitor activity |  | 3.40036 | 3.91006 | 3.64693 | 3.44551 | 4.06615 | 3.578 | 4.02314 | 4.06615 | 7.63163 | 7.70526 | 8.49595 | 8.59417 | 7.89454 | 8.77894 | 8.65789 | 8.42397 | 3.38833 | 3.37248 | 3.31726 | 0.0120299 | 0.0278774 | 0.083092 | 0.521734 | 0.537581 | 0.592796 | 0.258603 | 0.274451 | 0.329665 | 0.0571882 | 0.0730357 | 0.12825 | 0.677821 | 0.693668 | 0.748883 | 0.189677 | 0.205524 | 0.260739 | 0.634811 | 0.650659 | 0.705873 | 0.677821 | 0.693668 | 0.748883 | 4.2433 | 4.25915 | 4.31436 | 4.31693 | 4.33278 | 4.388 | 5.10762 | 5.12347 | 5.17869 | 5.20584 | 5.22169 | 5.2769 | 4.50621 | 4.52206 | 4.57727 | 5.39061 | 5.40646 | 5.46168 | 5.26956 | 5.28541 | 5.34063 | 5.03565 | 5.05149 | 5.10671 |
| 97509\_f\_at | Fgfr1 | fibroblast growth factor receptor 1 | 14182 | 8 | 32 | ATP binding  brain development  extracellular space  fibroblast growth factor receptor activity  inner ear morphogenesis  integral to membrane  kinase activity  protein amino acid phosphorylation  protein kinase activity  protein serine/threonine kinase activity  protein-tyrosine kinase activity  receptor activity  salivary gland morphogenesis  signal transduction  transferase activity | MAPK signaling pathway | 7.46362 | 7.1068 | 7.94651 | 7.22207 | 7.80236 | 7.92143 | 7.8791 | 7.45945 | 8.6596 | 8.48887 | 8.74658 | 9.16763 | 8.7878 | 9.8985 | 9.80822 | 10.1238 | 5.91245 | 3.97204 | 4.57732 | 1.55117 | 3.49158 | 2.8863 | 1.19434 | 3.13476 | 2.52947 | 2.03406 | 3.97447 | 3.36919 | 1.30962 | 3.25003 | 2.64475 | 1.8899 | 3.83032 | 3.22503 | 2.00897 | 3.94939 | 3.3441 | 1.96664 | 3.90706 | 3.30177 | 1.547 | 3.48741 | 2.88213 | 2.74715 | 4.68756 | 4.08228 | 2.57642 | 4.51683 | 3.91155 | 2.83413 | 4.77455 | 4.16926 | 3.25518 | 5.1956 | 4.59031 | 2.87535 | 4.81576 | 4.21048 | 3.98605 | 5.92646 | 5.32118 | 3.89577 | 5.83618 | 5.2309 | 4.21138 | 6.1518 | 5.54651 |
| 97519\_at | Spp1 | secreted phosphoprotein 1 | 20750 | 5 | 48 | cell adhesion  cytokine activity  extracellular space  ossification  protein binding |  | 4.0093 | 8.49786 | 8.8768 | 8.53906 | 11.9614 | 10.2822 | 11.9578 | 9.98701 | 13.2624 | 13.1467 | 13.6804 | 13.903 | 13.6362 | 13.6968 | 13.5477 | 13.3709 | 13.2528 | 13.1597 | 12.3502 | -9.24352 | -9.15045 | -8.34088 | -4.75495 | -4.66189 | -3.85232 | -4.37601 | -4.28295 | -3.47338 | -4.71375 | -4.62069 | -3.81112 | -1.29145 | -1.19838 | -0.38881 | -2.97065 | -2.87759 | -2.06802 | -1.29504 | -1.20198 | -0.392408 | -3.26581 | -3.17274 | -2.36317 | 0.00960217 | 0.102669 | 0.912237 | -0.106146 | -0.0130791 | 0.796489 | 0.427628 | 0.520695 | 1.33026 | 0.650211 | 0.743278 | 1.55285 | 0.383349 | 0.476415 | 1.28598 | 0.443978 | 0.537045 | 1.34661 | 0.294854 | 0.387921 | 1.19749 | 0.118126 | 0.211192 | 1.02076 |
| 97811\_at | Arfgap3 | ADP-ribosylation factor GTPase activating protein 3 | 66251 | 15 | 6 | GTPase activator activity  protein transport  regulation of GTPase activity  transport |  | 4.56936 | 4.55645 | 5.13813 | 5.52624 | 6.46799 | 5.43229 | 6.06708 | 5.58927 | 8.72969 | 8.03006 | 9.38725 | 9.53227 | 9.50109 | 9.662 | 10.1986 | 9.90656 | 7.48825 | 6.70562 | 5.84808 | -2.91889 | -2.13626 | -1.27872 | -2.93181 | -2.14918 | -1.29163 | -2.35013 | -1.5675 | -0.70995 | -1.96201 | -1.17938 | -0.321836 | -1.02026 | -0.237629 | 0.619917 | -2.05596 | -1.27333 | -0.415782 | -1.42118 | -0.638544 | 0.219002 | -1.89898 | -1.11635 | -0.258803 | 1.24144 | 2.02407 | 2.88162 | 0.541806 | 1.32444 | 2.18198 | 1.89899 | 2.68162 | 3.53917 | 2.04402 | 2.82665 | 3.6842 | 2.01284 | 2.79547 | 3.65301 | 2.17375 | 2.95638 | 3.81393 | 2.71039 | 3.49302 | 4.35056 | 2.41831 | 3.20094 | 4.05849 |
| 97825\_at | Perp | PERP, TP53 apoptosis effector | 64058 | 10 | 9 | Golgi apparatus  extracellular space  induction of apoptosis  integral to membrane  integral to plasma membrane  mitochondrion |  | 2.4808 | 4.54083 | 5.03988 | 5.25653 | 6.9189 | 4.8585 | 5.06364 | 4.83606 | 7.65486 | 7.24401 | 9.16405 | 8.49221 | 8.78319 | 8.60243 | 8.4562 | 8.73645 | 6.87373 | 6.96885 | 6.89379 | -4.39294 | -4.48805 | -4.413 | -2.33291 | -2.42802 | -2.35296 | -1.83385 | -1.92897 | -1.85391 | -1.61721 | -1.71232 | -1.63726 | 0.0451709 | -0.0499444 | 0.0251132 | -2.01523 | -2.11035 | -2.03529 | -1.81009 | -1.90521 | -1.83015 | -2.03767 | -2.13279 | -2.05773 | 0.781132 | 0.686017 | 0.761074 | 0.370273 | 0.275158 | 0.350216 | 2.29032 | 2.19521 | 2.27026 | 1.61848 | 1.52336 | 1.59842 | 1.90945 | 1.81434 | 1.8894 | 1.72869 | 1.63358 | 1.70863 | 1.58247 | 1.48735 | 1.56241 | 1.86272 | 1.7676 | 1.84266 |
| 97871\_at | Ero1l | ERO1-like (S. cerevisiae) | 50527 | 14 | 7 | electron transport  endoplasmic reticulum  integral to endoplasmic reticulum membrane  membrane  oxidoreductase activity  protein folding  transport |  | 3.39768 | 3.09952 | 2.77242 | 3.35103 | 4.06182 | 3.07551 | 4.09337 | 3.81844 | 4.77809 | 5.7911 | 8.13114 | 7.6336 | 7.12803 | 6.62032 | 7.34391 | 5.76278 | 3.07272 | 4.10032 | 3.61084 | 0.324957 | -0.702638 | -0.213162 | 0.0267943 | -1.0008 | -0.511325 | -0.300299 | -1.32789 | -0.838418 | 0.278308 | -0.749288 | -0.259811 | 0.989101 | -0.0384942 | 0.450982 | 0.00278898 | -1.02481 | -0.53533 | 1.02065 | -0.00694945 | 0.482527 | 0.745715 | -0.28188 | 0.207596 | 1.70537 | 0.67777 | 1.16725 | 2.71837 | 1.69078 | 2.18025 | 5.05842 | 4.03082 | 4.5203 | 4.56088 | 3.53328 | 4.02276 | 4.05531 | 3.02771 | 3.51719 | 3.5476 | 2.52001 | 3.00948 | 4.27118 | 3.24359 | 3.73306 | 2.69005 | 1.66246 | 2.15193 |
| 97933\_at | 2300006M17Rik | RIKEN cDNA 2300006M17 gene | 69466 | 13 | 6 |  |  | 2.11489 | 2.16834 | 2.21056 | 2.12274 | 2.20494 | 2.18391 | 2.20353 | 2.19287 | 6.95583 | 7.24437 | 7.9672 | 7.8349 | 7.61089 | 7.00494 | 8.27017 | 7.20677 | 2.09342 | 2.1073 | 2.06672 | 0.0214703 | 0.00759124 | 0.0481651 | 0.0749175 | 0.0610384 | 0.101612 | 0.117142 | 0.103263 | 0.143837 | 0.0293226 | 0.0154435 | 0.0560173 | 0.111524 | 0.0976446 | 0.138218 | 0.0904904 | 0.0766113 | 0.117185 | 0.110111 | 0.0962318 | 0.136806 | 0.0994466 | 0.0855675 | 0.126141 | 4.86241 | 4.84853 | 4.8891 | 5.15095 | 5.13707 | 5.17765 | 5.87378 | 5.8599 | 5.90048 | 5.74148 | 5.7276 | 5.76818 | 5.51747 | 5.50359 | 5.54416 | 4.91152 | 4.89764 | 4.93822 | 6.17675 | 6.16287 | 6.20345 | 5.11335 | 5.09947 | 5.14004 |
| 97970\_at | Mfi2 | antigen p97 (melanoma associated) identified by monoclonal antibodies 133.2 and 96.5 | 30060 | 16 | 9 | extracellular  extracellular space  ferric iron binding  iron ion homeostasis  iron ion transport  membrane  transport |  | 2.43181 | 2.4899 | 2.53171 | 2.42706 | 2.44097 | 2.5004 | 2.4611 | 2.49964 | 2.79679 | 4.41413 | 7.04306 | 6.86746 | 7.21134 | 7.68432 | 6.76619 | 7.25626 | 2.40107 | 2.40558 | 2.36587 | 0.0307489 | 0.026236 | 0.0659452 | 0.0888333 | 0.0843203 | 0.12403 | 0.130643 | 0.12613 | 0.165839 | 0.0259993 | 0.0214864 | 0.0611957 | 0.0399086 | 0.0353957 | 0.0751049 | 0.0993336 | 0.0948207 | 0.13453 | 0.0600363 | 0.0555234 | 0.0952327 | 0.0985782 | 0.0940653 | 0.133775 | 0.395725 | 0.391213 | 0.430922 | 2.01306 | 2.00855 | 2.04826 | 4.642 | 4.63748 | 4.67719 | 4.46639 | 4.46188 | 4.50159 | 4.81028 | 4.80576 | 4.84547 | 5.28325 | 5.27874 | 5.31845 | 4.36512 | 4.36061 | 4.40032 | 4.85519 | 4.85068 | 4.89039 |
| 97997\_at | Sfrp1 | secreted frizzled-related sequence protein 1 | 20377 | 8 | 8 | development  extracellular space  membrane  transmembrane receptor activity | Wnt signaling pathway | 3.3042 | 3.39967 | 3.44073 | 3.29796 | 3.42489 | 3.40994 | 3.52021 | 3.40439 | 7.03871 | 6.66375 | 8.22822 | 7.32521 | 8.18876 | 8.42586 | 7.94373 | 8.2767 | 3.27871 | 3.27021 | 3.2405 | 0.025491 | 0.0339922 | 0.0637052 | 0.120955 | 0.129456 | 0.159169 | 0.162015 | 0.170517 | 0.20023 | 0.019251 | 0.0277522 | 0.0574652 | 0.146176 | 0.154677 | 0.18439 | 0.131229 | 0.139731 | 0.169444 | 0.2415 | 0.250001 | 0.279714 | 0.12568 | 0.134181 | 0.163894 | 3.76001 | 3.76851 | 3.79822 | 3.38504 | 3.39354 | 3.42326 | 4.94951 | 4.95801 | 4.98772 | 4.0465 | 4.055 | 4.08472 | 4.91005 | 4.91855 | 4.94826 | 5.14715 | 5.15565 | 5.18536 | 4.66502 | 4.67352 | 4.70323 | 4.99799 | 5.00649 | 5.0362 |
| 98071\_f\_at | Dck | deoxycytidine kinase | 13178 |  | 6 | ATP binding  deoxycytidine kinase activity  kinase activity  nucleobase, nucleoside, nucleotide and nucleic acid metabolism  nucleus  phosphotransferase activity, alcohol group as acceptor  transferase activity | Purine metabolism  Pyrimidine metabolism | 4.65066 | 3.66477 | 3.30191 | 2.4191 | 3.4435 | 3.02983 | 5.29589 | 5.09397 | 7.76419 | 8.69824 | 8.97256 | 8.22364 | 6.52069 | 9.18461 | 8.86982 | 8.75674 | 1.54258 | 1.46948 | 1.585 | 3.10807 | 3.18117 | 3.06565 | 2.12219 | 2.19529 | 2.07977 | 1.75932 | 1.83242 | 1.7169 | 0.876517 | 0.949616 | 0.834096 | 1.90092 | 1.97401 | 1.8585 | 1.48724 | 1.56034 | 1.44482 | 3.7533 | 3.8264 | 3.71088 | 3.55138 | 3.62448 | 3.50896 | 6.22161 | 6.29471 | 6.17919 | 7.15566 | 7.22876 | 7.11324 | 7.42998 | 7.50308 | 7.38756 | 6.68105 | 6.75415 | 6.63863 | 4.97811 | 5.05121 | 4.93569 | 7.64202 | 7.71512 | 7.5996 | 7.32724 | 7.40034 | 7.28482 | 7.21416 | 7.28725 | 7.17174 |
| 98088\_at | Cd14 | CD14 antigen | 12475 | 18 | 19 | extracellular space  immune response  inflammatory response  plasma membrane | MAPK signaling pathway  Toll-like receptor signaling pathway | 4.47322 | 5.47581 | 7.14214 | 7.00441 | 8.53561 | 6.52067 | 8.47618 | 5.39071 | 10.0933 | 10.7633 | 10.5233 | 10.78 | 11.0557 | 10.7753 | 11.0515 | 11.324 | 5.74549 | 6.0149 | 5.91416 | -1.27227 | -1.54168 | -1.44094 | -0.269683 | -0.539097 | -0.438356 | 1.39665 | 1.12724 | 1.22798 | 1.25892 | 0.989504 | 1.09025 | 2.79012 | 2.52071 | 2.62145 | 0.775177 | 0.505764 | 0.606505 | 2.73069 | 2.46127 | 2.56201 | -0.354781 | -0.624194 | -0.523453 | 4.34782 | 4.07841 | 4.17915 | 5.01779 | 4.74838 | 4.84912 | 4.77777 | 4.50836 | 4.6091 | 5.03454 | 4.76513 | 4.86587 | 5.31023 | 5.04082 | 5.14156 | 5.02979 | 4.76038 | 4.86112 | 5.30605 | 5.03664 | 5.13738 | 5.57848 | 5.30906 | 5.4098 |
| 98122\_at | Lmo4 | LIM domain only 4 | 16911 | 3 | 13 | neural tube closure  protein binding  regulation of transcription, DNA-dependent  transcription factor binding  transcription factor complex  transcription from Pol II promoter |  | 9.66408 | 9.27593 | 9.49077 | 9.68955 | 10.5283 | 10.0717 | 10.0703 | 9.5603 | 10.6367 | 11.2843 | 11.7651 | 11.5535 | 11.3199 | 11.6351 | 11.7923 | 11.5536 | 10.2924 | 9.96249 | 10.4761 | -0.628325 | -0.298406 | -0.811983 | -1.01647 | -0.686554 | -1.20013 | -0.801631 | -0.471712 | -0.985289 | -0.602851 | -0.272932 | -0.786509 | 0.235904 | 0.565823 | 0.0522453 | -0.220679 | 0.109239 | -0.404338 | -0.222129 | 0.10779 | -0.405787 | -0.732106 | -0.402187 | -0.915764 | 0.344288 | 0.674207 | 0.160629 | 0.991852 | 1.32177 | 0.808193 | 1.4727 | 1.80261 | 1.28904 | 1.26106 | 1.59097 | 1.0774 | 1.02752 | 1.35744 | 0.843866 | 1.3427 | 1.67262 | 1.15905 | 1.49989 | 1.82981 | 1.31623 | 1.2612 | 1.59112 | 1.07755 |
| 98151\_s\_at | Catns | catenin src | 12388 | 2 | 5 | cell adhesion  cytoskeleton  nucleus  protein binding  structural molecule activity |  | 2.52318 | 3.24021 | 3.35469 | 2.48379 | 3.0029 | 2.82778 | 2.69607 | 2.63565 | 3.67072 | 6.54896 | 6.77572 | 7.09237 | 6.27088 | 8.79806 | 8.29377 | 7.25704 | 2.87499 | 2.92736 | 2.49506 | -0.35181 | -0.404177 | 0.0281228 | 0.365216 | 0.312849 | 0.745149 | 0.479704 | 0.427337 | 0.859637 | -0.391202 | -0.443569 | -0.011269 | 0.127908 | 0.0755415 | 0.507841 | -0.0472093 | -0.0995761 | 0.332724 | -0.178922 | -0.231289 | 0.201011 | -0.239344 | -0.291711 | 0.140589 | 0.79573 | 0.743364 | 1.17566 | 3.67397 | 3.62161 | 4.05391 | 3.90073 | 3.84837 | 4.28067 | 4.21738 | 4.16501 | 4.59731 | 3.39589 | 3.34352 | 3.77582 | 5.92307 | 5.8707 | 6.303 | 5.41878 | 5.36641 | 5.79871 | 4.38205 | 4.32968 | 4.76198 |
| 98532\_at | Cdk2ap1 | CDK2 (cyclin-dependent kinase 2)-associated protein 1 | 13445 | 5 | 7 | kinase activity  negative regulation of cell cycle |  | 9.91128 | 9.78762 | 9.72625 | 9.71429 | 9.9826 | 9.75963 | 10.1337 | 9.60646 | 10.4109 | 10.1724 | 10.8642 | 10.7173 | 10.7166 | 10.85 | 11.0588 | 10.7604 | 10.636 | 10.5537 | 10.5605 | -0.724677 | -0.642431 | -0.64926 | -0.848338 | -0.766092 | -0.772922 | -0.909707 | -0.827461 | -0.834291 | -0.921665 | -0.839419 | -0.846248 | -0.653356 | -0.57111 | -0.577939 | -0.87633 | -0.794084 | -0.800914 | -0.502239 | -0.419993 | -0.426823 | -1.02949 | -0.947246 | -0.954076 | -0.225085 | -0.142839 | -0.149668 | -0.463568 | -0.381322 | -0.388151 | 0.228277 | 0.310523 | 0.303693 | 0.0813186 | 0.163565 | 0.156735 | 0.0806423 | 0.162888 | 0.156059 | 0.214023 | 0.296269 | 0.28944 | 0.42284 | 0.505086 | 0.498257 | 0.124485 | 0.206731 | 0.199902 |
| 98593\_at | Cmas | cytidine monophospho-N-acetylneuraminic acid synthetase | 12764 | 6 | 10 | CMP-N-acetylneuraminate biosynthesis  N-acylneuraminate cytidylyltransferase activity  lipopolysaccharide biosynthesis  nucleotidyltransferase activity  nucleus  transferase activity |  | 4.67812 | 4.79011 | 6.3027 | 6.87856 | 8.21179 | 5.55495 | 7.55611 | 6.37658 | 9.22144 | 10.1019 | 10.5481 | 10.7812 | 10.6102 | 10.5753 | 10.4693 | 10.6387 | 6.9019 | 7.70111 | 7.17289 | -2.22378 | -3.02299 | -2.49477 | -2.11179 | -2.911 | -2.38278 | -0.599202 | -1.39841 | -0.870196 | -0.0233403 | -0.82255 | -0.294334 | 1.30989 | 0.510677 | 1.03889 | -1.34695 | -2.14616 | -1.61795 | 0.65421 | -0.144999 | 0.383217 | -0.525323 | -1.32453 | -0.796316 | 2.31954 | 1.52033 | 2.04854 | 3.20004 | 2.40083 | 2.92904 | 3.64617 | 2.84696 | 3.37518 | 3.87935 | 3.08014 | 3.60836 | 3.70828 | 2.90907 | 3.43729 | 3.67341 | 2.8742 | 3.40241 | 3.56736 | 2.76815 | 3.29637 | 3.73683 | 2.93762 | 3.46584 |
| 98944\_at | Sec23b | SEC23B (S. cerevisiae) | 27054 | 2 | 6 | COPII vesicle coat  ER to Golgi transport  Golgi apparatus  endoplasmic reticulum  intracellular protein transport  protein binding  protein transport  transport |  | 5.72114 | 5.94094 | 5.76748 | 5.73846 | 6.18908 | 5.92866 | 6.08485 | 5.78372 | 6.71175 | 6.58766 | 6.9052 | 7.08061 | 6.50674 | 7.13031 | 6.59057 | 6.89522 | 6.57898 | 7.46732 | 8.21421 | -0.857835 | -1.74617 | -2.49307 | -0.638038 | -1.52638 | -2.27327 | -0.811498 | -1.69984 | -2.44673 | -0.840524 | -1.72886 | -2.47576 | -0.389903 | -1.27824 | -2.02514 | -0.650317 | -1.53866 | -2.28555 | -0.494131 | -1.38247 | -2.12937 | -0.795255 | -1.68359 | -2.43049 | 0.132768 | -0.755571 | -1.50247 | 0.00867591 | -0.879663 | -1.62656 | 0.326224 | -0.562115 | -1.30901 | 0.50163 | -0.386709 | -1.13361 | -0.0722347 | -0.960574 | -1.70747 | 0.551333 | -0.337006 | -1.0839 | 0.0115867 | -0.876752 | -1.62365 | 0.316241 | -0.572098 | -1.31899 |
| 99011\_at | Galnt3 | UDP-N-acetyl-alpha-D-galactosamine | 14425 | 2 | 6 | Golgi apparatus  integral to membrane  manganese ion binding  polypeptide N-acetylgalactosaminyltransferase activity  sugar binding  transferase activity  transferase activity, transferring glycosyl groups | O-Glycans biosynthesis | 4.02479 | 4.10228 | 4.26023 | 3.97402 | 4.97803 | 4.18311 | 4.71532 | 4.16314 | 6.9894 | 6.39534 | 7.5896 | 7.61293 | 6.12247 | 8.07481 | 7.70737 | 7.55719 | 4.64695 | 4.6618 | 3.98897 | -0.622159 | -0.637005 | 0.0358211 | -0.544675 | -0.559521 | 0.113305 | -0.386721 | -0.401567 | 0.271259 | -0.672935 | -0.687781 | -0.0149551 | 0.331077 | 0.316232 | 0.989058 | -0.463846 | -0.478692 | 0.194134 | 0.0683685 | 0.0535232 | 0.726349 | -0.483807 | -0.498652 | 0.174173 | 2.34245 | 2.3276 | 3.00043 | 1.74838 | 1.73354 | 2.40637 | 2.94265 | 2.9278 | 3.60063 | 2.96598 | 2.95114 | 3.62396 | 1.47552 | 1.46067 | 2.1335 | 3.42786 | 3.41301 | 4.08584 | 3.06042 | 3.04557 | 3.7184 | 2.91024 | 2.8954 | 3.56822 |
| 99065\_at | Csnk | casein kappa | 12994 | 5 | 7 | extracellular  extracellular space |  | 11.4621 | 11.1858 | 11.9017 | 12.6278 | 13.1862 | 12.8179 | 13.1807 | 12.7969 | 13.2598 | 13.0149 | 13.6793 | 13.7915 | 13.7303 | 13.3475 | 13.8644 | 13.7502 | 13.7538 | 13.5908 | 13.5715 | -2.29171 | -2.12871 | -2.10939 | -2.56798 | -2.40498 | -2.38566 | -1.85211 | -1.68911 | -1.66979 | -1.12597 | -0.96297 | -0.943655 | -0.567632 | -0.404629 | -0.385314 | -0.935853 | -0.772849 | -0.753534 | -0.573067 | -0.410063 | -0.390749 | -0.956898 | -0.793894 | -0.774579 | -0.493959 | -0.330955 | -0.31164 | -0.738917 | -0.575913 | -0.556598 | -0.0745356 | 0.0884682 | 0.107783 | 0.0377469 | 0.200751 | 0.220066 | -0.023465 | 0.139539 | 0.158854 | -0.406323 | -0.243319 | -0.224004 | 0.110591 | 0.273595 | 0.29291 | -0.00363295 | 0.159371 | 0.178686 |
| 99460\_at | Sema3b | sema domain, immunoglobulin domain (Ig), short basic domain, secreted, (semaphorin) 3B | 20347 | 9 | 6 | development  extracellular space  membrane  neurogenesis  receptor activity |  | 3.53716 | 3.59624 | 3.63606 | 3.74772 | 3.96973 | 3.61434 | 3.73656 | 3.64529 | 8.54956 | 8.54675 | 10.3416 | 10.173 | 10.8908 | 10.0487 | 10.248 | 10.2675 | 3.49195 | 3.50708 | 3.47166 | 0.0452097 | 0.0300857 | 0.0654995 | 0.104284 | 0.0891604 | 0.124574 | 0.144112 | 0.128988 | 0.164402 | 0.25577 | 0.240646 | 0.27606 | 0.477781 | 0.462657 | 0.498071 | 0.122383 | 0.107259 | 0.142673 | 0.244611 | 0.229487 | 0.264901 | 0.153339 | 0.138215 | 0.173629 | 5.0576 | 5.04248 | 5.07789 | 5.0548 | 5.03968 | 5.07509 | 6.84967 | 6.83454 | 6.86995 | 6.68108 | 6.66595 | 6.70137 | 7.39885 | 7.38373 | 7.41914 | 6.55672 | 6.5416 | 6.57701 | 6.75607 | 6.74095 | 6.77636 | 6.7755 | 6.76038 | 6.79579 |
| 99475\_at | Socs2 | suppressor of cytokine signaling 2 | 216233 | 10 | 13 | intracellular signaling cascade  positive regulation of neuron differentiation  regulation of body size  regulation of cell growth  signal transduction |  | 6.30051 | 7.8152 | 6.60791 | 8.50064 | 10.8183 | 8.91138 | 10.1487 | 9.53401 | 11.9419 | 11.9793 | 12.7726 | 12.5029 | 12.4223 | 12.6605 | 13.0508 | 12.5869 | 6.49228 | 7.09655 | 5.78638 | -0.191772 | -0.79604 | 0.514132 | 1.32291 | 0.718643 | 2.02882 | 0.115626 | -0.488642 | 0.82153 | 2.00836 | 1.40409 | 2.71426 | 4.32605 | 3.72178 | 5.03196 | 2.41909 | 1.81482 | 3.125 | 3.65637 | 3.05211 | 4.36228 | 3.04172 | 2.43746 | 3.74763 | 5.44958 | 4.84532 | 6.15549 | 5.48698 | 4.88271 | 6.19289 | 6.28032 | 5.67605 | 6.98623 | 6.01062 | 5.40635 | 6.71653 | 5.93 | 5.32573 | 6.63591 | 6.16823 | 5.56397 | 6.87414 | 6.55847 | 5.9542 | 7.26438 | 6.09463 | 5.49036 | 6.80053 |
| 99481\_at |  |  | 11929 |  |  |  |  | 8.02116 | 8.09957 | 7.88786 | 6.03557 | 7.58037 | 8.17751 | 7.99659 | 8.02916 | 9.64943 | 9.90588 | 10.1257 | 11.5568 | 10.5729 | 11.7779 | 12.3861 | 10.5471 | 3.08503 | 3.08205 | 6.4476 | 4.93612 | 4.93911 | 1.57356 | 5.01454 | 5.01752 | 1.65197 | 4.80282 | 4.80581 | 1.44026 | 2.95054 | 2.95353 | -0.412024 | 4.49534 | 4.49833 | 1.13277 | 5.09248 | 5.09546 | 1.72991 | 4.91156 | 4.91455 | 1.54899 | 4.94412 | 4.94711 | 1.58156 | 6.56439 | 6.56738 | 3.20183 | 6.82085 | 6.82384 | 3.45828 | 7.04066 | 7.04365 | 3.67809 | 8.47176 | 8.47475 | 5.10919 | 7.48787 | 7.49086 | 4.12531 | 8.69288 | 8.69587 | 5.33031 | 9.30106 | 9.30404 | 5.93849 | 7.46211 | 7.4651 | 4.09955 |
| 99561\_f\_at | Cldn7 | claudin 7 | 53624 | 11 | 8 | extracellular space  integral to membrane  membrane  structural molecule activity  tight junction |  | 3.79178 | 4.49446 | 5.60849 | 6.02333 | 8.07205 | 5.64761 | 5.47233 | 4.40878 | 9.46044 | 9.17762 | 10.1965 | 10.5148 | 10.0094 | 10.4208 | 10.0931 | 10.3404 | 9.10064 | 9.27282 | 9.43051 | -5.30887 | -5.48104 | -5.63873 | -4.60619 | -4.77836 | -4.93605 | -3.49215 | -3.66432 | -3.82202 | -3.07731 | -3.24949 | -3.40718 | -1.02859 | -1.20077 | -1.35846 | -3.45303 | -3.62521 | -3.7829 | -3.62831 | -3.80048 | -3.95818 | -4.69186 | -4.86403 | -5.02173 | 0.359794 | 0.18762 | 0.0299284 | 0.0769801 | -0.0951947 | -0.252886 | 1.09585 | 0.923677 | 0.765985 | 1.41412 | 1.24195 | 1.08426 | 0.90871 | 0.736536 | 0.578844 | 1.32012 | 1.14795 | 0.990256 | 0.992461 | 0.820286 | 0.662594 | 1.23975 | 1.06758 | 0.909884 |
| 99642\_i\_at | Cpe | carboxypeptidase E | 12876 | 8 | 9 | carboxypeptidase A activity  carboxypeptidase E activity  carboxypeptidase E activity  carboxypeptidase activity  extracellular space  hydrolase activity  insulin processing  metallocarboxypeptidase activity  metallopeptidase activity  proteolysis and peptidolysis  zinc ion binding |  | 9.88167 | 10.4144 | 10.3405 | 9.52049 | 10.891 | 10.2342 | 10.4916 | 10.3244 | 12.7658 | 13.0744 | 13.3079 | 13.3081 | 13.429 | 13.9094 | 13.9921 | 13.4452 | 6.52154 | 6.61345 | 7.13687 | 3.36013 | 3.26823 | 2.7448 | 3.89287 | 3.80096 | 3.27754 | 3.81897 | 3.72707 | 3.20364 | 2.99894 | 2.90704 | 2.38361 | 4.36944 | 4.27754 | 3.75411 | 3.71262 | 3.62071 | 3.09729 | 3.97009 | 3.87819 | 3.35476 | 3.80283 | 3.71093 | 3.1875 | 6.24422 | 6.15232 | 5.62889 | 6.5529 | 6.461 | 5.93757 | 6.78639 | 6.69449 | 6.17106 | 6.78654 | 6.69463 | 6.17121 | 6.90744 | 6.81554 | 6.29211 | 7.38788 | 7.29598 | 6.77255 | 7.47057 | 7.37867 | 6.85524 | 6.9237 | 6.83179 | 6.30837 |
| 99643\_f\_at | Cpe | carboxypeptidase E | 12876 | 8 | 9 | carboxypeptidase A activity  carboxypeptidase E activity  carboxypeptidase E activity  carboxypeptidase activity  extracellular space  hydrolase activity  insulin processing  metallocarboxypeptidase activity  metallopeptidase activity  proteolysis and peptidolysis  zinc ion binding |  | 8.62697 | 9.67597 | 9.38019 | 9.25947 | 10.4947 | 8.79374 | 9.78958 | 9.13216 | 11.5557 | 12.0689 | 12.9253 | 12.7585 | 12.9358 | 13.2846 | 12.6191 | 12.4526 | 4.08575 | 4.31312 | 5.29586 | 4.54122 | 4.31385 | 3.3311 | 5.59022 | 5.36285 | 4.3801 | 5.29444 | 5.06706 | 4.08432 | 5.17372 | 4.94635 | 3.9636 | 6.4089 | 6.18153 | 5.19879 | 4.70799 | 4.48062 | 3.49788 | 5.70383 | 5.47646 | 4.49372 | 5.04641 | 4.81904 | 3.83629 | 7.46998 | 7.24261 | 6.25987 | 7.98313 | 7.75576 | 6.77301 | 8.83958 | 8.6122 | 7.62946 | 8.67279 | 8.44542 | 7.46267 | 8.85008 | 8.62271 | 7.63997 | 9.19883 | 8.97146 | 7.98871 | 8.53338 | 8.306 | 7.32326 | 8.36685 | 8.13948 | 7.15674 |
| 99810\_at | Gpx2-ps1 | glutathione peroxidase 2, pseudogene 1 | 14777 | 7 | 1 |  |  | 1.70855 | 1.77023 | 1.80593 | 1.70052 | 1.72171 | 1.77731 | 1.73624 | 1.77428 | 3.7195 | 7.20975 | 9.26763 | 9.19029 | 10.3971 | 9.82852 | 10.8869 | 10.2856 | 1.6738 | 1.67929 | 1.63861 | 0.034746 | 0.0292511 | 0.0699331 | 0.0964284 | 0.0909336 | 0.131616 | 0.132128 | 0.126633 | 0.167315 | 0.0267226 | 0.0212278 | 0.0619097 | 0.0479139 | 0.0424191 | 0.083101 | 0.103512 | 0.0980171 | 0.138699 | 0.0624438 | 0.0569489 | 0.0976309 | 0.100481 | 0.0949861 | 0.135668 | 2.0457 | 2.0402 | 2.08089 | 5.53595 | 5.53046 | 5.57114 | 7.59383 | 7.58833 | 7.62901 | 7.51649 | 7.51099 | 7.55168 | 8.72334 | 8.71785 | 8.75853 | 8.15472 | 8.14922 | 8.18991 | 9.21307 | 9.20757 | 9.24825 | 8.61184 | 8.60634 | 8.64702 |
| 99926\_at | Pigr | polymeric immunoglobulin receptor | 18703 | 1 | 7 | extracellular space  integral to membrane  receptor activity |  | 0.586924 | 0.635888 | 0.663935 | 0.637403 | 6.17704 | 0.773657 | 5.14621 | 0.711479 | 5.18557 | 7.4682 | 8.02853 | 7.99288 | 7.89153 | 7.78809 | 10.1674 | 10.4633 | 9.59124 | 9.87289 | 9.11353 | -9.00432 | -9.28597 | -8.52661 | -8.95535 | -9.237 | -8.47765 | -8.92731 | -9.20896 | -8.4496 | -8.95384 | -9.23549 | -8.47613 | -3.41421 | -3.69586 | -2.9365 | -8.81758 | -9.09923 | -8.33988 | -4.44503 | -4.72668 | -3.96732 | -8.87976 | -9.16141 | -8.40206 | -4.40567 | -4.68732 | -3.92796 | -2.12304 | -2.40469 | -1.64534 | -1.56271 | -1.84436 | -1.085 | -1.59837 | -1.88001 | -1.12066 | -1.69971 | -1.98136 | -1.222 | -1.80315 | -2.0848 | -1.32544 | 0.576142 | 0.294492 | 1.05385 | 0.872075 | 0.590425 | 1.34978 |

205 Genes
